# Dynamic self-assembly of compartmentalized DNA nanotubes

**DOI:** 10.1038/s41467-021-23850-1

**Published:** 2021-06-11

**Authors:** Siddharth Agarwal, Melissa A. Klocke, Passa E. Pungchai, Elisa Franco

**Affiliations:** 1 Department of Bioengineering, University of California, Los Angeles, CA USA; 2 Department of Mechanical Engineering, University of California, Riverside, CA USA; 3 Department of Mechanical and Aerospace Engineering, University of California, Los Angeles, CA USA; 4 Molecular Biology Institute, University of California, Los Angeles, CA USA

**Keywords:** Synthetic biology, Bioinspired materials, DNA nanostructures, Molecular self-assembly

## Abstract

Bottom-up synthetic biology aims to engineer artificial cells capable of responsive behaviors by using a minimal set of molecular components. An important challenge toward this goal is the development of programmable biomaterials that can provide active spatial organization in cell-sized compartments. Here, we demonstrate the dynamic self-assembly of nucleic acid (NA) nanotubes inside water-in-oil droplets. We develop methods to encapsulate and assemble different types of DNA nanotubes from programmable DNA monomers, and demonstrate temporal control of assembly via designed pathways of RNA production and degradation. We examine the dynamic response of encapsulated nanotube assembly and disassembly with the support of statistical analysis of droplet images. Our study provides a toolkit of methods and components to build increasingly complex and functional NA materials to mimic life-like functions in synthetic cells.

## Abstract (web summary)

A major goal in Engineering Biology and Materials Science is the development of active, autonomous scaffolds that mimic those present in biological cells. Here the authors report a toolkit for programming the dynamic behaviour of nucleic acid scaffolds in minimal cell-like compartments.

## Introduction

Synthesis of dynamic, programmable molecular scaffolds is an important challenge towards the development of living materials and artificial cells^1,2^. In biological cells, the cytoskeleton plays an active role in transporting components, determining the cell’s mechanical properties, and coordinating division and motility. While the cytoskeleton is primarily composed of filaments (including actin and microtubules), its operation is orchestrated by a large number of organizing proteins and interactions with the cell membrane^3^. The development of scaffolding systems that are inspired by the cytoskeleton’s architecture promises to endow synthetic cells and materials with the capacity to adapt, partition, and move^4–6^. These scaffolding systems should be easy to customize, and exploit components already established in cell-free synthetic biology.

The most direct approach to build minimal scaffolds inside artificial cells is that of isolating relevant cellular biomolecules and reconstituting them in cell-sized compartments^5,6^. Native cytoskeletal filaments have been encapsulated in a variety of droplets or vesicles; however, achieving dynamic behaviors beyond assembly in confinement is challenging due to both restrictive environmental conditions and laborious purification and reconstitution protocols^7–11^. Encapsulated full cytoplasmic extracts are able to generate assembly, disassembly, and contraction of actomyosin networks^8,12,13^. The inclusion of motor proteins (as well as ATP production and oxygen scavenging) is key to achieve contraction and directed motion^14^. These advances point to exciting opportunities toward harnessing native cytoskeletal systems in synthetic cells. However, the numerous components of the cytoskeleton coevolved with high levels of cross-talk, and these interactions are often not measurable. This makes it difficult to identify the minimal number of components as well as the expression levels needed to achieve a target behavior^5^.

To circumvent the complexity of reconstituting the cytoskeleton, synthetic hydrogels and polymers have been developed as scaffolds for artificial protocells^4,15^. These scaffolded compartments have been engineered to have a multi-layer architecture and to respond to specific chemical inputs as well as to temperature and light^16,17^. However, as they are primarily developed for applications like drug delivery and cosmetics, these scaffolds are not optimized to interact with out-of-equilibrium chemical reactions, nor with active molecular processes and genetic parts that require cytoplasmic conditions.

Nucleic acid (NA) nanotechnology has demonstrated a multitude of scaffolds and dynamic circuits, built by programming a finite number of DNA or RNA molecules^18,19^. NA molecules are rationally designed to match prescribed structural or temporal patterns by assigning complementary domains that bind according to Watson–Crick–Franklin base-pairing rules. The sequences in each domain are typically optimized through computer algorithms, which make it possible to generate a variety of structurally or functionally identical components with distinct sequences^20^. While there are many approaches to building NA structures, methods based on assembly of tiles have produced a variety of synthetic filaments structurally comparable to actin filaments and microtubules^21–24^. DNA or RNA tiles interact via engineered single-stranded sticky-end domains and form micrometer-sized nanotubes with prescribed tiling patterns^21–24^. These nanotubes can in turn be seeded, capped, and spatially organized by DNA structures folded with the origami approach, to build cytoskeletal-like networks^25–27^. In parallel, NA nanotechnology has also developed methods to build molecular circuits with programmable logic and dynamic behaviors operating in vitro and in vivo^28–30^. By exploiting enzymatic production and degradation of RNA, these circuits can recapitulate biological gene networks and respond to a variety of organic and inorganic signals sensed by NA aptamers^31–33^. As NA structural elements, circuits, and sensors share the same base-pairing rules to encode their interactions, all these devices can be modularly interconnected. In particular, DNA nanotubes were modified to respond to the release of NA molecules from NA circuits and sensors: dynamic assembly and disassembly of the nanotubes was controlled in a predictable way by pulse generating circuits, oscillators, and chemical signals such as pH^34–36^. These examples indicate that NA structures, circuits, and sensors may be collectively used to build modular mimics of dynamic cytoskeletal filaments inside artificial cells.

The operation of NA devices in cell-sized compartments has been demonstrated with a particular focus on reaction networks. Out of equilibrium NA circuits that comprise enzymes have been encapsulated in water-in-oil droplets to characterize the circuit bifurcation diagram as well as the robustness of its dynamics to partitioning noise^37–39^. Synthetic transcriptional networks operating in cell-free extracts were also engineered to generate patterns in communicating droplets^40^. Enzyme-free DNA circuits were encapsulated in proteinosomes and used for spatially organized protocell computation^41^. DNA nanostructures have been successfully engineered to serve as synthetic membrane receptors, scaffolding membrane elements, and multi-cell organizers^42–45^. However, little attention has been dedicated to encapsulation and growth of DNA scaffolds inside droplets and vesicles. Two dimensional DNA motifs have been encapsulated inside droplets to increase mechanical stability of vesicles or sense pH. Rapid folding of another two-dimensional DNA origami was confirmed by subsequent extraction, however, studies of DNA scaffold assembly and operation inside compartments are still lacking^46–48^. More importantly, the encapsulation of a multicomponent NA system comprising circuits and scaffolds has not been explored.

In this work, we demonstrate the encapsulation of a modular NA toolkit to build a minimal dynamic scaffolding system for synthetic cells. This toolkit includes DNA nanotubes as a self-assembling scaffold and transcriptional processes to control nanotube assembly and disassembly. We develop a variety of assays for encapsulating DNA nanotubes in cell-sized, water-in-oil droplets, highlighting that multiple nanotube species can be assembled and can coexist. We employ two distinct nanotube designs to control the start of assembly, and characterize the kinetics of polymerization inside compartments using quantitative statistical analysis. By implementing a DNA-RNA hybrid nanotube design, we demonstrate enzyme-mediated control of assembly and disassembly that yields transient presence of nanotubes inside compartments. The density of assembled nanotubes, as well as their lifetime in the droplets, are tunable properties of this system. The methods and components characterized here are a first step toward the bottom-up development of NA cytoskeletal mimics for synthetic minimal cells. Because of the modularity of NA components, this toolkit could be enriched with NA sensors to encode responses to external stimuli, and more complex dynamic networks for autonomous behaviors. Further, the encapsulation of NA structures and condensates promoting the formation of higher-order nanotube architectures would make it possible to systematically specify the overall internal organization and the mechanical properties of protocells.

## Results

### DNA nanotubes self-assemble from preformed monomers at constant temperature

**Fig. 1:**
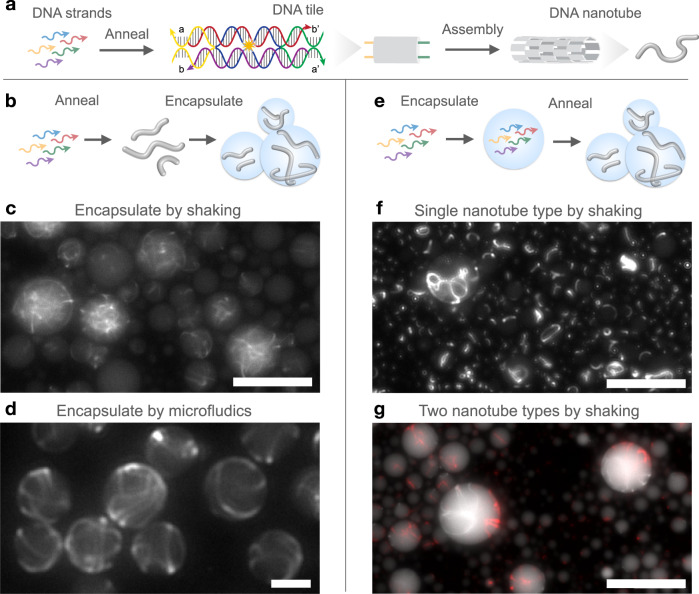
Schematic of DNA tiles and nanotubes and different methods for encapsulating and assembling DNA nanotubes in water-in-oil droplets. **a** DNA tiles composed of five unique ssDNA oligomers (shown in blue, red, yellow, green, and purple strands) that self-assemble into DNA nanotubes. Assembly occurs via hybridization of single-stranded complementary domains known as sticky-ends (marked as a, complementary to a′, and b complementary b′). Tiles are labeled with a fluorescent molecule, indicated here by the yellow star, for easy observation of DNA nanotubes under fluorescence microscopy. **b** DNA nanotubes can be pre-annealed from constituent oligomers in vitro before encapsulation in water-in-oil droplets. Representative fluorescence microscopy images of pre-annealed DNA nanotubes inside droplets using two different encapsulation protocols. **c** Nanotubes encapsulated via the shaken protocol at room temperature. Tile concentration is 500 nM. **d** Nanotubes encapsulated via the microfluidic protocol at room temperature. Tile concentration is 350 nM. **e** Constituent oligomers can be encapsulated inside the droplets and later annealed to form nanotubes. Multiple species of nanotubes can be simultaneously annealed in the droplets if the required strands are present in the initial encapsulated solution. **f** Representative fluorescence microscopy image of a single nanotube species annealed inside droplets (250 nM tile concentration) and then imaged at room temperature. **g** Example fluorescence image of two distinct species of DNA nanotubes annealed inside droplets (250 nM each tile), labeled with Cy3 dye (white) and Atto647N dye (red), respectively and then imaged at room temperature. Scale bars: 30 μm.

NA nanotechnology offers many options to build filamentous structures through different assembly pathways^22,24,49^. Because our goal is to build an artificial scaffolding system with the capacity to assemble and disassemble in the absence of thermal treatment, we selected a class of DNA nanotubes that polymerize at constant temperature from pre-annealed tiles or monomers^50^. Our tiles consist of five strands that form two parallel heteroduplexes held together at two points where strands cross over (double-crossover, or DX) from one duplex to the other, and are known as DAE-E tiles (Fig. 1a)^51^. As DX tile variants differ by the orientation of DNA strands and by the crossover distances, the “DAE-E” acronym describes precisely the tile structure, indicating the number of crossovers (double), the orientation of the strands through the crossover (antiparallel), the number of half-turns between intramolecular crossovers (even), and the number of half-turns between intermolecular crossovers (even).

Two strands (yellow and green in Fig. 1a, on opposite sides of the tile) include sticky-end domains that allow tiles to recognize complementary domains present on other such tiles to self-assemble into a nanotube. By designing the sequences of bases in the sticky-end domains we can program tile interactions and tile assembly in a modular manner. An important advantage of this class of DNA tiles is that pre-annealed tiles bind to each other, forming nanotubes in a range of constant temperatures dependent on the melting temperatures of the sticky-ends (typically between 25–40 °C)^50^. Tile nucleation and polymerization, and thus the yield of nanotubes, are also influenced by other conditions like tile concentration, level of positive cations, and presence of certain enzymes, but such conditions have been abundantly studied in the literature making DX tiles an ideal platform to develop synthetic scaffolds. Nanotube growth is typically monitored via epifluorescence microscopy, by labeling one of the tile strands with a fluorescent dye^22,50^. Here, we take advantage of several well-characterized DAE-E tile variants to explore and develop methods to encapsulate DNA nanotubes with distinct assembly pathways.

### Encapsulation of DNA nanotubes in micrometer sized droplets

Water-in-oil droplets are a simple approach to generate isolated compartments with high-throughput and with size that ranges from a few microns to tens of microns in diameter. We selected a droplet system, consisting of a fluorinated oil and biocompatible-surfactant mixture, and an aqueous medium containing nanotube components and buffer^52^. The surfactant stabilizes the aqueous droplets within the oil/surfactant medium, prevents coalescence and transfer of materials from droplet to droplet, minimizes adsorption of DNA to the surface, and confers resilience to temperature fluctuations^37,53^. In this way, each droplet is an isolated environment that remains stable and can be stored for days at room temperature. While these water-in-oil droplets do not fully mimic the cellular environment, which can freely exchange resources with the surrounding aqueous media, they make it possible to monitor assembly of DNA nanotubes in confined, cell-size compartments for extended periods of time.

In our first series of experiments, we encapsulated nanotubes that assemble from a single fluorescently tagged tile (a single set of five unique strands)^22,35^. This class of tiles folds and then self-assembles into nanotubes during the annealing process, unless the sample is stored at a temperature above the sticky-end melting temperature. First, we explored the encapsulation of nanotubes that were pre-annealed (Fig. 1b–d) using two techniques: a “shaken” protocol and a microfluidic protocol (Supplementary Note S4.7 and S4.8).

The shaken droplet protocol is rapid, requires little expertise, and employs only a bench vortexer to emulsify the oil and liquid phase and generate surfactant-stabilized water-in-oil droplets (Fig. 1c). These droplets present a wide range of diameters, and their size may only be controlled via filtration. Droplet samples were transferred to Ibidi imaging chambers for observation as described in S4.7–9.

While we successfully encapsulated pre-annealed nanotubes with the shaken protocol, we qualitatively observed major variability in the number of nanotubes per droplet, which could be the result of partitioning noise^37^. In sparsely populated droplets, nanotubes do not exhibit unpredicted morphologies or disordered joining. In densely populated droplets, nanotubes appear tightly entangled. It is probable that shear forces during the encapsulation process cause nanotubes to fragment, or promote formation of defects and aggregation in the assemblies. Nanotubes appear to be about as long as the diameter of their confining droplet, or much shorter, although precise measurements of nanotube length are not possible, as is discussed later in the manuscript.

The adoption of a microfluidic chip allows for formation of controlled/discrete sizes of droplets and for reduction of partitioning noise and damage to the nanotubes during encapsulation (Fig. 1d). Nanotubes encapsulated via microfluidics were much more uniformly distributed throughout the droplets than those encapsulated with the shaken protocol. Additionally, nanotubes appear to be longer than the diameter of the droplets confining them, causing them to wrap around the interior surface of the droplets, rather than form a tangled mesh in the center of the droplets as seen for nanotubes encapsulated with the shaken protocol. These results suggest that encapsulation of annealed nanotubes using microfluidics is less destructive than using the shaken method, yet this approach is significantly more laborious than the shaken protocol and requires at least 15 minutes to encapsulate samples. To monitor early nanotube assembly reactions in droplets, we employed the shaken droplet protocol for the remainder of experiments within the paper.

To continue using the rapid shaken protocol while avoiding damaging pre-annealed nanotubes during the process, we developed methods to encapsulate constituent strands inside droplets and subsequently anneal the nanotubes (Fig. 1e). This is feasible because these droplets remain stable at high temperatures. We first encapsulated, and then annealed the five unique strands of a single tile (Fig. 1f), observing assembly of nanotubes that were qualitatively comparable in length and morphology to pre-annealed nanotubes encapsulated via microfluidics. Because structurally identical DNA tiles can be designed to include orthogonal (noninteracting) sequences, it is possible to build distinct tile populations that assemble into distinct nanotubes in the same environment. Thus in our next experiments, we encapsulated the constituent strands of two orthogonal single-tile nanotubes, labeled with different fluorophores. Droplets with the encapsulated tile variants were then annealed, and we observed assembly of nanotube populations presenting different colors (Fig. 1g).

Although achievable, annealing nanotubes inside droplets introduced some difficulties. First, we observed evaporation of a portion of droplets; evaporation can be mitigated by covering samples with a protective layer of hexadecane or water, which however makes it difficult to extract nanotube-containing droplets. Further, constituent strands for some nanotube designs labeled with alternative fluorescent dyes aggregate towards the surface of droplets without assembling into nanotubes after annealing, likely due to interactions between the droplet surface and tiles labeled with hydrophobic dyes.

In addition, it is desirable to develop synthetic scaffolds with the capacity to assemble and disassemble at a constant temperature, like cytoskeletal filaments. For these reasons, we sought to work with alternative tile designs that allow for assembly within droplets without annealing, and we developed methods to aggregate information about assembly in sets of droplets.

### Isothermal growth of an encapsulated two-tile nanotube design

The potential usefulness of artificial biomolecular scaffolds goes beyond introducing a spatial organization within compartments. Like cellular scaffolds, which adapt to stimuli by assembling and disassembling dynamically, a synthetic scaffold could provide temporal control of compartment properties. To achieve this potential, it is important to identify methods enabling assembly at specific times and to assess the kinetics of assembly within the compartment.

**Fig. 2:**
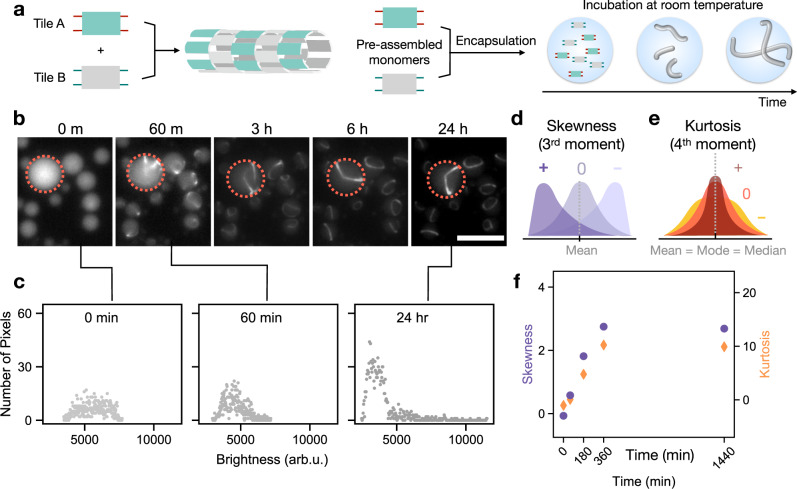
Statistical analysis of droplet fluorescence microscopy images makes it possible to track the condensation of nanotubes in a population of droplets. **a** The two-tile nanotube design requires both tile A and B (shown in green and gray), each with sticky-ends complementary to those of the other tile, for nucleation and polymerization of nanotubes. The tiles are annealed in vitro separately and mixed immediately before encapsulation. **b** Temporal sequence of representative fluorescence microscopy images of droplets encapsulating the two-tile nanotubes at room temperature (each tile at 100 nM). **c** We examine the distribution of pixel brightness across a single droplet over time. As nanotubes assemble, a change in the shape of the distribution also occurs. Each plot shows the histogram of pixel brightness within the droplet of interest at the associated time. Data for this illustrative figure were extracted by hand using ImageJ. **d** Skewness is a measure of the asymmetry of the distribution of a random variable about its mean. **e** Kurtosis is a measure of the “tailedness” of the distribution of a random variable. **f** The skewness and kurtosis values (represented as purple dots and orange diamonds, respectively) increase as nanotubes start growing and becoming more apparent over time inside the droplet. This graph represents a single droplet only. Source data for this figure is provided as a source data file. Scale bar: 20 μm.

To begin to address these challenges, we sought to encapsulate a nanotube design that requires the simultaneous presence of two distinct, interacting tiles (Fig. 2a)^22,54^. The sticky-ends are designed so that complementary sequences to sticky-ends of Tile A are present on Tile B, thus self-assembly can proceed only if both species are present. Tiles A and B can be separately pre-annealed and stored as monomers. Once A and B tiles are mixed and rapidly encapsulated with the shaken protocol (which takes only a couple of minutes from the time of mixing the two tiles), nanotube assembly can be monitored from the early stages of assembly (Fig. 2b). Fluorescence microscopy images do not reveal discernable structures immediately after encapsulation. But within 1 h of incubation at room temperature, we see tubular structures inside the droplets. We also observe that as time progresses, the nanotubes appear to elongate and join (Supplementary Movie 1). Encapsulated assemblies are stable for over 72 h at room temperature (Supplementary Fig. S67). In some cases, nanotubes form rings, a behavior also observed in droplet-encapsulated actin filaments and microtubules^7,55^. It is likely that the rings observed in droplets are actually formed by nanotube bundles. They appear to be larger and less curved than loose rings formed by nonencapsulated DNA nanotubes, whose measured persistence length is 4–5 µm^22,56^. Bundling and localization of nanotubes near the droplet surface were confirmed using confocal microscopy (Supplementary Fig. S11). Bundling may be due to spurious interactions between assembled tiles or between the hydrophobic dyes and the surface, or to confinement effects, as nanotube bundles appear to be longer than the diameter of the droplets. While aggregation of nonencapsulated nanotubes has been observed, it is unclear whether ordered bundles can form in the absence of confinement. We note that addition of DNase to the aqueous phase during encapsulation fully suppresses nanotube growth, while addition of DNase to the oil phase has no effect on nanotube growth because this type of emulsion does not support protein exchange (Supplementary Note S5.17).

### Tracking nanotube assembly through statistical properties of droplet images

While qualitative observations of assembly can be directly made from visual inspection of epifluorescence microscopy images, we sought a less subjective method to track assembly (Fig. 2b). Non-encapsulated nanotubes are typically imaged on glass slides, and statistics about their number and length can be collected with automated image processing^35^. However, this is impractical, if not impossible, inside water-in-oil droplets due to nanotubes intersecting, bundling, and moving within the droplets during confocal microscopy. One could measure the total length of visible segments evident in the microscopy images to get a sense of the polymerization of encapsulated nanotubes at a particular time. This is time consuming, as common methods of separating objects of interest from backgrounds fail to effectively separate nanotubes from background fluorescence in the droplet. In addition, it is still not an accurate reflection of nanotube length, as the total length of visible contours decreases beyond 180 min which disagrees with previous reports on nanotube polymerization kinetics (Supplementary Note S5.16)^35,36^. An alternative route is the examination of statistical measures of droplet epifluorescence microscopy images: the shape of the distribution of pixel intensities within a single droplet clearly evolves over time as nanotubes are qualitatively observed to grow (Fig. 2c). Before nanotubes have polymerized, tiles and corresponding bound fluorescent molecules will be evenly dispersed over the entire volume of a droplet. This uniform spread of tiles, and thus fluorescent signal, will redistribute as tiles are recruited during nanotube polymerization, resulting in distinct bright and dark pixels where there are and are not nanotubes within the droplet, respectively. It is worth noting that the epifluorescence microscopy images we are using to observe nanotube polymerization are a two-dimensional projection of fluorescent signals from within the three-dimensional droplet, with planes above and below the focal plane contributing unfocused signal to the overall image. Thus, one way to assign a quantitative measurement to assembly of nanotubes in individual droplets, is to examine the shape of the distribution of pixel brightness.

Skewness and kurtosis, also respectively known as third and fourth standardized moments, are measures that describe the shape of a distribution. Skewness describes the distribution of any variable about its mean, while kurtosis describes the “tailedness” of a distribution (Fig. 2d, e)^57–59^. Because these are statistical measures that describe the shape of a distribution, they are agnostic to differing exposure times provided that no pixels of the camera used during imaging are saturated, and all pixel brightness values for an image fall within the dynamic range of the camera (Supplementary Note S5.8). Skewness and kurtosis have been previously used to quantify the temporal evolution of actin polymerization and of phase separation of liquid crystals^60,61^.

As nanotubes assemble, the distribution of intensities within a droplet shifts from being symmetric about the mean brightness value to a bulk of the pixels becoming darker while a small number remains bright. This is reflected in the skewness value progressing from near zero at the start of the experiment, before nanotubes have polymerized, to increasing in positive magnitude as nanotubes polymerize. For unpolymerized nanotubes, we expect a negative or near zero kurtosis value, reflecting the heavily tailed distribution of pixel values, which increases to a positive value as nanotubes have polymerized and the distribution shifts to a high peak with weak tails. Indeed, as nanotubes assemble we observe a progressive increase in the skewness and kurtosis values for the distribution of pixel intensities within a single droplet, with a greater relative change in the skewness (Fig. 2f).

Skewness and kurtosis are influenced not only by assembly of nanotubes, indicated by a condensation in the total fluorescent signal but also by the number of free fluorescently-labeled tiles creating a background signal. This is visible by comparing the images and pixel brightness plots in Fig. 2b at 60 minutes and 3+ hours. At both timepoints, nanotubes are visibly polymerized in the droplet, but the amount of free tiles contributing to background noise are different, which is reflected in the shape of the pixel brightness profiles. For this reason, skewness and kurtosis are not a direct measurement of the presence of nanotubes, rather they provide a qualitative picture of condensation through quantitative measurements of statistical properties of droplet images. Skewness and kurtosis measured at a given time point appear to be independent of droplet size (Supplementary Note S5.11), thus we opted for not binning droplets by radius in our analysis.

To track and compare nanotube growth in populations of droplets, we collected skewness and kurtosis measurements for a subpopulation of droplets within the field-of-view, and we report their mean and standard deviation. A larger standard deviation may be taken as an indication of unequal encapsulation of reagents during the shaken droplet protocol. To automate image processing, we also developed a droplet detection code using Python to find droplets and extract their pixel intensities (Supplementary Note S4.13)^62^. A random sample of droplets at each time-point is measured and individual droplets are not tracked through the duration of each experiment. The number of droplets considered for each experiment is shown in the supplementary information, with further discussion on the detection and data extraction process (Supplementary Note S5.9). Unless otherwise noted, data were gathered using the automated droplet detection code.

### Varying concentration of nanotube components and introduction of crowding agents affect morphology and assembly kinetics

In live cells, the assembly and disassembly of cytoskeletal filaments is driven by the concentration of activated protein monomers. Similarly, the concentration of tiles encapsulated in our droplets should influence nanotube assembly. We illustrate this idea with a computational tile assembly model reported in Supplementary Note S6.1. The model, based on ordinary differential equations (ODEs), shows that the higher the tile concentration, the faster nanotube nucleation and elongation reach completion^63^. Expecting to observe similar results in droplet experiments, we encapsulated different concentrations of the two-tile system (Fig. 2a) and continuously monitored nanotube assembly. To prepare the monomers Tile A and Tile B, stoichiometric quantities of the respective five constituent strands were mixed together in TAE buffer containing 12.5 mM Mg^2+^ and annealed (1 °C/min) from 95 °C to room temperature. Pre-annealed Tile A and Tile B were introduced in the aqueous phase simultaneously before encapsulation. Incubation and subsequent measurements were performed immediately after mixing at room temperature.

**Fig. 3:**
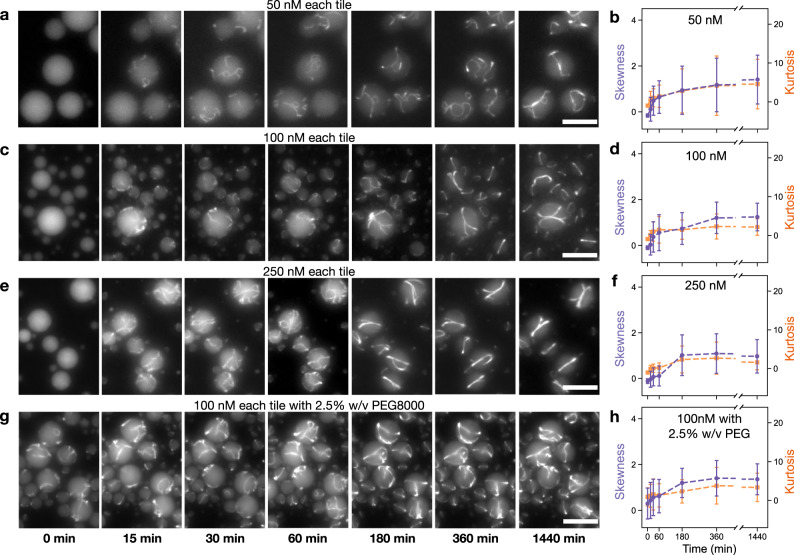
Isothermal assembly of encapsulated two-tile DNA nanotubes at room temperature. Tiles were pre-annealed and mixed immediately before encapsulation. **a**, **c**, **e** Representative temporal sequence of fluorescence microscopy images of two-tile nanotubes encapsulated at 50, 100, and 250 nM concentration for each tile. **b**, **d**, **f** Plots of the mean skewness (purple) and kurtosis (orange). **g** Representative images of two-tile nanotubes encapsulated at 100 nM each tile, with 2.5% w/v PEG. **h** Plot of the mean skewness (purple) and kurtosis (orange) over time for isothermal assembly of tiles encapsulated with PEG. Data were presented as mean values ±  standard deviation. Data extracted using droplet detection code as described in Supplementary Note S4.13. Number of droplets sampled and histogram data of radii for sampled droplets in Supplementary Notes S5.9 and S5.10. Source data for this figure is provided as a source data file. Scale bars: 20 μm.

We monitored encapsulated two-tile nanotubes at 50, 100, and 250 nM each tile for over 24 h (Fig. 3a–f). (For comparison, example images of nonencapsulated nanotubes are in Supplementary Fig. S66.) Qualitatively, small nanotubes were discernible within an hour for all the samples. At 15 and 30 minutes nanotubes appear to be more numerous and longer in the 250 nM tile sample. At 24 h, nanotubes in all samples appear to have elongated, joined, and formed circular bundles that are qualitatively comparable, although they appear significantly thicker and less curved in the 250 nM sample. Additional images of droplets after 24 h incubation are in Supplementary Fig. S10, which includes example images of encapsulated nanotubes produced at 25 nM tile concentration. In this case, nanotube bundling and alignment is not observed as most droplets appear to include only one or two short nanotubes (see also Supplementary Fig. S12).

To gather and compare information about assembly as it occurs in a collection of droplets, we compared skewness and kurtosis of droplet fluorescence microscopy images taken over time, using the image processing protocol described earlier, and Supplementary Note S4.13 (Fig. 3b, d, f). A first observation is that the “steady state” value of kurtosis and skewness is roughly the same in each of these three samples, and thus does not appear significantly affected by the tile concentration. In contrast, the skewness and kurtosis value before the 1 h mark are dependent on concentration, with both skewness and kurtosis increasing more slowly at higher tile concentration. This highlights that skewness and kurtosis are not a measure of the concentration of nanotubes in encapsulation. They, instead, are a measure of the fluorophores (or tiles since each has one fluorophore) incorporated in a structure versus those which are unincorporated. As assembly of nanotubes begins, the ratio of incorporated to unincorporated tiles starts increasing, thus resulting in a rise in skewness and kurtosis. The rate of increase of skewness and kurtosis within 1 h is fastest in the 50 nM sample since the total number of unincorporated tiles itself is low. As there are more unincorporated tiles in the 250 nM sample, the rate of increase of skewness and kurtosis is much slower. For higher tile concentrations, more free tiles contribute to a brighter overall signal for individual droplets (Supplementary Fig. S69).

Next, we examined the effects of macromolecular crowding on encapsulated nanotube assembly. Macromolecular crowding inside living cells influences diffusion thereby changing intracellular reaction rates, and is a major driving force in phase separation. Thus, we included a common crowding agent, polyethylene glycol 8000 (PEG), in our droplets. In a sample containing, 2.5% PEG, assemblies were visible immediately after encapsulation (Fig. 3g). In this case (Fig. 3h), the starting value and rate of increase of skewness and kurtosis in the first 30 minutes are higher than that of the sample without PEG (Fig. 3d), suggesting that the crowding agent assists assembly (Fig. 3h). Additional experiments show that increased concentration of PEG results in an increase in both skewness and kurtosis (Supplementary Fig. S16). However, with concentrations as high as 10% w/v PEG, we observe rapid formation of aggregates rather than programed assemblies (Supplementary Fig. S16c). We hypothesize that at 10% w/v PEG kinetically favored formation of aggregates instead of the intended thermodynamically favored nanotube structures.

Overall, these experiments support the expectation that assembly of encapsulated nanotubes is influenced by tile concentration. In agreement with computational predictions, our droplet images confirm that at higher tile concentration more nanotubes form because nucleation rates are faster (Supplementary Fig. S72). Yet, based on the droplet brightness and the skewness and kurtosis plots, a substantial fraction of non-assembled tiles appears to persist at all concentrations tested on a timescale of hours, in contrast with model predictions, presumably due to unmodeled depolymerization and joining reactions^64^.

### Activating nanotube assembly inside droplets via RNA triggers

**Fig. 4:**
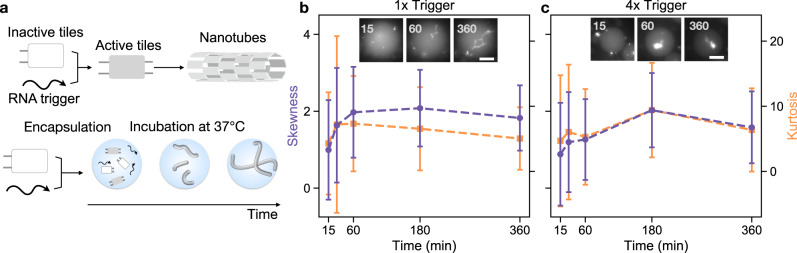
Isothermal assembly of hybrid DNA-RNA nanotubes inside compartments at 37 °C. **a** Inactive DNA tile, lacking sticky-ends necessary for self-assembly on one side of the tile, is activated by the addition of RNA molecules and triggers self-assembly into nanotubes. Inactive tiles and trigger RNA are mixed immediately before encapsulation and incubated at 37 °C. **b**, **c** Representative fluorescence microscopy images of droplets encapsulated with 500 nM inactive tiles, and 1x and 4x RNA trigger (insets) with plots of mean skewness (purple) and kurtosis (orange). Data were presented as mean values ± standard deviation. Data extracted using droplet detection code as described in Supplementary Note S4.13. The number of droplets sampled and a histogram data of radii for sampled droplets in Supplementary Note S5.9 and S5.10. Source data for this figure is provided as a source data file. Scale bars: 20 μm.

To expand the toolkit of NA scaffolds that can be formed inside droplets, we adopted a DNA tile whose assembly into nanotubes is triggered by RNA molecules (Fig. 4a)^36^. In general, assembly of nanotubes cannot occur if one or both of the sticky-end strands is missing from the tile monomer. By excluding one sticky-end strand from the tile annealing mix, it is possible to form inactive tiles that are activatable by the addition of the missing strand, which can be either DNA or RNA. Agarwal et al. previously demonstrated assembly of DNA-RNA hybrid nanotubes within minutes of adding the activating RNA “trigger” strand to pre-annealed inactive DNA tiles at room temperature^36^. RNA can be transcribed as needed from small amounts of templates, a process that can be temporally controlled through transcriptional gene circuits. While it is difficult to produce large assemblies exclusively with RNA, nanostructures made of both DNA and RNA offer a promising route toward transcriptionally-controlled assemblies^65^.

To begin characterizing assembly of DNA-RNA hybrid nanotubes, we added varying concentrations of gel-purified trigger RNA to inactive tiles and subsequently encapsulated the sample. For both 1:1 gel-purified RNA to inactive tile (1x, Fig. 4b) and 4:1 (4x, Fig. 4c) samples, assemblies are visible within 15 minutes. Focusing on the droplets during fluorescence microscopy is non-trivial before nanotubes assemble. When assemblies are present, the skewness and kurtosis values across the sample of droplets measured varies greatly. Fluorescence microscopy images suggest that the assemblies with 4x RNA are disordered aggregates, rather than filaments. We hypothesize that the high concentration of trigger RNA relative to inactive tiles results in undesired hybridization between multiple RNA strands and a single inactive tile, which disrupts the assembly of (defect-less) hybrid nanotubes. These results highlight the limitations of characterizing assembly via skewness and kurtosis, as these measures do not distinguish between predictably assembled nanotubes and disordered aggregates.

### Transcriptional control of nanotube assembly inside droplets

**Fig. 5:**
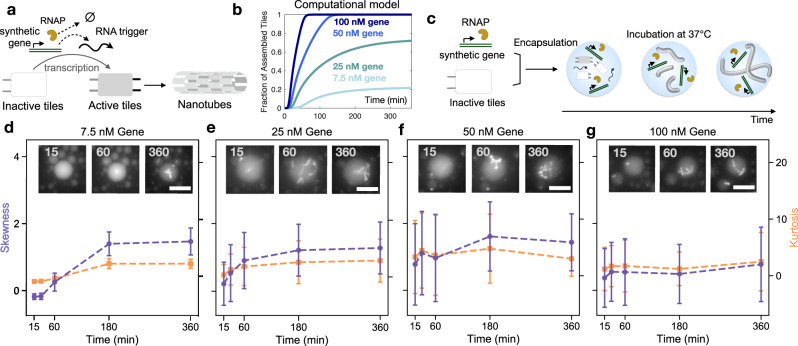
Co-transcriptional isothermal assembly of encapsulated hybrid DNA-RNA nanotubes with in situ trigger transcription at 37 °C. **a** Inactive tiles are activated by an RNA trigger transcribed using a synthetic gene and subsequently assemble into nanotubes. **b** A computational model illustrates that by increasing the concentration of synthetic gene template, we can speed up the assembly kinetics and increase the equilibrium fraction of assembled tiles (modeling details in Supplementary Note S6.2). Increasing amounts of gene template are represented with darker shades of blue. **c** Inactive tiles at 500 nM concentration were encapsulated with transcription mix, 2.5% w/v PEG, and 2.5% v/v RNAP and incubated at 37 °C. **d**–**g** Representative fluorescence microscopy images of droplet samples in which we titrated the amount of synthetic gene transcribing RNA trigger (insets) with plots of mean skewness (purple) and kurtosis (orange) for sampled droplets. The rate of increase of skewness and kurtosis correlates with the concentration of DNA template producing the trigger RNA strand. Data were presented as mean values ± standard deviation. Images were processed using the droplet detection code as described in Supplementary Note S4.13. The number of droplets sampled and a histogram data of radii for sampled droplets are reported in Supplementary Note S5.9 and S5.10. Source data for this figure is provided as a source data file. Scale bars: 20 μm.

In living cells, all molecular components are continuously produced and degraded, including those participating in complex cytoskeletal dynamics. To embed a similar architecture in our system, we use a well-characterized in vitro strategy to produce RNA molecules from linear DNA templates or synthetic genes using bacteriophage T7 RNA polymerase (RNAP) inside droplets^37^. The RNA trigger described in the previous section was transcribed inside droplets in the presence of inactive tiles to operate as an activator that promotes growth of encapsulated nanotubes (Fig. 5a–c)^36^.

First, we investigated how the concentration of template producing trigger RNA affects the temporal evolution of nanotube assembly inside droplets given a fixed concentration of inactive tiles and transcription conditions (buffer mix, NTPs, and RNAP). Simulations using a deterministic ODE model using realistic parameters that capture transcription, RNA-mediated tile activation, and tile assembly (Supplementary Note S6.2) illustrate that both the kinetics and the fraction of assembled tiles become faster with increasing synthetic gene concentration (Fig. 5b). Taking into account loss of activity of RNAP, these simulations also suggest that the equilibrium fraction of assembled tiles can be controlled.

Figure 5d–g shows the corresponding experimental results with representative droplet images and skewness/kurtosis plots of DNA-RNA hybrid nanotubes obtained by in situ RNA production inside droplets. No assembly occurs in the absence of the synthetic gene, as shown in Supplementary Fig. S13. The steady-state values of skewness and kurtosis are reached more rapidly in experiments with higher concentration of genes, presumably due to faster RNA production and tile activation. While this appears to agree with the picture illustrated by the model, several considerations are in order.

First, unlike the two-tile or gel-extracted RNA trigger experiments, nanotubes were not visible via co-transcription without the addition of 2.5% PEG 8000 (Supplementary Fig. S14). Through further assays with varied concentrations of PEG added to the encapsulated co-transcription mix, we determined that a small amount of PEG facilitates visualization of nanotubes during co-transcription of the RNA trigger within droplets. While PEG does not promote aggregation of non-assembling tiles (Supplementary Fig. S15), increased amounts of PEG seems to interfere with the tile assembly process and results in aggregation (Supplementary Fig. S16). PEG is likely facilitating polymerization, promoting nanotube localization near the droplet surface, as well as enhancing the rate of RNA transcription^66^.

For all conditions, there are a number of droplets smaller than 5 µm in radius in which no nanotubes polymerize. This is likely a result of random partitioning of components across the population of droplets, whose effects are more prominent in smaller volumes: the droplets without any polymerized nanotubes may not encapsulate sufficient amounts of nanotube or transcription components (synthetic gene and RNAP) to trigger assembly. For 7.5 nM template, small nanotubes are visible in droplets larger than 15 µm in diameter by 30 minutes. For droplets of all diameters at this template concentration, nanotubes are visible within 60 minutes and continue to grow through 360 minutes. Before nanotubes have polymerized, skewness and kurtosis are near 0 with small variation across sampled droplets. As nanotubes polymerize, the average skewness and kurtosis values increase, as well as their standard deviation. Notably, nanotubes form in droplets at a lower concentration of template than previously reported in bulk solution by Agarwal et al. Although this is likely the result of the presence of the crowding agent PEG, confinement may contribute to lowering the minimum threshold of active tiles for nucleation and assembly of nanotubes, thus reducing the required transcription rate. At high gene concentration (50 and 100 nM), bright spots indicative of nanotube assembly are visible immediately after encapsulation. In smaller droplets (3–7 µm radius), a single nanotube often forms within the first 15 minutes but does not appear to elongate further. In larger droplets (>8 µm radius), branching nanotubes and aggregates are visible within 15 minutes, suggesting that overproduction of RNA promotes formation of incorrect assemblies, as individual tiles may bind to two RNA trigger strands. Consistently higher values of average skewness and kurtosis from the beginning of the experiment confirm that assembly or aggregation occur rapidly after inactive tiles are mixed with transcription components.

### Transient nanotubes formation inside droplets arising from simultaneous RNA transcription and degradation

**Fig. 6:**
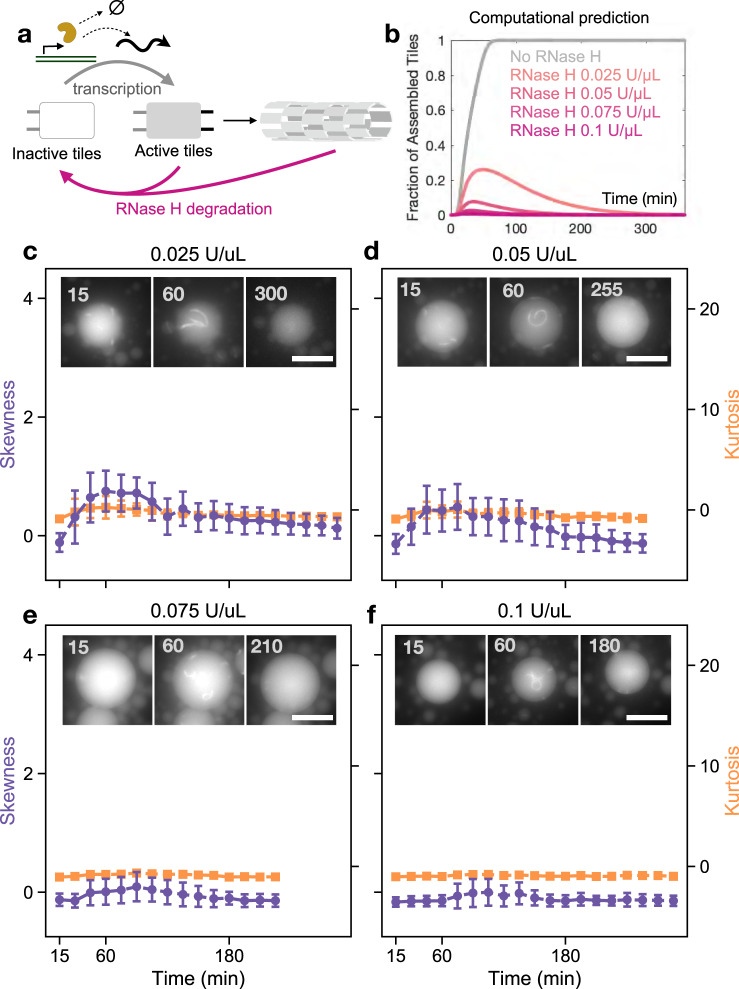
Enzyme-mediated control of assembly and disassembly of encapsulated hybrid DNA-RNA tubes at 37 °C. **a** Schematic of the reactions occurring in a sample that includes inactive tiles, DNA template transcribing the RNA trigger (promoting growth of nanotubes), and RNase H (promoting degradation of nanotubes). **b** Computational prediction showing that loss of activity of RNAP against unchanged RNase H activity yields a temporal pulse in the fraction of assembled tiles, whose peak and duration depend on the amount of RNase H. Increasing amounts of RNase H are represented with darker shades of pink. Modeling details are in Supplementary Note S6.3. **c**–**f** Representative fluorescence microscopy images of the RNase H titration experiments (insets) with plots of mean skewness (purple) and kurtosis (orange) for sampled droplets. Inactive tiles at 500 nM concentration were encapsulated with 100 nM gene template, 2.5% w/v PEG, 2.5% v/v RNAP, and 0.025 U/µL–0.1 U/µL RNase H. These experimental results show that, when RNA trigger transcription and degradation components are simultaneously present, a pulse of nanotube polymerization is observed. Eventually RNase H causes disassembly of the nanotubes at a speed that depends on the RNase H concentration, presumably due to a decrease of RNAP activity. Skewness and kurtosis are plotted in shades of purple and orange, respectively. Data were presented as mean values ±  standard deviation. Data extracted using droplet detection code as described in Supplementary Note S4.13. The number of droplets sampled and histograms of radii for sampled droplets are in Supplementary Notes S5.9 and S5.10. (Also see: Supplementary Movie 4). Source data for this figure is provided as a source data file. Scale bars: 20 μm.

Next, we sought to introduce nanotube disassembly using RNase H, an enzyme that hydrolyzes RNA in DNA-RNA complexes (Fig. 6a). RNase H has been widely used to control degradation in a variety of artificial in vitro transcriptional circuits as well as within nanotube systems^35^. We previously showed that RNase H can deactivate free (unpolymerized) tiles by degrading the portion of RNA trigger bound to DNA promoting nanotube disassembly^35,36^. When both RNAP and RNase H are present with inactive tiles and transcription reagents in nonencapsulated solutions, a transient pulse of nanotube assembly was observed^36^. This pulse may be attributed to a progressive loss of activity of RNAP relative to the initial transcription “burst”, and to accumulation of incomplete RNase H degradation products^67–69^. Figure 6b illustrates the predictions of our unfitted computational model capturing transcription (including loss of RNAP activity), tile activation and assembly, as well as RNase H degradation: the model qualitatively predicts a pulse in fraction of assembled tiles (Supplementary Note S6.3), and the height and duration of this pulse depend on the amount of RNase H present. Thus, we expect to see this encapsulated system to yield transient nanotube assembly, with dynamics that can be tuned by changing the amount of RNase H.

We observed transient nanotube formation in droplets for different concentrations of RNase H, while the inactive tile concentration was kept at 500 nM, template at 100 nM, and 2.5% v/v of T7 RNAP (Fig. 6c–f and Supplementary Movie 4). In the absence of RNase H, droplets with synthetic gene at 50 and 100 nM showed nanotube aggregation likely due to overproduction of RNA trigger (Fig. 5f, g). In the presence of RNase H such aggregation was not visible. The skewness and kurtosis plots exhibit a transient pulse and a small standard deviation when compared to experiments that do not include both transcription and degradation, presumably due to most tiles remaining unassembled throughout the experiment. At lower amounts of RNase H, 0.025 U/µL and 0.05 U/µL, nanotubes are visible until about 3 h after encapsulation (Fig. 6c, d). At higher amounts of RNase H, assembly is delayed and fewer nanotubes are visible, confirming that it is possible to tune the temporal response of assembly and the nanotube density inside the compartments (Fig. 6e, f). At 0.05 U/µL RNase H and higher, many nanotubes appear to curl into loops in most droplets. It is unclear what is causing this morphology, but it could be a result of both crowding and degradation of RNA. As the density and dynamics of nanotubes inside droplets depends on transcription and degradation rates, both characteristics may be controlled by changing either the relative concentration of enzymes or synthetic gene for RNA production.

These experiments indicate that more complex genetic programs relying on RNA production and degradation may be encapsulated to control nanotube assembly^35^. A notable challenge toward sustained transcription and degradation dynamics is the accumulation of abortive and elongated transcripts, which can cause side reactions and crosstalk^70^. These undesired transcripts may bind to inactive tiles producing “waste” complexes that can no longer be activated by the correct RNA trigger^36^. Additional RNases may be included to reduce the influence of “waste” products in the system^71^.

## Discussion

We demonstrated the construction of artificial dynamic scaffolding systems for minimal cell mimics through the assembly of NA tubular nanostructures inside water-in-oil droplets. When compared to cytoskeletal scaffolds in biological cells, NA nanotubes offer similar structural features with a minimal number of components required for assembly and regulation^22,35^. We established that nanotube assemblies can be obtained with four different tile (monomer) designs, a toolkit that can be easily expanded. Monomers can be annealed inside droplets or pre-annealed and subsequently encapsulated. We showed that distinct, noninteracting tile types can be encapsulated and assembled simultaneously, making it possible to build distinct scaffolds. Conversely, we verified that encapsulated tiles and NA strands designed to interact generate the desired products, which is consistent with the outcome of nonencapsulated reactions. Finally, nanotube assembly was modularly integrated with RNA transcription and degradation processes, whose competition enabled the dynamic, autonomous control of self-assembly inside droplets^36^. It is remarkable that the encapsulation methods described here make it possible to operate NA structures and circuits with marginal deviation from their nonencapsulated behavior.

We have characterized different methods for scaffold encapsulation, focusing on an emulsion method that generates stable droplets with a broad range of sizes and compositional diversity. These droplets are ideal for long-term observation of nanotube assembly, however they appear inadequate to explore the capacity of nanotubes to induce compartment deformation. Control of compartment deformation has been demonstrated using cytoskeletal polymers, by tuning compartment rigidity and size, and the stiffness of the polymers, in agreement with computational predictions^72–74^. Similar shape control could be achieved with DNA filament systems, where stiffness may be changed by tuning nanotube design or by forming nanotube networks^75^. Nanotubes may be encapsulated in liposomal and phase separated (membrane less) synthetic cells that have been used to compartmentalize various proteins and synthetic circuits^11,76–79^. However, preliminary investigations revealed many challenges in obtaining high yield encapsulation of DNA structures in cell-sized vesicles. These challenges include the selection of lipids that generate stable vesicles with minimal surface interaction with DNA nanostructures, and of methods which produce a high yield of vesicles with controllable size while avoiding damage to the nanostructures^40,80,81^. A promising route is given by microfluidic approaches that make it possible to build cell-sized vesicles with control over their membrane and internal composition^82,83^.

To track the growth of NA scaffolds in time, we complemented qualitative observations with a quantitative method that tracks statistical properties of droplet epifluorescence microscopy images. We showed that skewness and kurtosis (third and fourth moment) of intensity histograms provide high-throughput information about the extent of monomer condensation. While this statistical analysis alone does not allow for distinguishing correct and incorrect assemblies, it makes it possible to compare tile condensation in a large population of droplets under different concentrations of monomers, crowding agents, synthetic genes, as well as enzymes. We found significant diversity of condensation extent across droplets, with smaller droplets presenting the highest variability, which is likely the result of several phenomena including partitioning noise, assembly errors, and surface interactions that may promote uneven bundling of nanotubes. Such variability increases when assembly is triggered by RNA molecules that may generate more incorrect assemblies and undesired aggregates. However, the simultaneous presence of enzymes producing and degrading RNA appears to reduce variability, as quantified by our statistical measures, a counterintuitive result given that additional interacting species are expected to amplify the effects of partitioning noise^37^. It is likely that a lower standard deviation of both skewness and kurtosis is simply due to the fact that most tiles remain unpolymerized in the presence of RNase H.

Our study points to the potential application of NA nanostructures as a rich toolkit for generating complex scaffolding components in artificial cells. Our demonstration could be immediately expanded to include multiple filamentous scaffolds to spatially organize distinct organic or inorganic ligands, and each scaffold may be individually controlled by distinct synthetic genes or circuits^84^. As chemically modified nanotubes can be made resilient to cytoplasmic conditions, they could be controlled through a multitude of circuit parts involving transcription-translation^85,86^. While we focused on assembly of one class of filamentous structures from tiles, similar tile variants can be used to build fibers and two dimensional assemblies, and RNA variants may be produced co-transcriptionally^24,87^. While our work falls short of seeking to mimic active cytoskeletal behaviors, these nanotubes may be used as tracks for molecular motors that could perform work and transfer cargo within compartments^22,88,89^. Further, NA nanotubes could be organized in higher-order structures using other types of assemblies such as DNA origami^90^. Finally, because NA scaffold assembly can be regulated by complex synthetic gene networks and strand displacement reactions within compartments, the coordination of multi-compartment behavior through diffusing molecules could open up exciting opportunities to build artificial tissues with programmable development and patterning^40,91^.

## Methods

### DNA oligonucleotides and enzymes

Oligonucleotides were purchased from IDT DNA. T7 RNAP was purchased from Lucigen^®^ and RNase H was purchased from Promega^™^. Transcription reagents were purchased from Lucigen^®^ and New England Biolabs. Oligonucleotide sequences and modifications, as well as further details on the preparation of DNA nanotubes and transcription reactions, are available in the Supplementary Information (SI).

### Generation of microemulsion droplets

Two alternative methods for the generation of the microdroplets were employed. The first method, or “shaken method”, consisted in simply vortexing a emulsion of oil-surfactant mixture and aqueous buffer solution that contained the reagents (Supplementary Note S4.7). By adjusting mixing time and vortexing speed appropriately, we generated populations of droplets with a broad size distribution, with radii ranging from ∼1 µm to >20 µm. About 100 µl of droplet emulsion was generated by mixing 20 µl reaction mix with 80 µl of oil-surfactant mix (FC-40 Fluorinert^™^ oil (86508-42-1, Sigma-Aldrich) that contained 2% (w/v) perfluoropolyether-polyethylene glycol (PFPE-PEG) block-copolymer fluorosurfactant with Krytox-FSH via an amide group (Ran Biotechnologies)) in DNA LoBind tubes (Eppendorf) using a vortex mixer for 60 s. The second method consisted in using microfluidic chips to generate emulsions (Supplementary Note S4.8). This allowed us to produce large numbers of droplets with a much narrower size distribution (Fig. 1d) but it was technically challenging to produce very small droplets with diameters smaller than 20 µm. For these reasons, we adopted the shaken method for all experiments reported in this paper, except for those reported in Fig. 1d.

### Fluorescence time lapse experiments

Droplet samples were imaged using an inverted microscope (Nikon Eclipse TI-E) with Nikon Plan Fluor 20X/0.5 NA objectives. Droplets were placed in Ibidi chamber slides (µ-Slide VI ^0.4^, hydrophobic coating) with the inputs to the channels sealed with vacuum grease (Dow Corning^®^) and VWR micro slides to prevent evaporation throughout experiments. Droplets encapsulating transcription reactions were incubated in the Ibidi chambers on a ThermoPlate (Nikon Inc, Tokai Hit) set to 37 °C during imaging.

### Confocal fluorescence microscopy

Droplet samples were imaged in an Ibidi chamber slide (µ-Slide VI ^0.1^, hydrophobic coating) on a Leica TCS SP8-STED confocal microscope with 63x/1.20 NA water-immersion objective

### Image analysis and data processing

Droplets were monitored for up to 24 h and images were processed using an in-house Python scripts to identify droplets and extract pixel brightness values from fluorescence images. The code is available through a Github repository (Supplementary Note S4.13), but is also available upon request from the authors. To measure the skewness and kurtosis for the distribution of pixel brightness values extracted for each droplet, first a list of unique pixel brightness values was calculated for each droplet based on the number of pixels in each bin and the bin width of the histogram for each droplet. Then skewness and kurtosis are calculated for the unique pixel brightness values list using the skew and kurtosis functions in the Python Pandas library. The average skewness and kurtosis values, along with the standard deviation for each measurement, was calculated for all droplets measured at each time point of each experiment. Standard deviation was chosen over the variance or other measurements as it represents the spread of the values across the measure samples. Further details for the same are available in the SI.

### Statistics and reproducibility

Data which is quantified in the manuscript comes from single experiments containing hundreds of droplets. Each experimental condition, however, was reproduced at least twice with some assays repeated three or more times for gathering of confocal data, control experiments, and optimizing imaging protocols.

### Reporting Summary

Further information on research design is available in the Nature Research Reporting Summary linked to this article.

## Supplementary information

Supplementary Information Notes

Supplementary Movie 1

Supplementary Movie 2

Supplementary Movie 3

Supplementary Movie 4

Reporting Summary

Description of Additional Supplementary Files

## Data Availability

The data for the current study are available from the corresponding author on reasonable request. Source data for Figs. 2 to 6 are provided with the paper.

## Source data

Source Data

## Code availability

Both the custom Python image processing code and the ODE models developed in MATLAB are available on Github here: https://github.com/klockemel/dropletDetection_dynamicSelfAssemblyProject. 10.5281/zenodo.4763253
