# Supplementary material for: Dynamic self-assembly of compartmentalized DNA nanotubes
Source: Nat Commun. 2021 Jun 11;12:3557. doi: 10.1038/s41467-021-23850-1 (PMC8196065; doi:10.1038/s41467-021-23850-1)
Supplement: Supplementary file 1 — Supplementary Information Notes [file 41467_2021_23850_MOESM1_ESM.pdf]

Supplementary Information: Dynamic Self-assembly of Compartmentalized DNA Nanostructures

Siddharth Agarwal[1,+], Melissa A. Klocke[2,+], Passa E. Pungchai[1], Elisa Franco[3]

[1]Department of Bioengineering, University of California, Los Angeles, CA, USA
[2]Department of Mechanical Engineering, University of California, Riverside, CA, USA
[3]Department of Mechanical Engineering, University of California, Los Angeles, CA, USA

+ these authors contributed equally,
* corresponding author (efranco@seas.ucla.edu)

Email list
sidagarwal@g.ucla.edu, klocke@ucr.edu, passaep@g.ucla.edu, efranco@seas.ucla.edu

Table of Contents

SUPPLEMENTARY NOTE 1 SEQUENCES **4**

SUPPLEMENTARY NOTE 2 SYNTHETIC GENES FOR PRODUCTION OF RNA STRANDS **9**

SUPPLEMENTARY NOTE 3 REAGENTS **10**

S3.1 OLIGONUCLEOTIDES 10
S3.2 ENZYMES 10
S3.3 BUFFERS, DYES, AND OTHER REAGENTS 10
S3.4 OIL AND SURFACTANT 10

SUPPLEMENTARY NOTE 4 METHODS **12**

S4.1 SINGLE-TILE DNA NANOTUBE PREPARATION: ANNEAL PRIOR TO ENCAPSULATE 12
S4.2 SINGLE-TILE DNA NANOTUBE PREPARATION: ENCAPSULATE PRIOR TO ANNEAL 12
S4.3 TWO-TILE DNA NANOTUBE PREPARATION 12
S4.4 DNA INACTIVE TILE PREPARATION 12
S4.5 ACTIVATION OF TILE ASSEMBLY BY ADDING STRAND S4 13
S4.6 ACTIVATION OF TILE ASSEMBLY BY CO-TRANSCRIPTION OF THE TRIGGER STRAND 13
S4.7 SHAKEN METHOD FOR WATER-IN-OIL DROPLET FORMATION 13
S4.8 MICROFLUIDIC METHOD FOR WATER-IN-OIL DROPLET FORMATION 14
S4.9 EPIFLUORESCENCE MICROSCOPY 14
S4.10 CONFOCAL FLUORESCENCE MICROSCOPY 15
S4.12 RNA EXTRACTION 16
S4.13 DROPLET DETECTION AND PIXEL BRIGHTNESS VALUE EXTRACTION VIA PYTHON 17

SUPPLEMENTARY NOTE 5 ADDITIONAL DATA AND ANALYSIS **24**

S5.1 POSITIONING OF DROPLETS INSIDE IBIDI CHAMBER (μ-SLIDE VI $^{0.4}$) 24
S5.2 MORPHOLOGY OF MATURE TWO-TILE NANOTUBES AFTER 24 HOURS OF INCUBATION 25
S5.3 CONFOCAL MICROSCOPY IMAGES 26
S5.4 TWO-TILE NANOTUBE SYSTEM AT 25 NM CONCENTRATION 27
S5.5 DNA-RNA HYBRID TILES WITHOUT GENE TEMPLATE 28
S5.6 CO-TRANSCRIPTIONAL ASSEMBLY WITHOUT CROWDING AGENT 28
S5.7 EFFECTS OF CROWDING AGENT ON TWO-TILE NANOTUBE SYSTEM 29
S5.7 INFLUENCE OF PEG ON TRANSCRIPTIONALLY ACTIVATED NANOTUBES 30
S5.8 INFLUENCE OF EXPOSURE TIME ON SKEWNESS AND KURTOSIS 31
S5.9 NUMBER OF DROPLETS DETECTED IN EACH EXPERIMENTAL STUDY 34
S5.10 RADII OF THE DETECTED DROPLETS IN EACH EXPERIMENTAL STUDY 39
S5.11 DROPLET RADIUS VERSUS SKEWNESS AND KURTOSIS 54
S5.12 TWO-TILE NANOTUBES IN BULK 82
S5.13 TWO-TILE NANOTUBES IN DROPLETS FOR EXTENDED OBSERVATIONS 83
S5.14 AUTOFLUORESCENCE OF WATER-IN-OIL DROPLETS WITHOUT FLUORESCENTLY LABELED STRANDS 84
S5.15 INCREASED BACKGROUND FLUORESCENCE FOR INCREASING TILE CONCENTRATION IN TWO-TILE DESIGN EXPERIMENTS 85
S5.16 LENGTH MEASUREMENT OF NANOTUBES USING TWO-DIMENSIONAL CONTOUR PROJECTION 86
S5.17 EFFECT OF DNASE I ON TWO-TILE DNA NANOTUBES 87

SUPPLEMENTARY NOTE 6 COMPUTATIONAL MODELING **88**

S6.1 TILE ASSEMBLY MODELS 88

S6.2 Model capturing transcription of RNA and RNA-mediated tile activation and assembly 91
S6.3 Model capturing transcription and degradation of RNA and RNA-mediated tile activation and assembly 93

REFERENCES **97**

Supplementary Note 1 Sequences

PAGE purified DNA sequences were ordered from IDT DNA (Coralville, IA, USA). Sequences of strands composing the tiles are taken from Rothemund et al., 2004[1] and Agarwal & Franco, 2019[2].

In addition to reporting DNA sequences, this section also includes schematics representing each tile type. Red circles on the blue strand represent the location Cy3 or ATTO647N fluorophores as stated in the strand sequence.

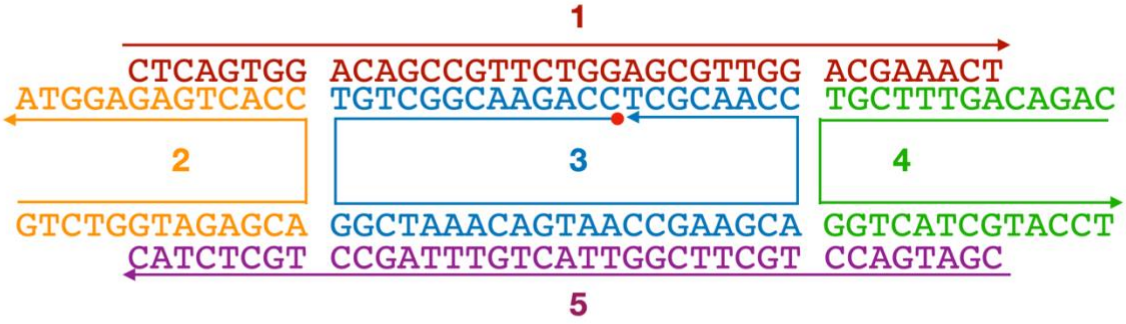

Figure S1: Tile designed for assembly of single-tile nanotubes, design variant 1.

Strand position	Name	Sequence
1	5bSE1	5'- CTC AGT GGA CAG CCG TTC TGG AGC GTT GGA CGA AAC T
2	5bSE2	5'- GTC TGG TAG AGC ACC ACT GAG AGG TA
2T	5bSE2 T	5'-G TCT GGT AGA GCA CCA CTG AGA GGT A
3	5bSE3	5'- cy3-CCA GAA CGG CTG TGG CTA AAC AGT AAC CGA AGC ACC AAC GCT
4	5bSE4	5'- CAG ACA GTT TCG TGG TCA TCG TAC CT
5	5bSE5	5'- CGA TGA CCT GCT TCG GTT ACT GTT TAG CCT GCT CTA C

Table S1: DNA sequences for single-tile nanotubes. Nomenclature of the sequences in the table reflects labeling of strands in the lab and the original name of the strands in the paper by Rothemund et al., 2004[1]. The '5bSE2 T' strand was used during encapsulation of nanotubes by microfluidics (Fig 1d, SI section S4.8) and includes a short single stranded toehold that does not affect the assembly pathway.

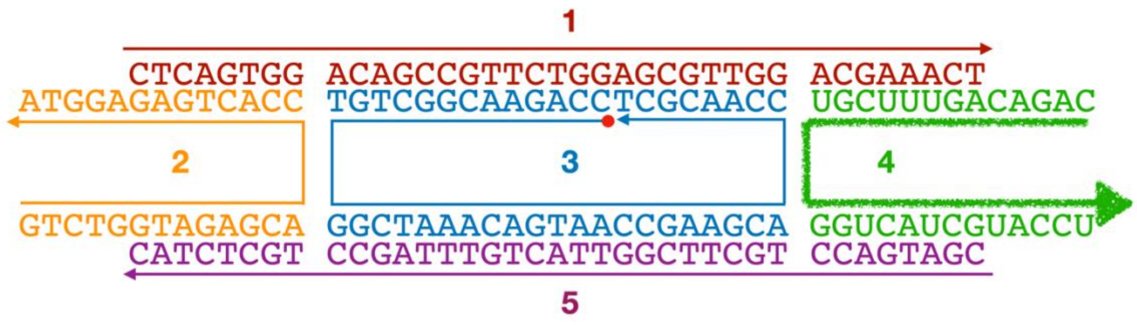

Figure S2: DNA-RNA hybrid tile. Strand 4 is an RNA molecule.

**Strand position**	**Name**	**Sequence**
1	5bSE1	5'- CTC AGT GGA CAG CCG TTC TGG AGC GTT GGA CGA AAC T
2	5bSE2	5'- GTC TGG TAG AGC ACC ACT GAG AGG TA
3	5bSE3	5'- cy3-CCA GAA CGG CTG TGG CTA AAC AGT AAC CGA AGC ACC AAC GCT
**4**	**RNA-5bSE4**	**5'- CAG ACA GUU UCG UGG UCA UCG UAC CU**
5	5bSE5	5'- CGA TGA CCT GCT TCG GTT ACT GTT TAG CCT GCT CTA C

Table S2: DNA sequences for the DNA-RNA hybrid nanotubes. Bold indicates RNA strand.

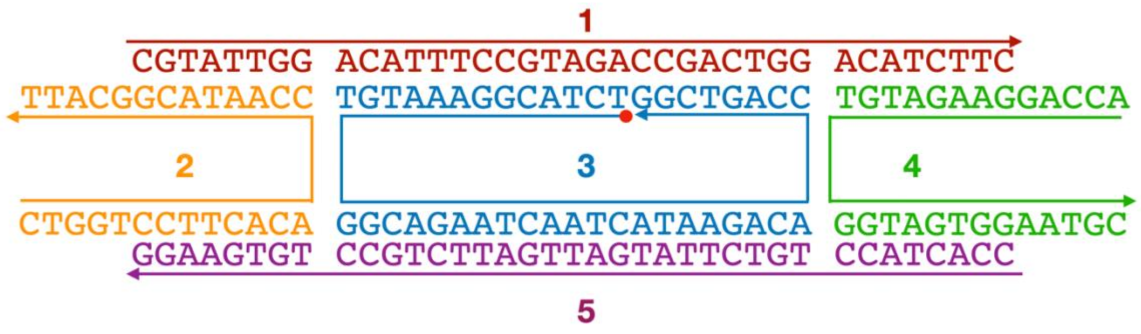

Figure S3: Tile designed for single-tile nanotube assembly, design variant 2.

Strand position	Name	Sequence
1	5bRE1	5'- CGT ATT GGA CAT TTC CGT AGA CCG ACT GGA CAT CTT C
2	5bRE2	5'- CTG GTC CTT CAC ACC AAT ACG GCA TT
3	5bRE3	5'- atto647N-TCT ACG GAA ATG TGG CAG AAT CAA TCA TAA GAC ACC AGT CGG
4	5bRE4	5'- ACC AGG AAG ATG TGG TAG TGG AAT GC
5	5bRE5	5'- CCA CTA CCT GTC TTA TGA TTG ATT CTG CCT GTG AAG G

Table S3: DNA sequences for single tile system, design variant 2. Nomenclature of the sequences in the table reflects labeling of strands in the lab and the original name of this tile variant in the paper by Rothemund et al., 2004[3].

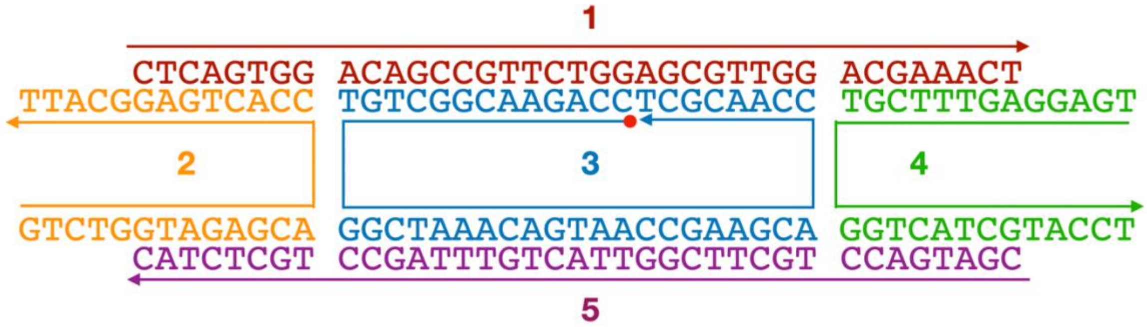

Figure S4: Tile A - Tiles designed for two-tile nanotube assembly.

**Strand position**	**Name**	**Sequence**
1	5bSEp1	5’- CTC AGT GGA CAG CCG TTC TGG AGC GTT GGA CGA AAC T
2	5bSEp2	5’- GTC TGG TAG AGC ACC ACT GAG GCA TT
3	5bSEp3	5’- cy3-CCA GAA CGG CTG TGG CTA AAC AGT AAC CGA AGC ACC AAC GCT
4	5bSEp4	5’- TGA GGA GTT TCG TGG TCA TCG TAC CT
5	5bSEp5	5’- CGA TGA CCT GCT TCG GTT ACT GTT TAG CCT GCT CTA C

Table S4: DNA sequences of Tile A for nanotube assembly from two tiles. The strand nomenclature reflects the one adopted by Rothemund et al., 2004[1] to describe the two-tile nanotubes in which tiles form a perpendicular, alternated “ring” pattern.

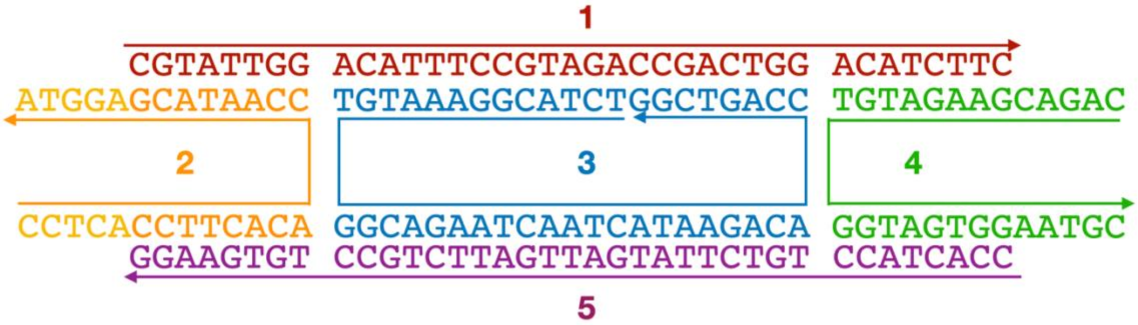

Figure S5: Tile B - Tiles designed for two-tile nanotube assembly.

**Strand position**	**Name**	**Sequence**
1	5bREp1	5'- CGT ATT GGA CAT TTC CGT AGA CCG ACT GGA CAT CTT C
2	5bREp2	5'- CCT CAC CTT CAC ACC AAT ACG AGG TA
3	5bREp3	5'- TCT ACG GAA ATG TGG CAG AAT CAA TCA TAA GAC ACC AGT CGG
4	5bREp4	5'- CAG ACG AAG ATG TGG TAG TGG AAT GC
5	5bREp5	5'- CCA CTA CCT GTC TTA TGA TTG ATT CTG CCT GTG AAG G

Table S5: DNA sequences of Tile B for nanotube assembly from two tiles. The strand nomenclature reflects the one adopted by Rothemund et al., 2004[1] to describe the two-tile nanotubes in which tiles form a perpendicular, alternated "ring" pattern.

Supplementary Note 2 Synthetic genes for production of RNA strands

Templates (genelets) include 4 base “sealing” domains in genes at the 5’ end of non-template strand, to prevent breathing at the promoter site. Each RNA strand was designed to start with ‘G’ on the 3’ end, to ensure good transcription yield[2,4].

NonTemplate	5’- GCG CTA ATA CGA CTC ACT ATA GCA GAC AGT TTC GTG GTC ATC GTA CCT
Template	5’- AGG TAC GAT GAC CAC GAA ACT GTC TGC TAT AGT GAG TCG TAT TAG CGC

Table S6: DNA sequences of the synthetic gene designed to produce RNA trigger (5BSE4).

Supplementary Note 3 Reagents

S3.1 Oligonucleotides

Lyophilized, PAGE-purified DNA oligonucleotides were purchased from Integrated DNA Technologies (Coralville, IA). All strands were resuspended in nuclease free water (Thermo Fisher Cat. no. AM9932), quantitated by UV absorbance at 260 nm using a Thermo Scientific Nanodrop 2000c Spectrophotometer, and stored at -20°C. RNA strands were transcribed, and gel-extracted in house according to the protocol in SI Section S4.12.

S3.2 Enzymes

T7 RNA Polymerase was purchased from Lucigen® as part of the AmpliScribe™ T7-Flash™ Transcription Kit (Cat. No. ASF3507). RNase H was purchased from Promega™ (Ref. No. M4281, 2.1 units/µL as reported by the manufacturer). Prior to use, RNase H was diluted down to 0.25X concentration in a solution containing 1 mM DTT, 50 mM KCL and 50%v/v glycerol. Enzymes were stored at -20°C. DNase I was purchased from Lucigen® as part of the AmpliScribe™ T7-Flash™ Transcription Kit (Cat. No. ASF3507).

S3.3 Buffers, dyes, and other reagents

Transcription buffer was taken from the Lucigen® AmpliScribe™ T7-Flash™ Transcription Kit (Cat. No. ASF3507). Nucleotide Triphosphates (NTPs) were purchased from New England Biolabs (NEB, Cat. No. N0450S). Tris-acetate-EDTA (TAE, Cat. no. 15558026) and Tris-borate-EDTA (TBE, Cat. no.LC6675) buffers were purchased from Thermo Scientific. SYBR™ Gold Nucleic Acid Gel Stain was purchased from Invitrogen™, Thermo Fisher Scientific (Cat. no. S11494). Polyethylene Glycol 8000 (PEG), was purchased from Thermo Fisher Scientific (Cat. no. BP233-100).

S3.4 Oil and surfactant

We used Fluorinert™ FC-40, an immiscible fluorocarbon oil, for our water-in-oil droplets. Fluorinert™ FC-40 was purchased from Sigma-Aldrich™ (CAS Number 86508-42-1 MDL number MFCD00131095). We used RAN Biotech's non-ionic surfactant for droplet encapsulation (cat#: 008-FluoroSurfactant-); which is a linear poly(ethylene glycol), MW ca. 600, coupled on each end to Krytox-FSH via an amide group.

The oil-surfactant mix includes FC-40 fluorinated oil and 2% w/v perfluoropolyether-polyethylene glycol (PFPE-PEG) block-copolymer fluorosurfactant with Krytox-FSH via an amide group (Ran Biotechnologies).

Supplementary Note 4 Methods

S4.1 Single-tile DNA nanotube preparation: anneal prior to encapsulate

After annealing, DNA nanotubes were diluted to target concentration for encapsulation in 1x TAE buffer with 12.5 mM $MgCL_2$. For encapsulation with the shaken protocol, 20 µL of 5b SEs nanotubes at 500 nM dilution were pipetted into oil-surfactant mixture and vortexed for 60 seconds following the shaken droplet protocol in section S4.7 (see experiment in Fig. 1c). For encapsulation with the microfluidic chip, nanotubes were diluted to 350 nM in 1x TAE buffer with 12.5 mM $MgCL_2$ before encapsulation using the microfluidic protocol described in section S4.8 (see experiment in Fig. 1d).

S4.2 Single-tile DNA nanotube preparation: encapsulate prior to anneal

All the strands for tile variants 1 (Fig. S1) and 2 (Fig. S3) that form single-tile nanotubes were mixed for a target 250 nM tile concentration (each tile), by adding 250 nM (target concentration) of each DNA strand 1, 2, 3-Cy3/3-Atto647N, 4 and 5 to 1x TAE buffer with 12.5 mM $MgCL_2$ and nanopure water inside DNA Lo-bind tubes. After vortexing briefly, and encapsulation following the shaken droplet protocol described in section S4.7, the solution was annealed using an Eppendorf Mastercycler Gradient thermal cycler by heating to 90°C, and cooling to 25°C, over a 6-hour period. Tile variant 1 was encapsulated alone (see Fig. 1f), or simultaneously encapsulated with variant 2 (see Fig. 1g)

S4.3 Two-tile DNA nanotube preparation

Tile A and tile B were annealed separately at a target 2 µM concentration by adding 2 µM (target concentration) of DNA strands 1, 2, 3-Cy3, 4 and 5 for each tile to 1x TAE buffer with 12.5 mM $MgCL_2$ and nanopure water inside two different DNA Lo-bind tubes. After vortexing briefly, each sample was annealed using an Eppendorf Mastercycler Gradient thermal cycler by heating to 90°C, and cooling to 25°C, over a 6-hour period.

For encapsulation, tile A and tile B were mixed into the same DNA Lo-bind tube at target concentrations and encapsulated via the shaken droplet protocol in section S4.7.

S4.4 DNA inactive tile preparation

Inactive tile solution was prepared to target 1µM tile concentration by adding 1 µM (target concentration) of DNA strands 1, 2, 3-Cy3/3-Atto647N, and 5 to 1x TXN buffer (RNA Pol Reaction Buffer from Lucigen®) and nanopure water inside

DNA Lo-bind tubes. After vortexing for 60 seconds, the solution was annealed using an Eppendorf Mastercycler Gradient thermal cycler by heating to 90°C, and cooling to 25°C, over a 6-hour period.

S4.5 Activation of tile assembly by adding strand S4

Annealed inactive tiles were incubated in the Mastercycler at 37°C prior to adding the missing strand 4. RNA strand 4 was added (target strand concentration to match the tile concentration) prior to encapsulating. After encapsulation, droplets were incubated at 37°C and monitored via fluorescence microscopy.

S4.6 Activation of tile assembly by co-transcription of the trigger strand

Annealed inactive tiles were incubated at 37°C prior to addition of the transcription mix and synthetic gene. The transcription mix includes RNAP (2.5% v/v RNAP (AmpliScribe™ T7-Flash™ Transcription Kit, Cat. No. ASF3507)) and 1x transcription buffer (AmpliScribe™ T7-Flash™ Transcription Kit, Cat. No. ASF3507), 4 mM each nucleoside triphosphates (NTPs) 2.5% w/v PEG 8000 and 10 mM $MgCL_2$. Unless otherwise noted, we used 100 nM annealed synthetic template for transcribing missing RNA strand 4 for an inactive tile concentration of 0.5 µM. Inactive tiles, transcription mix, and synthetic gene were mixed, rapidly encapsulated, and droplets were incubated 37°C as described in section S4.7. Droplets were imaged using a fluorescence microscope for several hours as described in section S4.9.

S4.7 Shaken method for water-in-oil droplet formation

The procedure described here is for the formation of 100 µl droplet emulsion. We pipette 80 µl oil-surfactant mix into a microtube. We then add to it 20 µl of the oligonucleotide containing aqueous phase. Emulsion droplets are formed by vortexing for 60s on a benchtop vortexer. Successful emulsification is indicated by a milky appearance of the sample (the aqueous phase will be dispersed throughout the oil phase at this point). We note that the average droplet size is affected by both time and vortexing speed, thus consistency of this step is essential for repeatable results. Pipetting the sample for imaging directly after emulsification yields too dense a sample of droplets which will result in stacked, multilayers of droplets within the imaging. If droplet overlap is excessive, data from individual droplets cannot be gathered and processed. In addition, overlap inhibits code-based droplet detection (Section S4.13). In that case image processing/droplet identification must be done manually. To get a monolayer of droplets, the emulsion should be allowed to set for 30-60 seconds before pipetting 50-70 µl of the emulsion from below the dense droplet layer at the top of the sample into the well of an Ibidi chamber slide (µ-Slide VI $^{0.4}$ - hydrophobic coating). To

prevent contamination and evaporation of the sample, we seal the well by covering the top edge of each input with vacuum grease (Dow Corning®) and cover with a glass coverslip. The assembly process begins as soon as the encapsulation step is completed so imaging via fluorescence microscopy is started directly after the sample is loaded into the imaging chamber as described in section S4.9.

For confocal microscopy, the sample is mixed and allowed to set for at least 2 hours prior to imaging. Nanotube motion during confocal imaging will result in poorly focused and blurry images.

S4.8 Microfluidic method for water-in-oil droplet formation

Microfluidic droplets were produced using the Dolomite 3D flow focusing 100 µm chip (hydrophobic coating, #3200434) and associated connectors (Linear Connector 4-way # 3000024 x2, Chip Interface H #3000155). Before preparing the droplet samples, the syringe pumps and microfluidic devices were assembled and the microscope was brought into focus on the junction of the chip sitting in the Chip Interface H. Next, 3 input, 1 output, and 4 extra pieces of tubing (IDEX Health and Science, FEP 1/16x.020x100ft #1548XL) were cut; input and output tubing were cut long enough to reach from syringe pumps to the microscope stage, while the 4 extra pieces of tubing were only 1 inch long each. Tubing was threaded through the linear connectors: 3 inputs and 1 extra piece for one connector, and 1 output and three extra pieces for the other connector. The tips of the tubing should either even or slightly drawn into the gasket of the linear connector. Extra 1inch pieces of tubing are present only to hold the shape of the gasket for a good seal with the chip. Nanotube samples were prepared using single tile nanotube variant 1 with strands 5bSE1, 5bSE2 T, 5bSE3, 5bSE4 and 5bSE5 as described in section S4.1. 200 µL 350 nM 5-base Toehold nanotubes was loaded into a syringe (BD 1 mL Syringe Luer-Lok™ Tip with 25g x ⅝ BD PrecisionGlide™ Needle secured with Parafilm), and two syringes were loaded with 300 µL of oil-surfactant mixture each. After threading input tubings with the appropriate syringe needles, tubing was primed by pumping at a rate of 0.2 mL/min via syringe pumps (SyringePump.com #NE-4000, #NE-300). When fluid was visible at the linear connectors, priming was stopped, and the microfluidic chip was loaded into the Chip Interface H and linear connectors attached. Droplets were made by flowing the aqueous sample at 2,000 µL/h and oil phase at 3,000 µL/h, after first flowing the aqueous sample phase only. Droplets were collected in a DNA Lo-bind tube before being loaded into an Ibidi imaging chamber (µ-Slide VI $^{0.4}$, hydrophobic coating) for imaging via fluorescence microscopy.

S4.9 Epifluorescence microscopy

All fluorescent samples inside droplets were imaged using an inverted microscope (Nikon Eclipse TI-E) with Nikon Plan Fluor 20X/0.5 NA objective. All unencapsulated samples were imaged using a 60X/1.4 NA oil immersion objective.

Control experiments of non-encapsulated nanotubes were imaged at 50 nM tile concentration in corresponding experimental buffer conditions (either 1x TAE and 12.5 mM $MgCL_2$, or 1x transcription buffer); samples were placed on a Fisherbrand microscope glass slide (12-545E No. 1, thickness=0.13 to 0.17 mm; size: 50 x 22 mm) and gently covered with VWR Micro Slides (Plain, Selected, Pre-cleaned, 25 x 75 mm, 1.0 mm thick) or Fisherbrand™ Cover Glasses (cat. No. 12-545F). Droplet-encapsulated nanotubes were imaged in an Ibidi chamber (µ-Slide VI $^{0.4}$, µ-Slide VI $^{0.1}$, hydrophobic coating) with the inputs to the channels sealed with vacuum grease (Dow Corning®) and VWR micro slide or Fisherbrand™ Cover Glasses (cat. No. 12-545F) to prevent evaporation. Droplets encapsulating transcription reactions were incubated in the Ibidi chambers on a ThermoPlate (Nikon Inc, Tokai Hit) set to 37°C during imaging.

Encapsulated nanotubes labeled with Cy3 fluorescent molecules were imaged using the Cy3 filter. Nanotubes, labeled with ATTO 647 N on strand S3, were imaged using the Cy5 filter (excitation and emission wavelength are comparable to ATTO 647N). The image of two different species of single-tile nanotubes in Figure 1g is a superposition of images acquired with the filters for Cy3 and Cy5 dyes individually.

Exposure times for non-encapsulated samples was 90 ms. For encapsulated samples, exposure varied in different experiments, depending on tile concentration and fluorophore used. Exposure times for all two-tile experiments were 3 seconds for all time points. Exposure times for 1x RNA trigger experiment was 1 second for all timepoints except at 24 hours, which had an exposure of 3 seconds. The 4x RNA trigger experiment had an exposure time of 128 ms for all timepoints, as the aggregates formed by crowding of RNA trigger were much brighter than assembled nanotubes. Exposure times for gene titration experiment series were 2-3 seconds. Exposure times for RNase H titration experiments were 2-3 seconds. In SI section S5.8 we report control experiments that demonstrate how exposure time does not affect skewness and kurtosis measurements.

S4.10 Confocal fluorescence microscopy

Confocal laser scanning microscopy was performed at the Advance Light Microscopy/Spectroscopy Laboratory and the Leica Microsystems Center of Excellence at the California NanoSystems Institute at UCLA. Nanotube samples were imaged on a Leica TCS SP8-STED confocal microscope with 63x/1.20 NA water-immersion objective. Images for Cy3 and Atto 647 N labeled samples were obtained with the white light laser and detector set to the maximum excitation/emission wavelengths for Cy3 and Alexa 647, respectively. Droplets were collected in a DNA Lo-bind tube before being loaded into an Ibidi imaging chamber (µ-Slide VI $^{0.1}$, hydrophobic coating) for imaging. Images were line averaged during acquisition, with the number of line-averages noted in the caption of each image, and the z-step size was system optimized to get the maximum number of steps for each z-stack possible.

S4.11 Polyacrylamide gel electrophoresis (PAGE)

Denaturing PAGE: Gel pre-mix was prepared (for a final volume of 100 mL) by adding 42 g of urea to 25 ml of nanopure water, the mixture was then heated until the urea completely dissolved. This mixture was allowed to cool to room temperature, then acrylamide/bis-acrylamide 19:1, 40% solution was added. The pre-mix was added in appropriate ratios with TBE and nanopure water, ammonium persulfate (APS), and Tetramethylethylenediamine (TEMED) to cast the desired polyacrylamide percentage. Gels were cast in 10X10 cm, 1 mm thick disposable mini gel cassettes (ThermoScientific, Cat. No. NC2010) and allowed to polymerize for at least 2 hours before electrophoresis. Gels were run at room temperature at 100 V in 1X TBE unless otherwise noted. Gels were stained in SYBR Gold Nucleic Acid Gel Stain for 20-30 minutes and then imaged using the Biorad ChemiDocTM MP system. We purchased the 10bp DNA ladder used in denaturing gels from Invitrogen™ (Cat. No. 10821- 015).

Non-Denaturing PAGE: Acrylamide/bis-acrylamide 19:1, 40% solution, TAE, Magnesium Chloride 12.5 mM (final concentration), APS, and TEMED were added together at appropriate concentrations for the desired polyacrylamide percentage, then cast in 10X10 cm, 1 mm thick disposable mini gel cassettes (Thermo Scientific, Cat. No. NC2010) and allowed to polymerize for at least 2 hours before electrophoresis. Gels were run at 4°C at 150 V in 1X TBE buffer. After electrophoresis gels were stained in SYBR Gold Nucleic Acid Gel Stain for 20 minutes then imaged using the Biorad ChemiDocTM MP system.

S4.12 RNA extraction

RNA was transcribed using the AmpliScribe-T7-Flash Transcription Kit (Lucigen®). The following components were mixed at room temperature: RNase-free water, 1-1.5mg template, AmpliScribe T7-Flash 1X Reaction Buffer (Cat. No. ASF3507, Lucigen®), 9 mM NTPs, 40 U/mL RiboGuard RNase Inhibitor (Cat. No. RG90925, Lucigen®), and 2mL of AmpliScribe T7-Flash Enzyme Solution (Cat. No. ASF3507, Lucigen®). This mix was then incubated at 37°C for 4 hours.

After transcription, the samples were loaded on a PAGE gel and run at 100V; the gel was then stained in 80 mL 1X TBE and 1mL SYBR Gold Nucleic Acid Gel Stain (Thermo Scientific, Cat. No. S-11494) for 20-30 minutes. The stained gel was placed on a TLC silica gel 60 W F254S aluminum sheet (EMD Millipore, Cat. No. 1055590001) covered in plastic wrap. The gel was then illuminated with UV light and the desired RNA band was excised, crushed and eluted using 200mL of 0.3 M sodium acetate at pH 5.3. The elution reaction was done in 0.5 mL DNA Lobind tubes (Eppendorf, Cat. No. 022431005), incubated at 42°C for ~20 hours. After incubation, the sodium acetate was removed and placed into 1.7 mL RNase/DNase free tubes. The old Lobind tubes were further rinsed with 100mL of 0.3 M sodium acetate pH 5.3, which was added to the new tubes.

Then 1 mL of freezer cold 100% ETOH and 1mL of glycogen was added into each tube and the sample was incubated at -20°C for 20 hours.

Next, the samples were spun at 13500 rpm at 4°C for 15 minutes. The white precipitate pellet (RNA) at the bottom of the tube was located and the supernatant was carefully pipetted out of the tubes to avoid removal of the pellet. Then, 500mL of 70% freezer cold ETOH was added to the tubes and spun at 13500 rpm at 4°C for 5 minutes. The supernatant was carefully decanted again with a pipette. This washing procedure was repeated a third time. After the last wash, as much supernatant was removed as possible, then the tubes were opened and placed in the vacuum concentrator and allowed to spin at room temperature for 15 minutes. The samples were then re-suspended in 10-15mL of Invitrogen™ nuclease free water (ThermoFisher Scientific, Cat. No. AM9932).

S4.13 Droplet detection and pixel brightness value extraction via Python

Extracting pixel brightness values (PBV) data from fluorescence microscopy images by hand is both time consuming and prone to human error. By employing a few basic Python packages, we wrote a script which extracts PBV data from fluorescence microscopy images for hundreds of droplets in minutes. The foundation of the Digital Image Processing (DIP) script we wrote for detecting droplets in epifluorescence microscopy images is built upon the scikit-image image processing library.[5] We implemented the Circular Hough Transform (CHT) algorithm tailored to detect circles across a wide range of radii and remove a majority of artifacts from the returned values.

The droplet detection script is available at Github[6]:

https://github.com/klockemel/dropletDetection_dynamicSelfAssemblyProject.

Before applying the CHT, it is helpful to pre-process the images to improve detection of droplets especially in samples presenting large droplets or aggregation of fluorescently labeled tiles. Prior to running the droplet detection script, all 16-bit .nd2 images are duplicated. Duplicates are brightness-adjusted using the "Auto" setting in the Brightness/Contrast tool of ImageJ and saved as 8-bit tiff files. Brightness-adjusted images are only used for droplet detection, while PBV are extracted from the raw images with full bit depth (Fig S5). Brightness-adjusting the images in which droplets are detected enables a future thresholding step to more accurately separate droplets from the background of the image than would be separated otherwise, even in the presence of bright spots in the image.

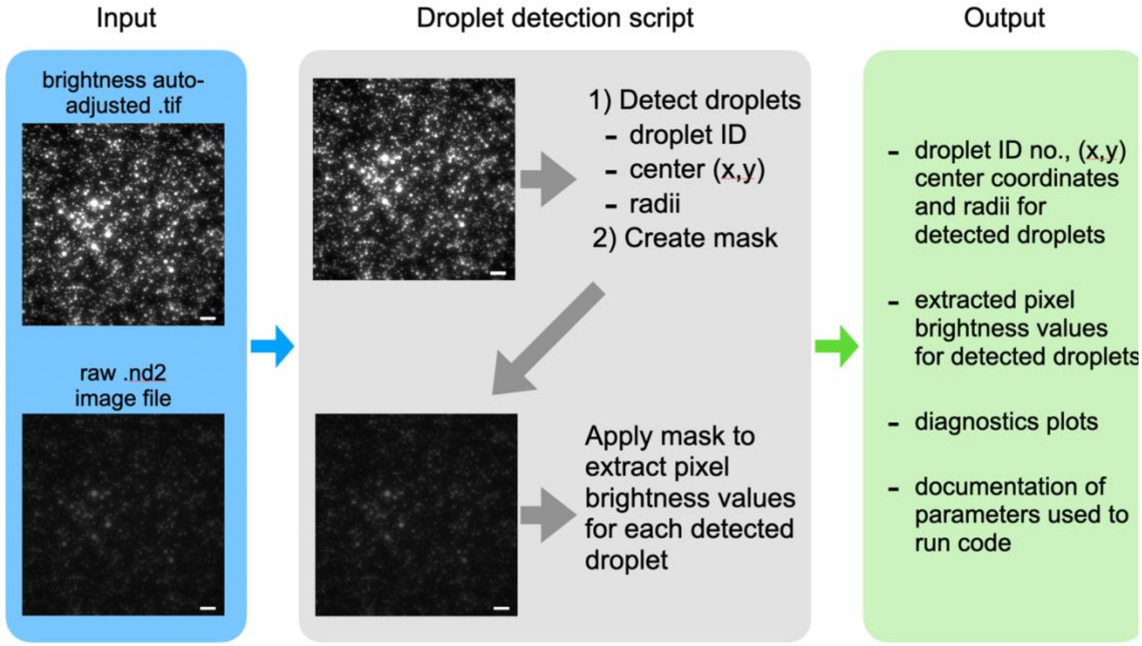

Figure S6: Diagram summarizing the process of the automated droplet detection and data extraction script. First, droplets are detected using the circular Hough transform (CHT) algorithm from the scikit-image package on a brightness-adjusted image. The radii, (x, y) center coordinates, and Hough intensity for each detected circle are filtered to remove circles with low Hough intensities and circles which are overlapping. A mask is created using the detected circles to extract pixel brightness values (PBV) from the raw image file. Diagnostics plots, a reference image with detected droplets labeled, and the information for extracted PBV and circle parameters are the final output. These example images are from the 7.5 nM template co-transcriptional activation of DNA-RNA nanotubes at 0 minutes after encapsulation (Main text Section “Transcriptional control of nanotube assembly inside droplets”). Scalebars are 50 μm.

The droplet detection script requires both the raw and brightness-adjusted images, as well as a few parameters to run. The minimum and maximum radii to search for must be provided in pixels, in addition to the step size. The step size determines which discrete radius values to search for, within the range of the minimum and maximum radii. For a step size of one, all radii between the min and max will be considered, while for a step size of 2, every other value will be skipped, and so on. Droplets made via the shaken protocol described in SI Section S4.7 are heterogeneous in size, and we commonly searched with a minimum of 10 and maximum of 50-70 pixels. As searching over such a large range of radii becomes computationally demanding, radii are broken into subsets of ten for detection with the results from each group compiled for further processing following detection. The final parameter is a limit on the number of droplets allowed in each subset of radii sizes. Varying these parameters determines the computational time,

detection accuracy, and number of droplets returned from sample images. (For example, without a numeric limit to the number of droplets which can be found, the script may return hundreds of artifacts, or false detections, and take upwards of 30 minutes to complete processing an image.)

To detect droplets, the following CHT algorithm is applied over each subset of radii. First a Canny edge detector is applied (skimage.feature.canny). The Canny edge detector function first removes noise from the image via Gaussian smoothing. Gradients in pixel brightness across the image are found using horizontal and vertical Sobel operators, or kernels. Edges are located when pixels lie normal to horizontal, vertical or diagonal gradients with large magnitudes, and weighted according to the magnitude of the neighboring gradient. Edge weights are compared locally and thinned to 1 pixel in width before being thresholded. Pixels connected to edges, with an edge weight value above a smaller threshold are also labeled as edges. The function returns a Boolean image array in which the edges are labeled as True. The CHT transform function (skimage.transform.hough_circle) is then applied to the Boolean edge array. The CHT function in scikit-image returns an array for each radius with the Hough intensities for detected circles. The Hough circle peaks function (skimage.transform.hough_circle_peaks) then reads the Hough circle arrays and returns peak Hough intensity values in the “accums” variable, and the associated (x, y) coordinates and radii for detected droplets.

After detecting circles with the above algorithm, artifacts and overlapping circles are filtered out. First, center (x, y) coordinates and radii for circles with low accums values are dropped. Overlapping circles, often circles sharing the same center coordinates but with different radii, are dropped if the distance between the centers is less than the sum of their radii. The circle with the lower accums value is dropped in this case. Finally, if the number of remaining circles is greater than the user defined limit, the circles with the lowest accums values are dropped until the limit is reached. The results for each subset are compiled to create a list of detected droplets across the range of the minimum and maximum radii parameters. After compiling circles from all radii subsets, overlapping circles and circles with low accums values are once more filtered out.

The center coordinates and radii of the remaining droplets are used to generate a mask labeled with droplet ID, which is then applied to the raw image to extract PBV. A reference image is created in which detected droplets are drawn in red on the brightness-adjusted fluorescence image with their ID numbers. ID numbers are printed in a new color every 100 droplets to help improve legibility for images in which many droplets are detected. The output of the droplet detection script are the reference image with labeled droplets, supplementary diagnostics plots shown in the previous figures, and three comma-separated value (csv) files: 1) the user defined parameters used
in the run, 2) droplet ID number, radii, and center coordinates, and 3) the PBV in the form of a 256-bin list-form histogram with appropriate droplet IDs.

Before PBV data is further processed, reference images are visually inspected, and any remaining artifacts are removed from the final data using their ID numbers. In some cases, detected circles are merely artifacts (as an example, see number 170 in Fig S6b). Other circles which need to be removed from data may have radii off by more than a few pixels (number 197), inaccurate centers (number 175), or are cut-off by the edge of the image.

To quantify our observations of nanotube polymerization and depolymerization we measure the skewness and kurtosis, also respectively known as third and fourth standardized moments, which describe the shape of a distribution. Skewness describes the distribution of any variable about its mean, while kurtosis describes the "tailedness" of a distribution. More specifically, skewness describes where the bulk of a distribution lies relative to its mean, with a negative skew value indicating that the bulk of a distribution is above the mean, a zero skewness value indicates a normal distribution, and a positive skew value indicates the bulk of the distribution is below the mean.[7] Kurtosis is a statistical measure that defines how the tails of a distribution differ from the tails of a normal distribution.[8] We use Fisher's definition to calculate kurtosis (also termed as excess kurtosis), where 3 is subtracted from the result so that kurtosis of the normal distribution is zero.[9] A distribution with heavy tails on either side (ex. Laplace distribution), indicating large outliers, has a positive kurtosis. A distribution with thin tails (ex. Uniform distribution) has a negative kurtosis, indicating fewer outliers in a distribution.

To measure the skewness and kurtosis for the distribution of pixel brightness values extracted for each droplet, first a list of unique pixel brightness values was calculated for each droplet based on the number of pixels in each bin and the bin width of the histogram for each droplet. Then skewness and kurtosis are calculated for the unique pixel brightness values list using the skew and kurtosis functions in the Python Pandas library (pandas.DataFrame.skew and pandas.DataFrame.kurtosis respectively). The average skewness and kurtosis values, along with the standard deviation, were calculated for all droplets measured at each time point of each experiment. Standard deviation was chosen over the variance or other measurements as it represents the spread of the values across the measure samples.

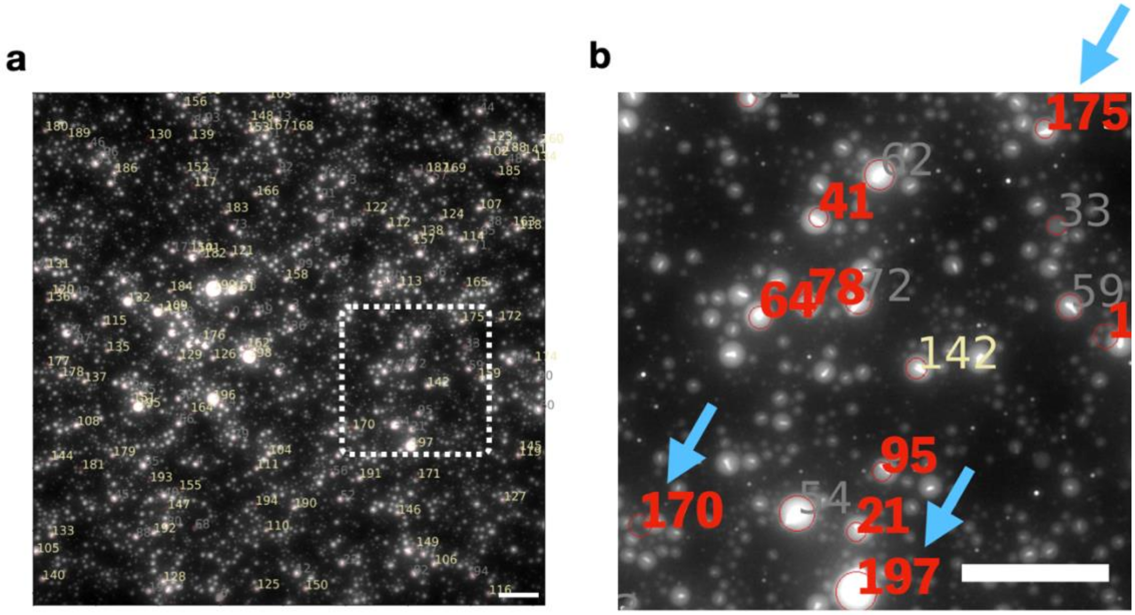

Figure S7: Representative reference images for detected droplets. This is representative image are from experiments in which we used 7.5 nM template for co-transcription of RNA to activate DNA-RNA nanotubes; image is taken at 360 minutes after encapsulation (Main text Section “Transcriptional control of nanotube assembly inside droplets”). (a) The full image with all labeled detected droplets labeled and marked by red circles. (b) An inset of the image marked by the dotted line. Artifacts which are removed before further processing are labeled in bold, red numbers (red font superimposed for illustrative purposes). Blue arrows indicate examples of droplets that were removed (for different reasons) before data analysis. Not only are artifacts removed (170), but also droplets for which the radius is off by more than a few pixels (197), droplets with inaccurately detected center coordinates (175), or a combination of both. Scalebars are 50 µm.

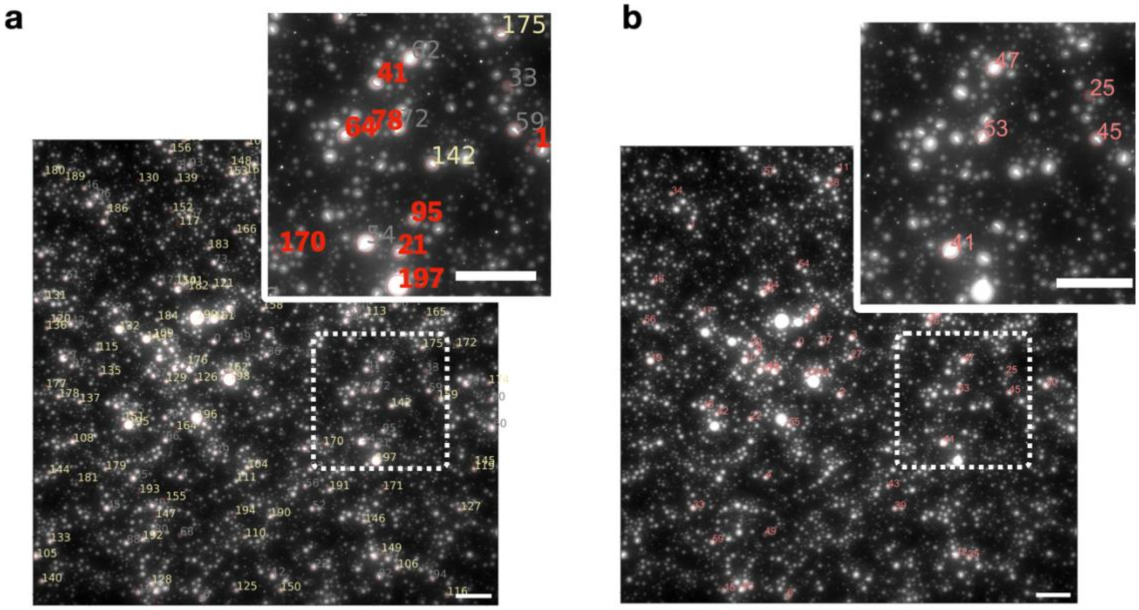

Figure S8: Representative images of detected droplets before (a) and after (b) removing artifacts and incorrectly detected droplets. Detected circles which are removed are indicated by bold, red numbers in inset. Insets are magnified views of the areas indicated by the dotted lines. The images are from the 7.5 nM template co-transcription of DNA-RNA nanotubes at 360 minutes after encapsulation (Main text Section “Transcriptional control of nanotube assembly inside droplets”). Scalebars are 50 μm.

Supplementary Note 5   Additional data and analysis

S5.1 Positioning of droplets inside Ibidi chamber (µ-Slide VI $^{0.4}$)

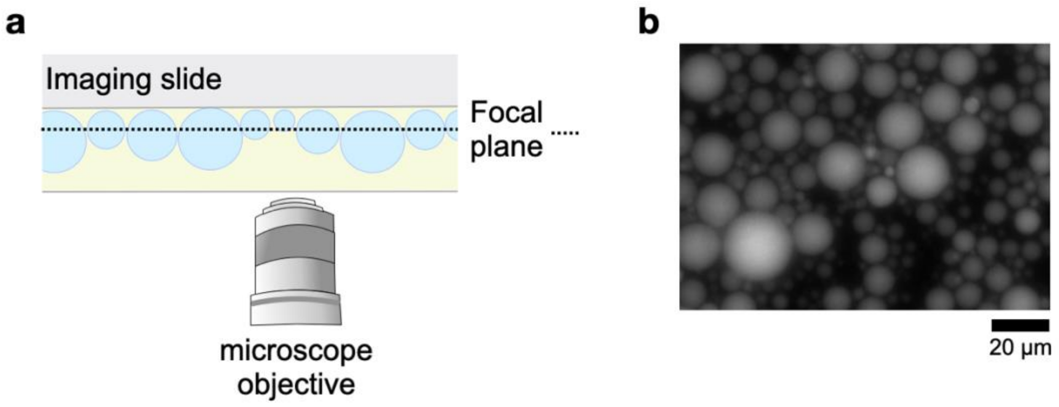

Figure S9: Imaging water-in-oil droplets of different sizes. (a) Cartoon schematic of the cross section of an imaging chamber during observation. As the water droplets (blue) float to the top of the oil phase (yellow), and droplets are not all the same size, the ideal focal plane for imaging each droplet is different. Ideally, we would image near the center of a droplet. (b) Representative fluorescence microscopy image of shaken droplets, immediately after encapsulation. Because the sample is made up of droplets of different sizes, not all droplets can be in focus in a single image. For our experiments, we made the effort to both find a field of view and an optimal focal plane for droplets 6-15 µm in diameter.

S5.2 Morphology of mature two-tile nanotubes after 24 hours of incubation

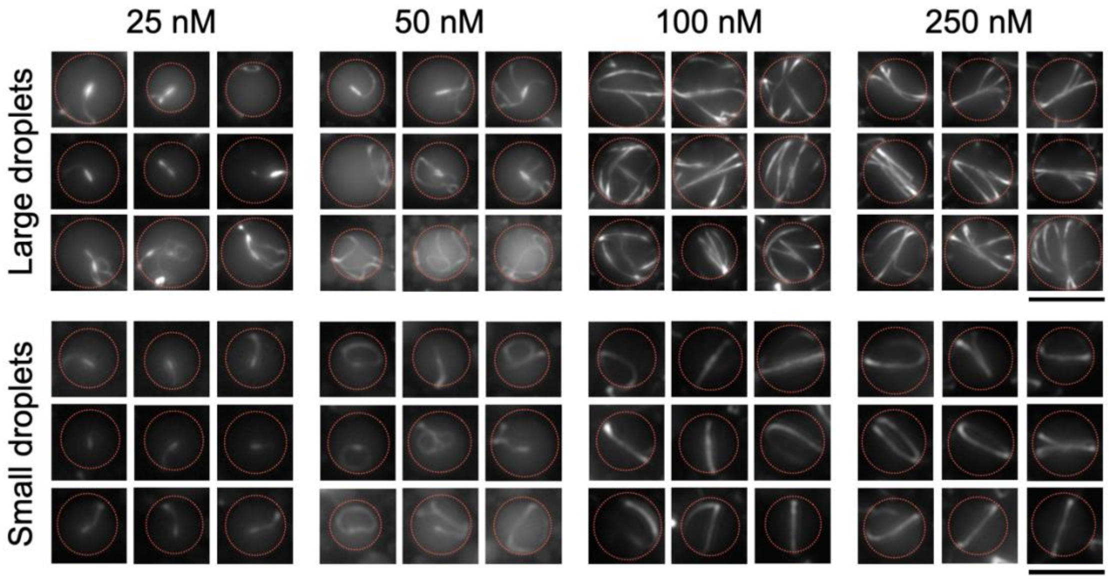

Figure S10: Concentration affects morphology of mature two-tile nanotubes. For very dilute concentrations of 25 and 50 nM, mature nanotubes at 24 h following encapsulation appear more flexible with some small networked or bundled regions. Nanotubes also appear to be distributed throughout the droplet, while ends of some nanotubes localize near the inner surface of the droplets. For higher concentrations, 100 and 250 nM each tile, bundling of nanotubes around the inner circumference of the droplets is clear in droplets 20 µm in diameter and larger, while smaller droplets appear to contain on long nanotube wrapping around the inner surface of the droplet. Confocal microscopy for the 100 nM two-tile sample at 24 h confirms that nanotubes are bundled and wrap around the inner surface of the droplets (figure S11). Scale bars: 25 µm (top) and 15 µm (bottom).

S5.3 Confocal Microscopy images

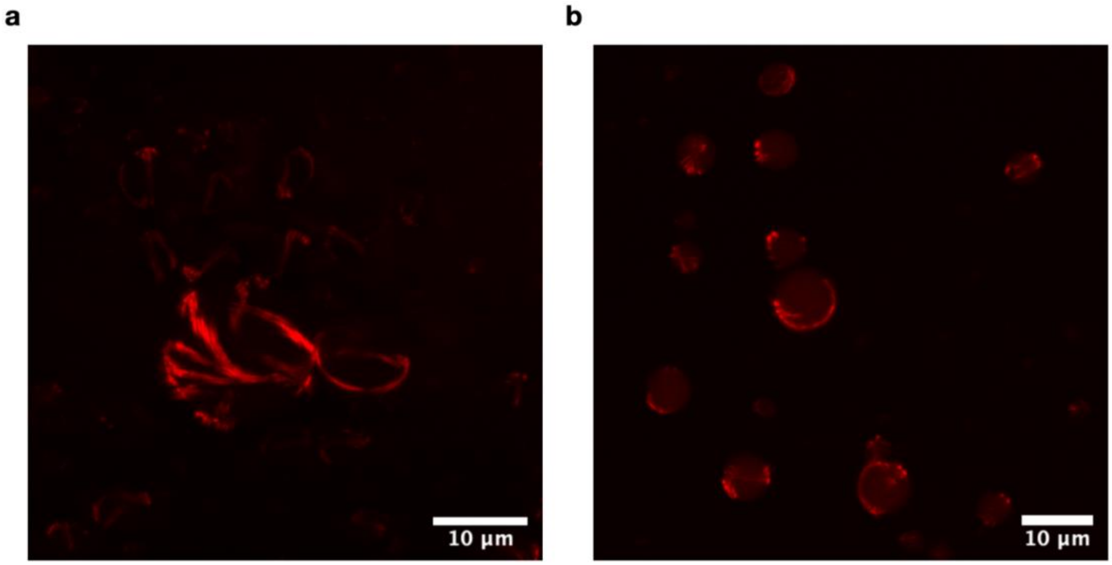

Figure S11: Representative confocal microscopy images of two-tile nanotubes. Here we encapsulated each tile at 100 nM. We are imaging nanotubes at 24 h in water-in-oil droplets without (a) and with 2.5% w/v PEG (b). These results suggest that a majority of the mature nanotubes in this sample are at the surface of the droplets and nanotubes appear to be forming bundle-like structures, as seen in (a). Image (a) is a 6-line average projection, image (b) is a 16-line average projection. See also Supplementary Video 2.

S5.4 Two-tile nanotube system at 25 nM concentration

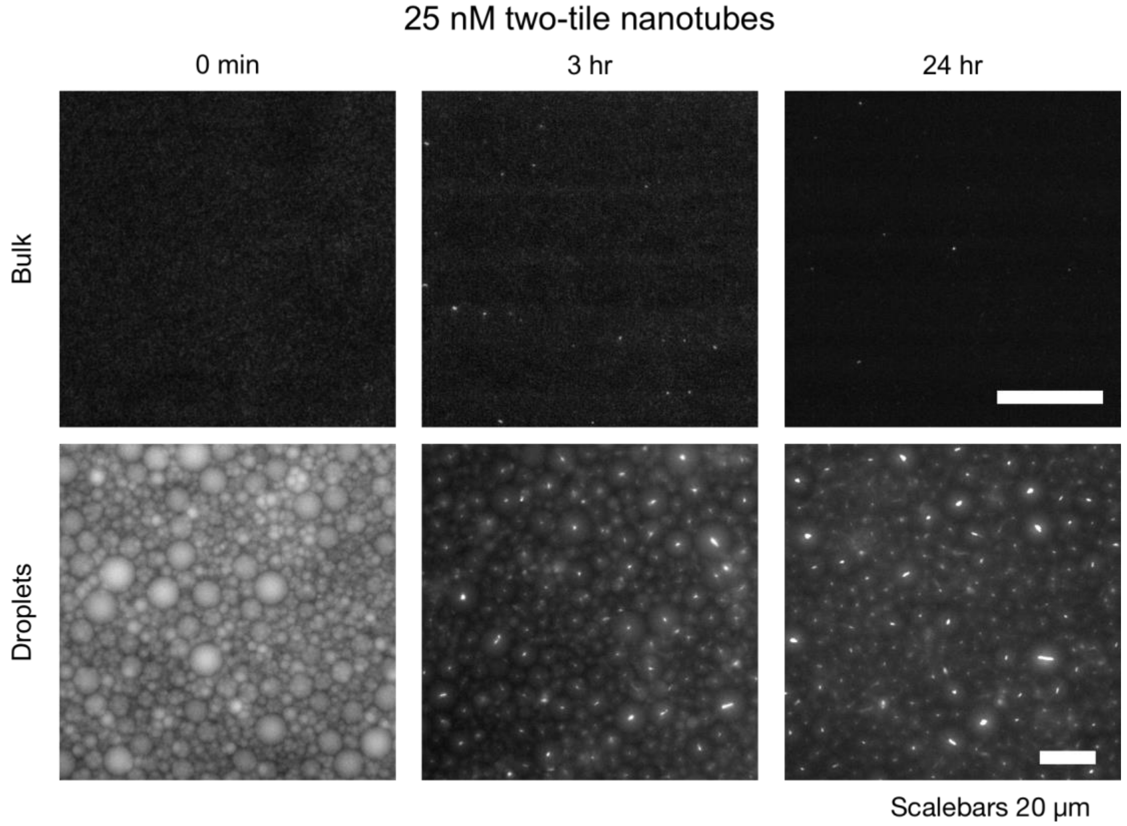

Figure S12: Representative fluorescence microscopy images of 25 nM two-tile nanotubes in non-encapsulated, bulk sample (top) and encapsulated in in water-in-oil droplets (bottom). Here we observe that the nanotubes form inside encapsulated system even if the concentration is too low for a non-encapsulated, bulk setting.

S5.5 DNA-RNA hybrid tiles without gene template

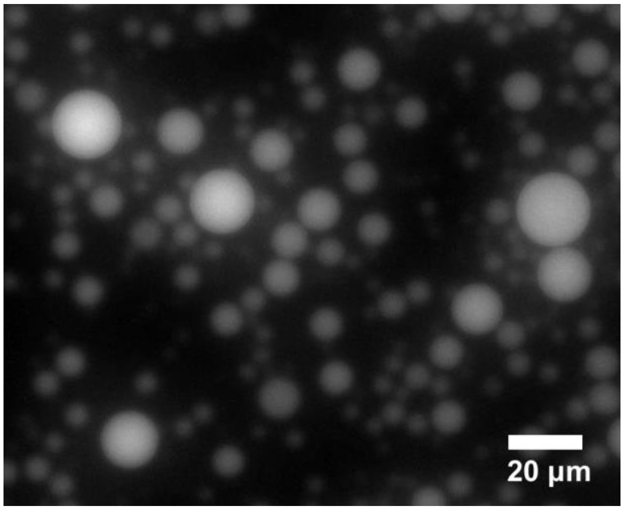

Figure S13: Representative fluorescence microscopy image of DNA-RNA hybrid tiles without the gene template required for production of RNA trigger in droplets. In the absence of the gene template, no assemblies are visible. Inactive tile concentration was 500 nM. Image captured 10 hours after encapsulation.

S5.6 Co-transcriptional assembly without crowding agent

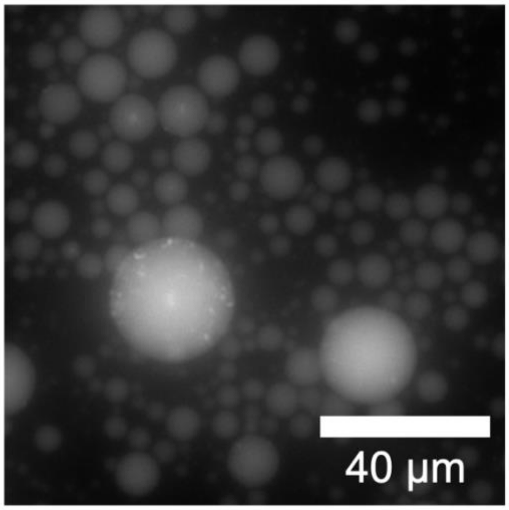

Figure S14: Representative fluorescence microscopy image of DNA-RNA hybrid nanotubes with co-transcription of RNA trigger in droplets without PEG. In the absence of PEG, only few small assemblies are visible. Inactive tile concentration was 500 nM, and template concentration was 100 nM. Image captured 4 hours after encapsulation.

S5.7 Effects of crowding agent on two-tile nanotube system

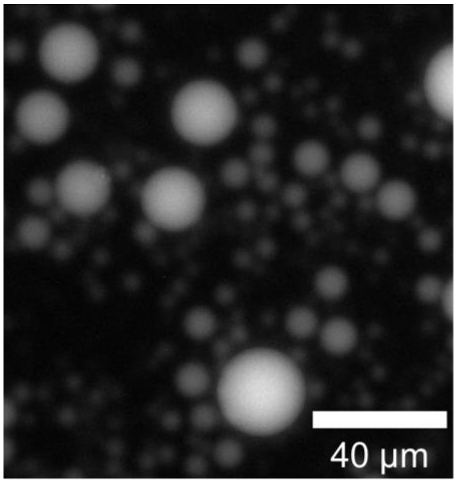

Figure S15: Crowding agents do not cause aggregating of individual tiles in droplets. Representative fluorescence microscopy image of tile A of the two-tile system encapsulated in shaken water-in-oil droplets with 2.5% w/v PEG 180 minutes after encapsulation. As there is only one of the two tiles in the droplets, no nanotube polymerization occurs.

S5.7 Influence of PEG on transcriptionally activated nanotubes

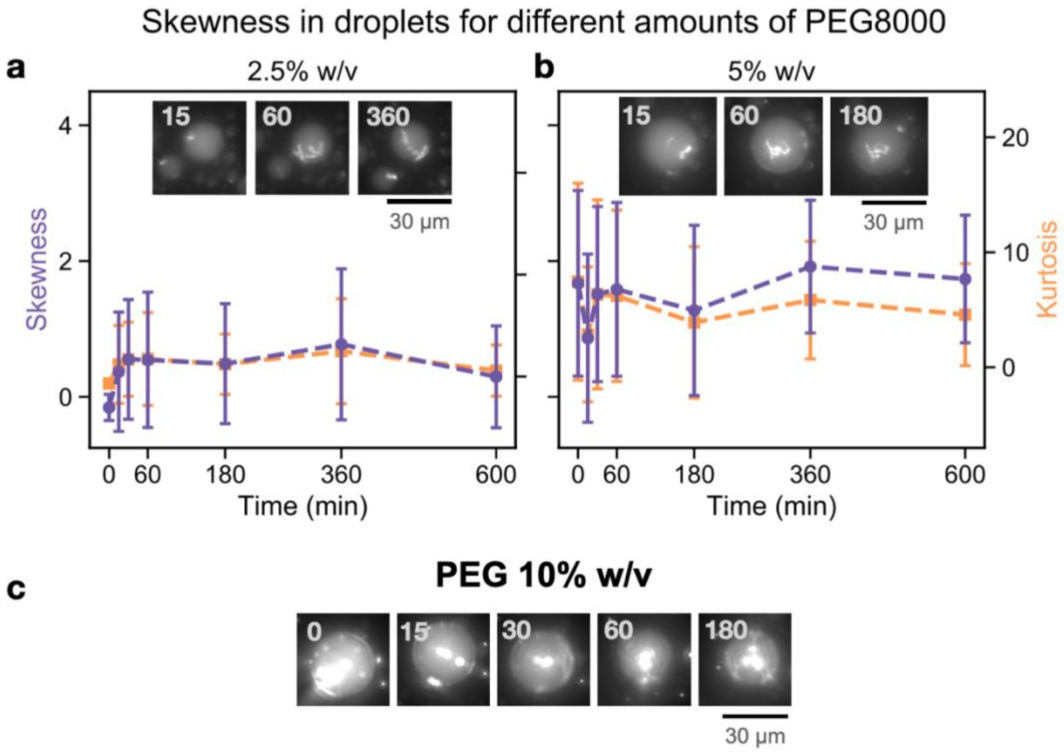

Figure S16: Representative fluorescence microscopy images (insets) showing the influence of PEG on transcriptionally activated nanotubes. We show mean skewness and kurtosis measurements at varying amounts of PEG in droplets (2.5% w/v in (a) and 5% w/v in (b)). Inactive tile concentration was 500 nM, and template/gene concentration was 100 nM. (c) Droplets could not be detected for 10% w/v PEG assay. Data is presented as mean values +/- standard deviation. The number of droplets sampled and a histogram data of radii for sampled droplets in SI section S5.9 and S5.10. Scale bars: 30 µm.

S5.8 Influence of exposure time on skewness and kurtosis

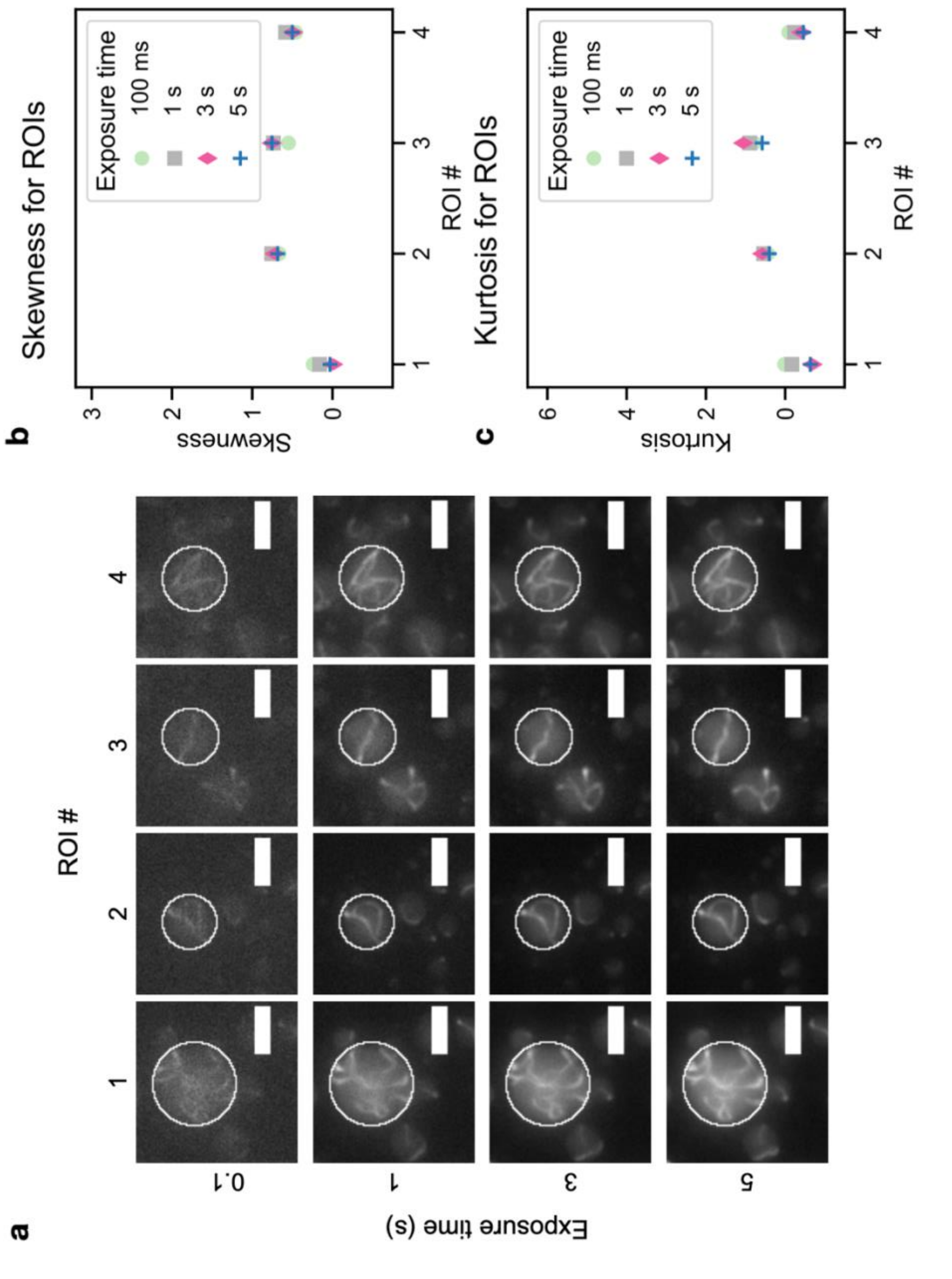

Figure S17: Skewness and kurtosis are not significantly influenced by exposure time. In this control experiment, we show computed skewness and kurtosis for different exposure times over four different droplets from a repeated assay of the 100 nM two-tile nanotubes incubated at room temperature. The images processed are from 3 h into the experiment. (a) Fluorescence microscopy images of the droplets, or Regions Of Interest (ROIs), used for the control measurements. Four droplets were chosen in which the nanotubes did not appear to move between the different images. Four exposure times

were chosen that represented a range of exposure times used for assays in this work – 100 ms, 1, 3, 5 s. Pixel brightness values were extracted for each droplet, or ROI, from images captured with each exposure time, and skewness (b) and kurtosis (c) values were calculated from there. Skewness and kurtosis values are close across all exposure times. For ROI number 1 and 3, there is a slight difference in skewness and kurtosis, but close inspection of the nanotubes within those droplets suggest there may have been some shifting within the droplet between images captured with exposure times that varied. As no skewness and kurtosis for exposure times were consistently different for all droplets measured, we conclude that data captured with different exposure times is comparable provided the image is not over- or under-exposed. As shown in figure S18, the 100 ms exposure is slightly under-exposed, but skewness and kurtosis values do not appear drastically affected. Data for this figure were extracted by hand using ImageJ. Scale bars 10 µm.

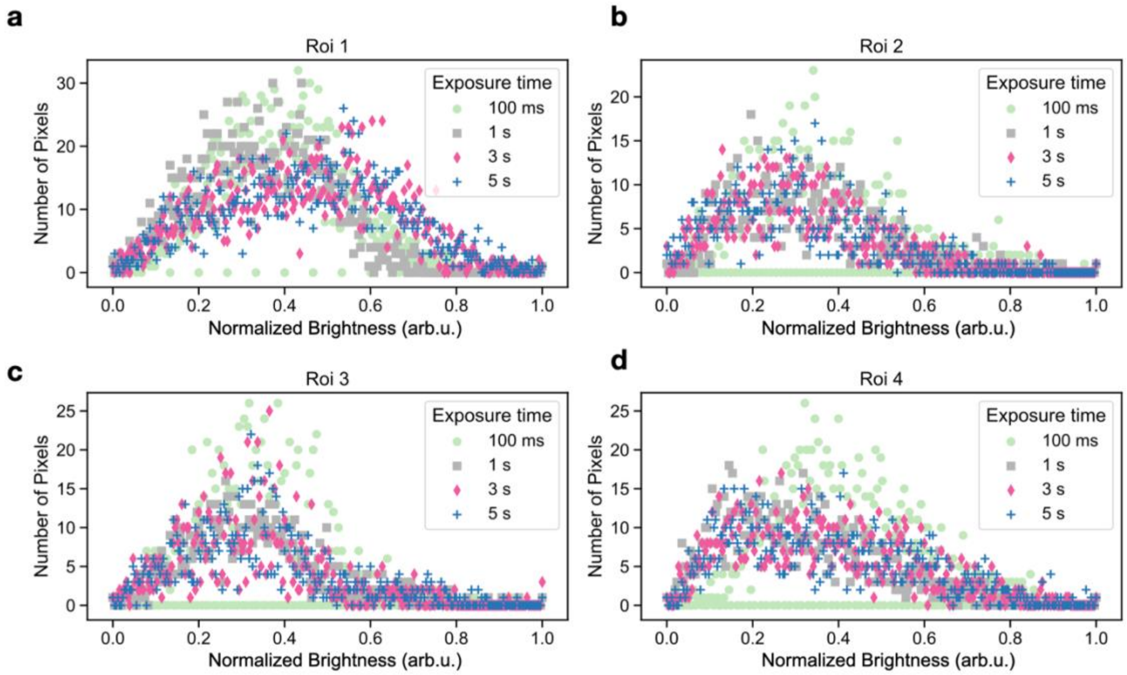

Figure S18: Normalized brightness of the pixels detected in each ROI with different exposure time: (a-d) Normalized distributions of pixel brightness values for the same four droplets, or ROIs, at each of the four exposure times discussed in figure S12. (Normalization formula: $I\ normalized = \frac{(I - I\ minimum)}{(I\ maximum - I\ minimum)}$). From these distributions, we can see that the 100 ms exposure is slightly under-exposed, as there are a number of the 256 bins of pixel brightness values which are empty. There is a difference in the shape of the distributions for ROI 1, which is reflected in the slight disagreement of the skewness and kurtosis values between the 100 ms and 1 s exposures, and the 3 s and 5 s exposures. We also see a difference in distribution shape between the 100 ms exposure and the 1, 3 and 5 s exposures for ROI 3. In this study we do not observe a consistent relation between exposure time and the shape of the pixel brightness histogram across all four droplets. Hence, it is likely slightly disagreeing measurements are due to some small shift in the nanotube position inside the droplets, and comparing the skewness and kurtosis values for images captured with different exposure times is valid provided no droplets are under- or over-exposed.

S5.9 Number of droplets detected in each experimental study

Here we provide the number of droplets selected for computing the skewness and kurtosis in each experiment. The number of droplets vary at each time point in an experimental study because of reasons as described in the droplet detection method (S4.13). As our experiments did not include labeled oligomers or dyes as fluorescence references, and droplets were identified with an edge-based detection algorithm, detection becomes less accurate for samples in which most of the tiles have been recruited to nanotubes. For this reason, as the free tiles in droplets decrease, the number of droplets detected and processed also decreases. Thus, as time progresses, the number of droplets detected by the method reduce. Inclusion of a secondary inert dye, not involved in the nanotube polymerization process would address this limitation and provide consistency in droplet detection and processing for all stages of the experiment. As skewness and kurtosis are affected by the distribution of pixel brightness values, out-of-focus images would have different skewness and kurtosis values than in-focus images of the same subject. For experiments in which the 0 min image is out-of-focus, we processed the data from 15 minutes, the next time-point, onwards.

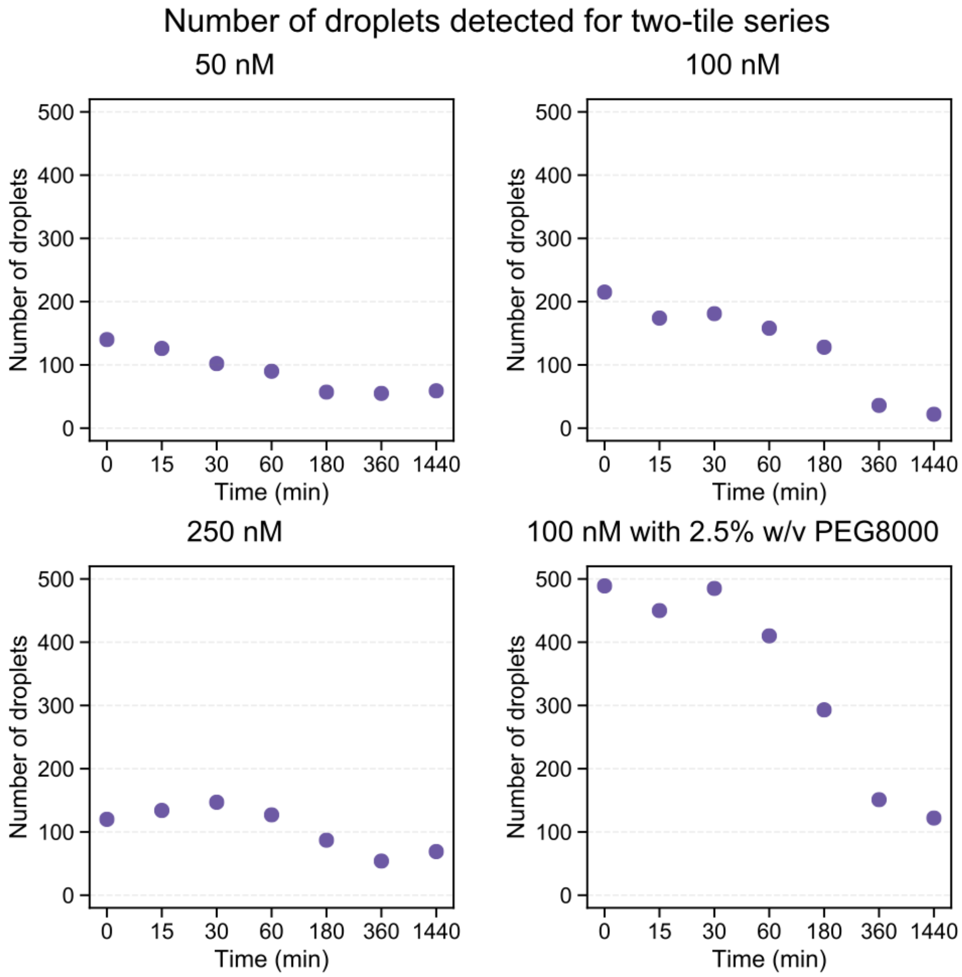

Figure S19: Number of droplets detected using the script from SI Section 4.13 for two-tile experiments in Figure 3 of the main text.

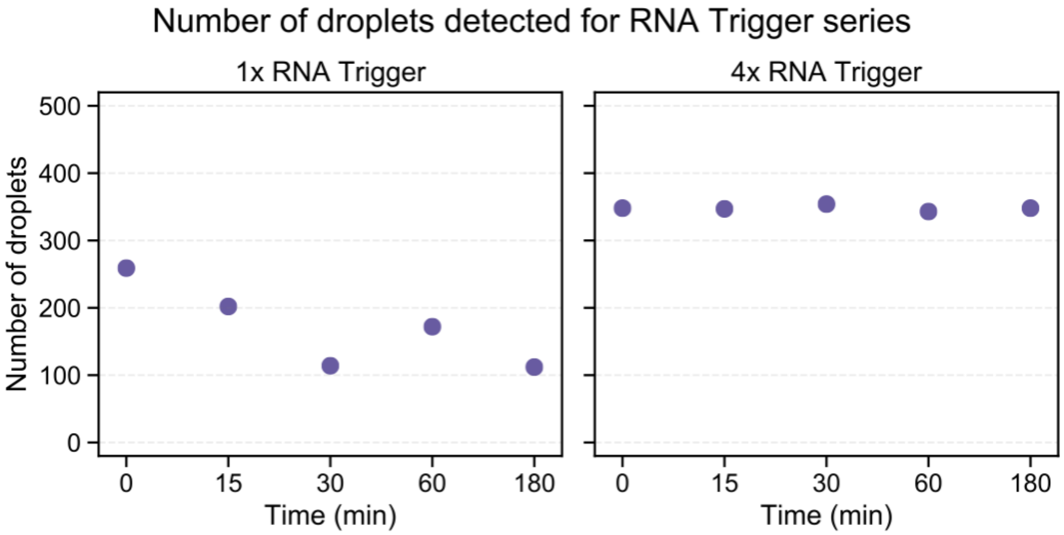

Figure S20: Number of droplets detected using the script from SI Section 4.13 for the DNA-RNA hybrid nanotubes with gel-extracted RNA trigger experiments (Figure 4).

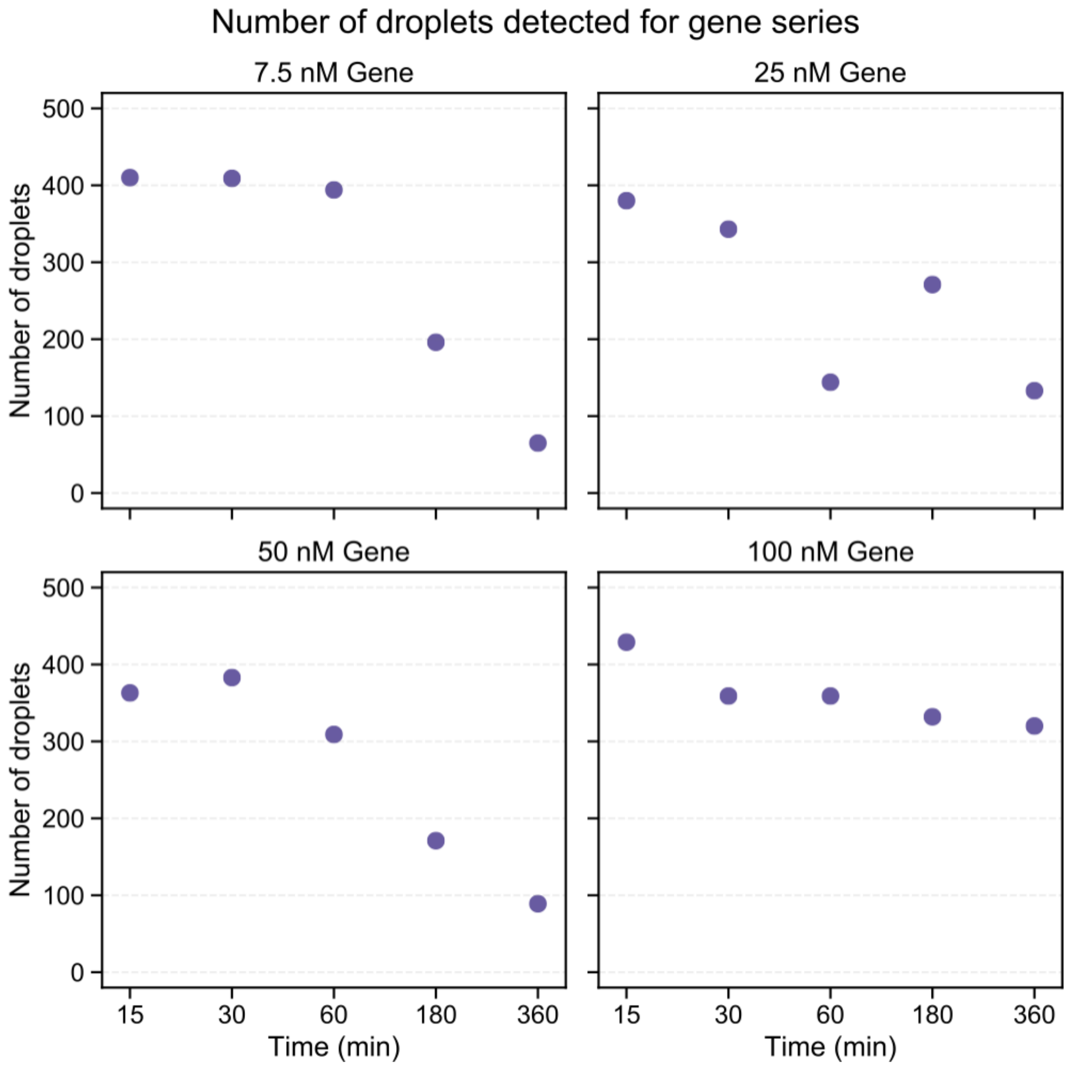

Figure S21: Number of droplets detected using the script from SI Section 4.13 for the DNA-RNA hybrid nanotubes with varying concentrations of template, or gene, experiments (Figure 5).

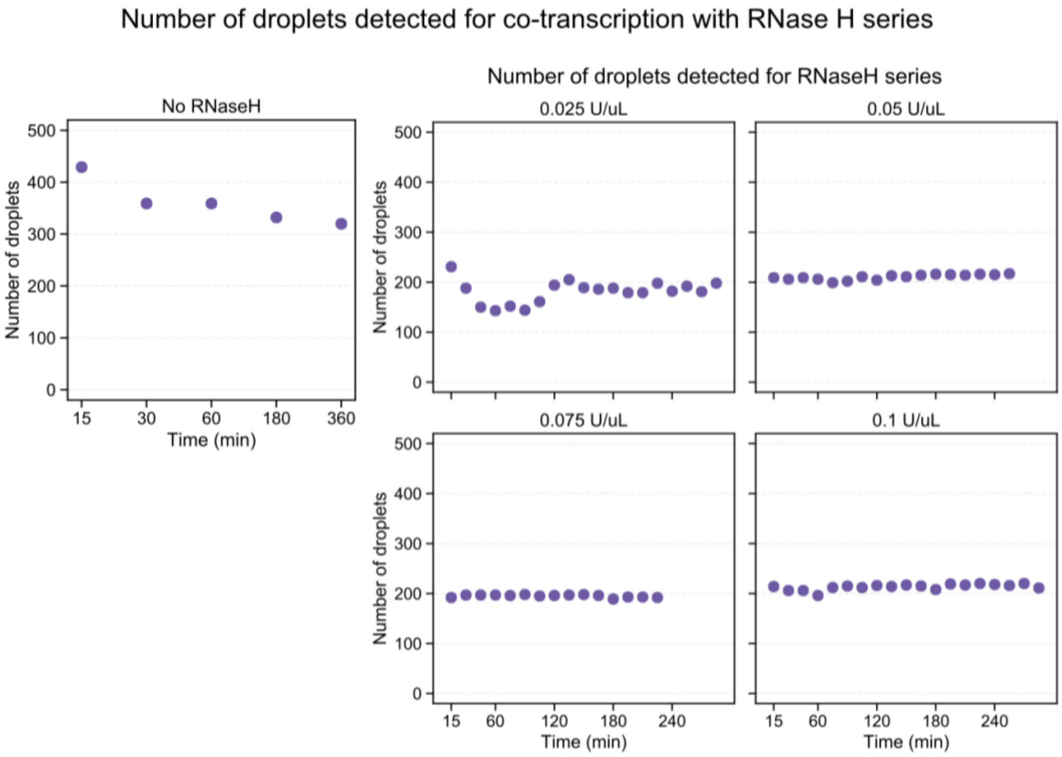

Figure S22: Number of droplets detected using the script from SI Section 4.13 for the DNA-RNA hybrid nanotubes with varying concentrations RNase H experiments (Figure 6). “No RNase H” data is the same as “100 nM Gene” from figure S21.

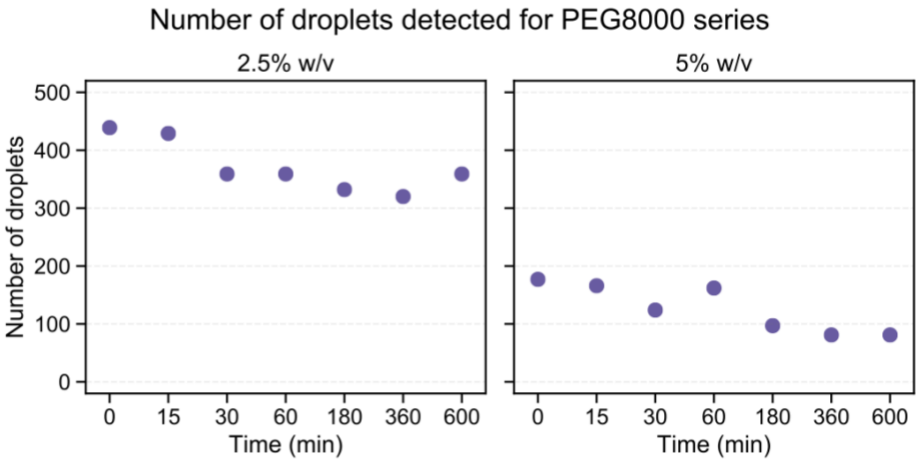

Figure S23: Number of droplets detected using the script from SI Section 4.13 for the DNA-RNA hybrid nanotubes with varying concentrations of PEG experiments (Figure S16). Droplets were not tracked for the duration of the experiment.

S5.10 Radii of the detected droplets in each experimental study

Here we provide the histograms of the radii of droplets selected for computing the skewness and kurtosis in each experiment. The droplet radii vary in each experimental study because the shaken protocol for water-in-oil droplet method (S4.7) produces a wide range of droplet sizes. Differences in detected droplet sizes can also occur in the droplet detection step (S4.13).

Another reason for omitted detection of droplets can be if they are overlapping inside the imaging chamber. Images collected of such droplets have fluorescent signal from multiple droplets on top of each other in the z-plane. Thus, individual droplets become indistinguishable in that area and have to be discarded in order to get the most accurate skewness and kurtosis value. We did not encapsulate a reference dye or fluorescently labeled oligomer to aid in the detection of droplets.

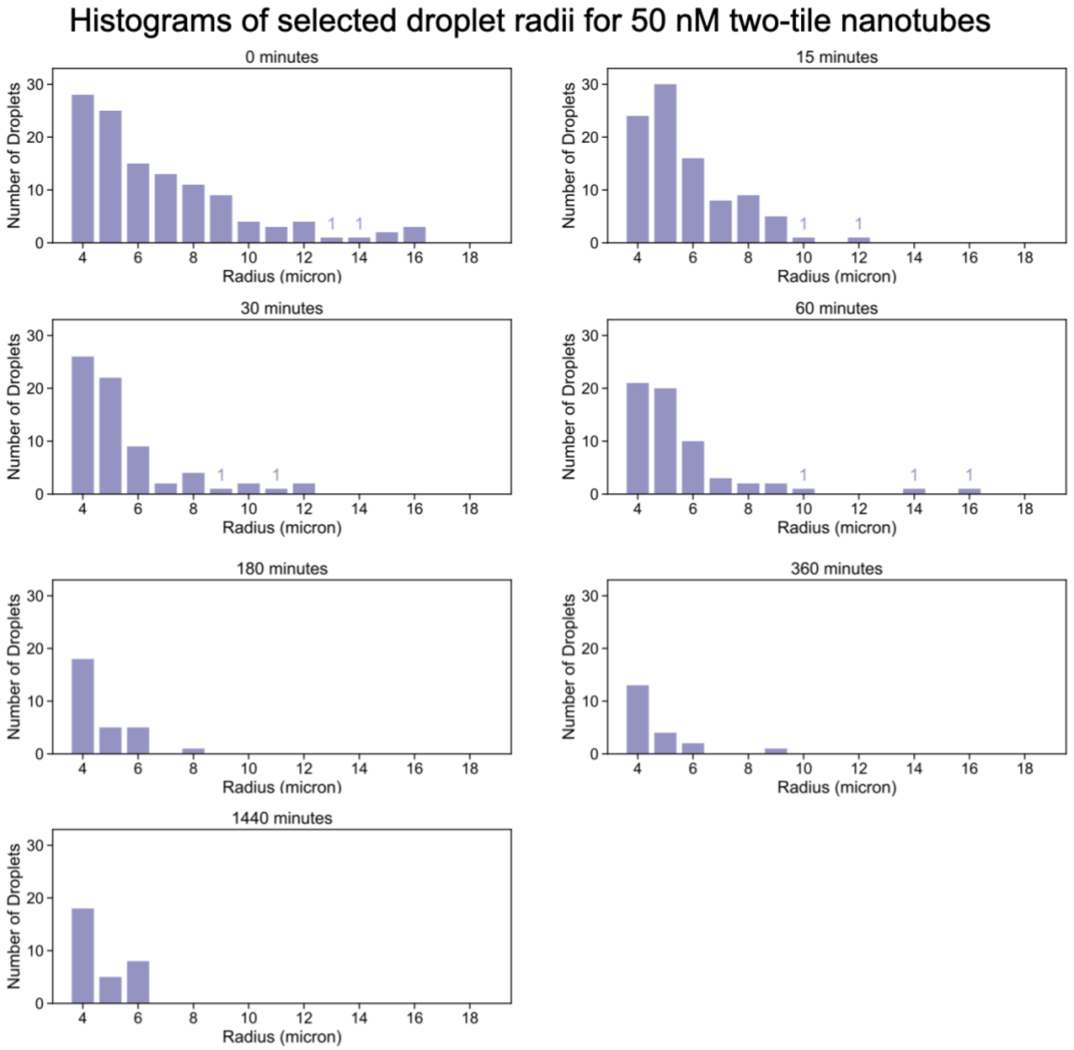

Figure S24: Histograms of the radii of measured droplets for the 50 nM two-tile nanotubes in water-in-oil droplets experiment (Figure 3). Droplets were detected with the script described in SI Section 4.13.

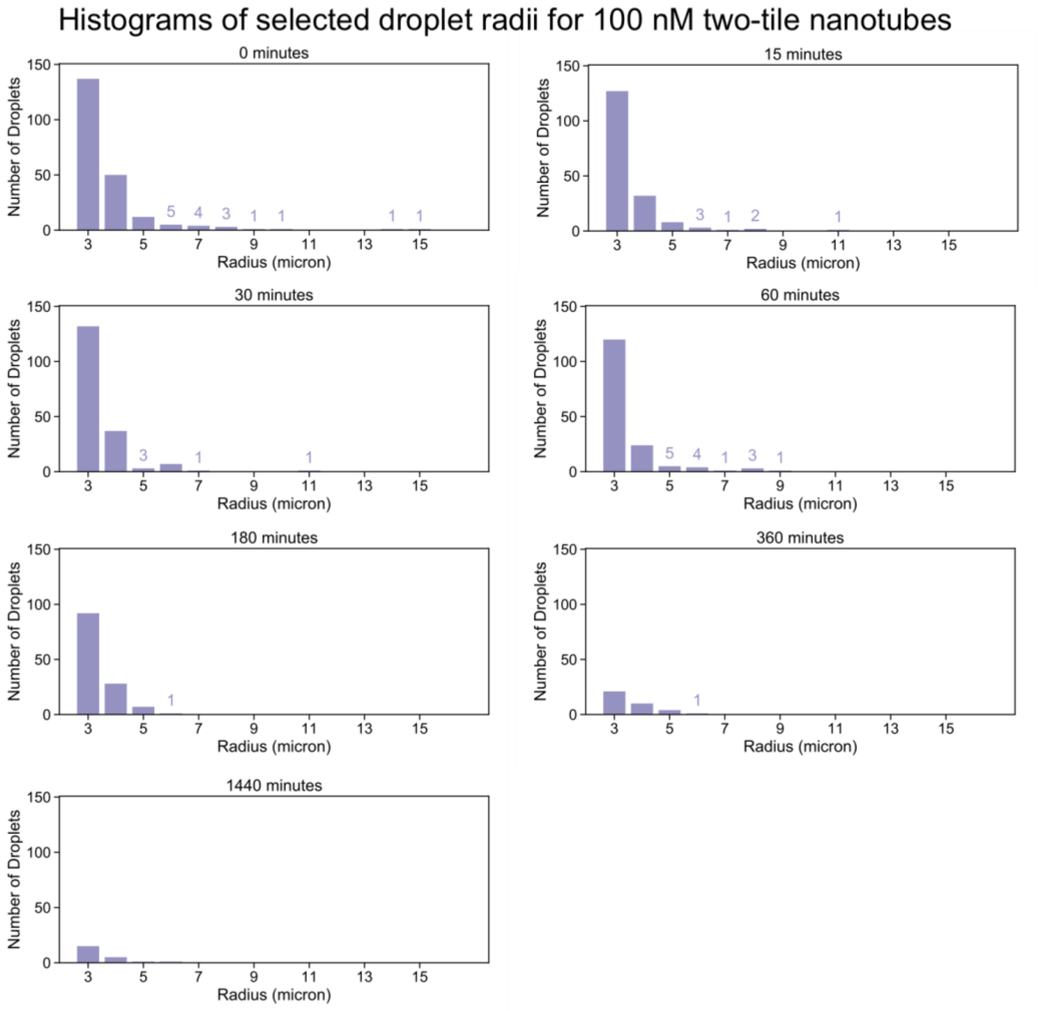

Figure S25: Histograms of the radii of measured droplets for the 100 nM two-tile nanotubes in water-in-oil droplets experiment (Figure 3). Droplets were detected with the script described in SI Section 4.13.

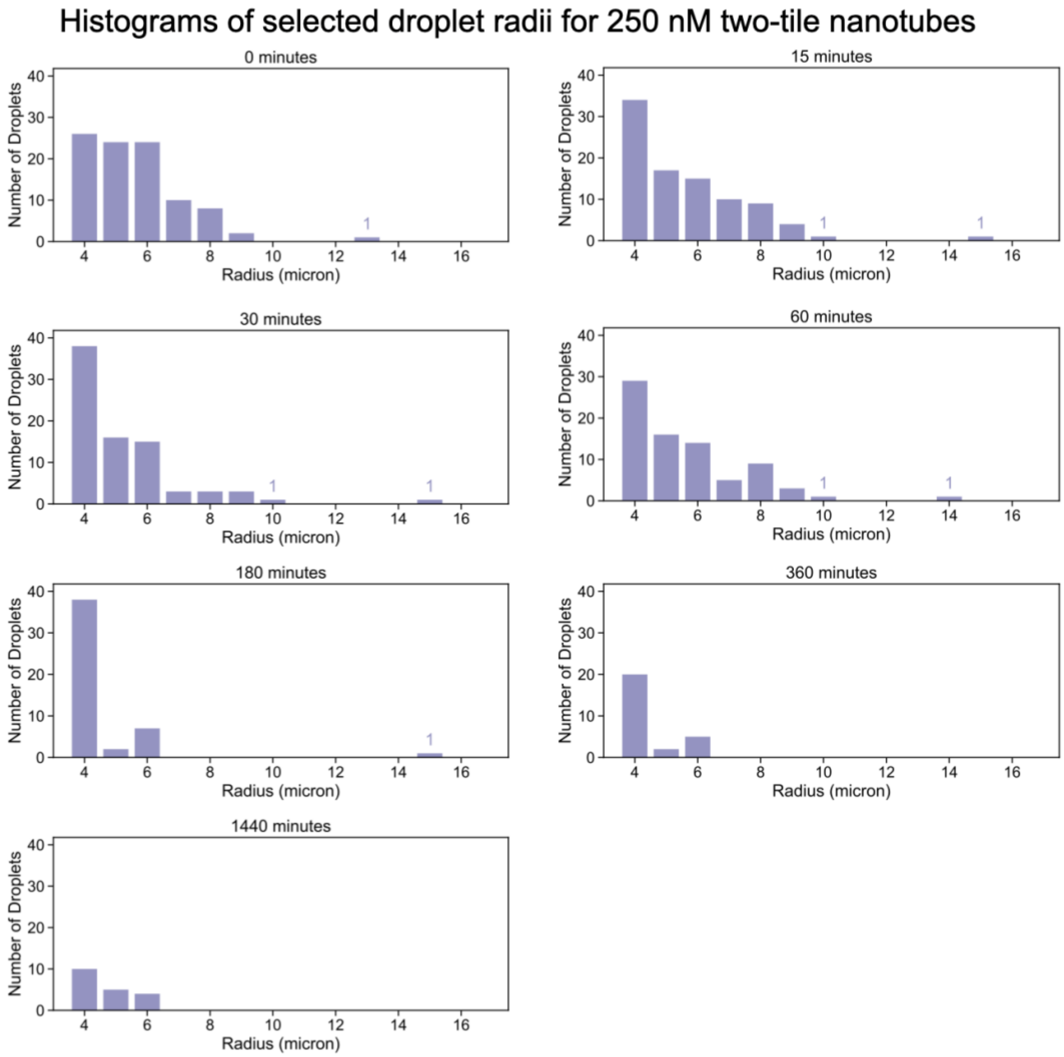

Figure S26: Histograms of the radii of measured droplets for the 250 nM two-tile nanotubes in water-in-oil droplets experiment (Figure 3). Droplets were detected with the script described in SI Section 4.13.

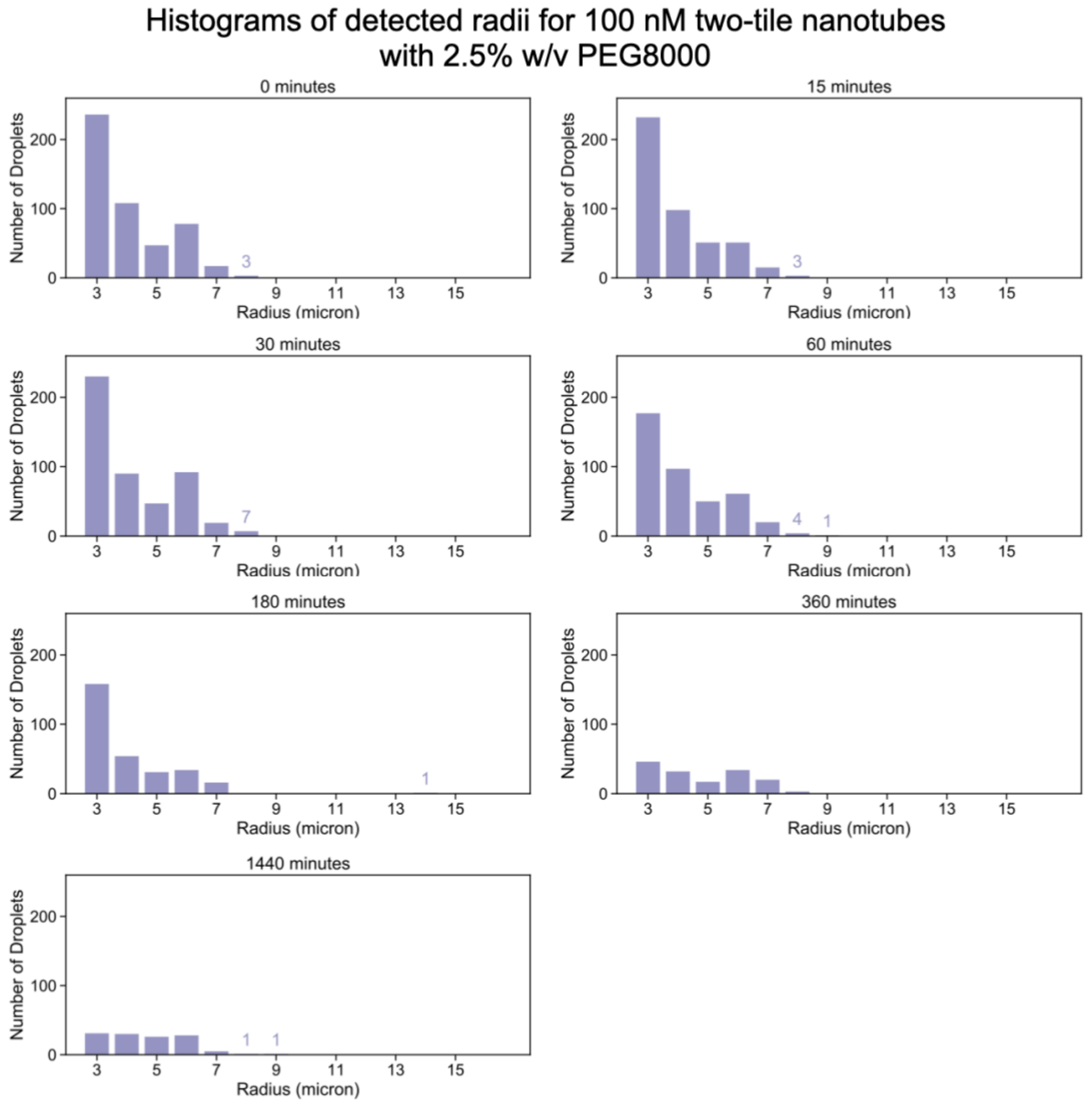

Figure S27: Histograms of the radii of detected droplets for the 100 nM two-tile nanotubes with 2.5% w/v PEG in water-in-oil droplets experiment (Figure 3). Droplets were detected with the script described in SI Section 4.13.

Histograms of detected radii for 1x RNA Trigger

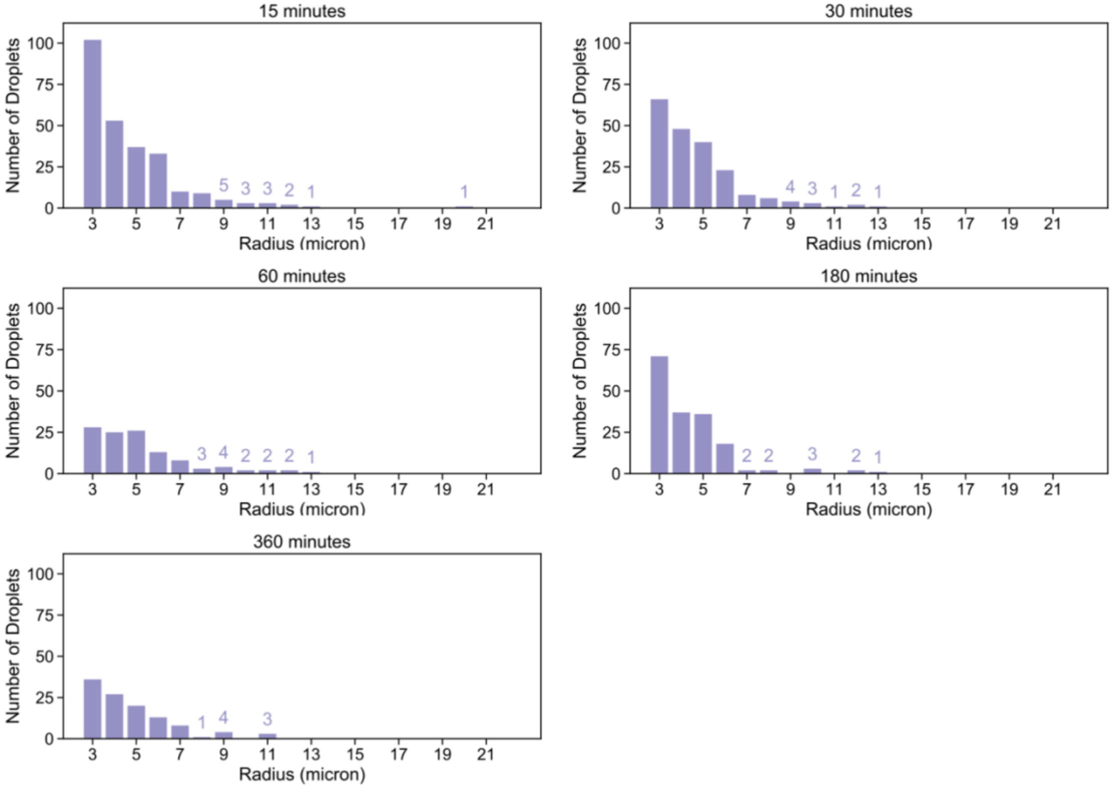

Figure S28: Histograms of the radii of detected droplets for the DNA-RNA hybrid nanotubes with 1x gel-extracted RNA trigger in water-in-oil droplets experiment (Figure 4). Droplets were detected with the script described in SI Section 4.13.

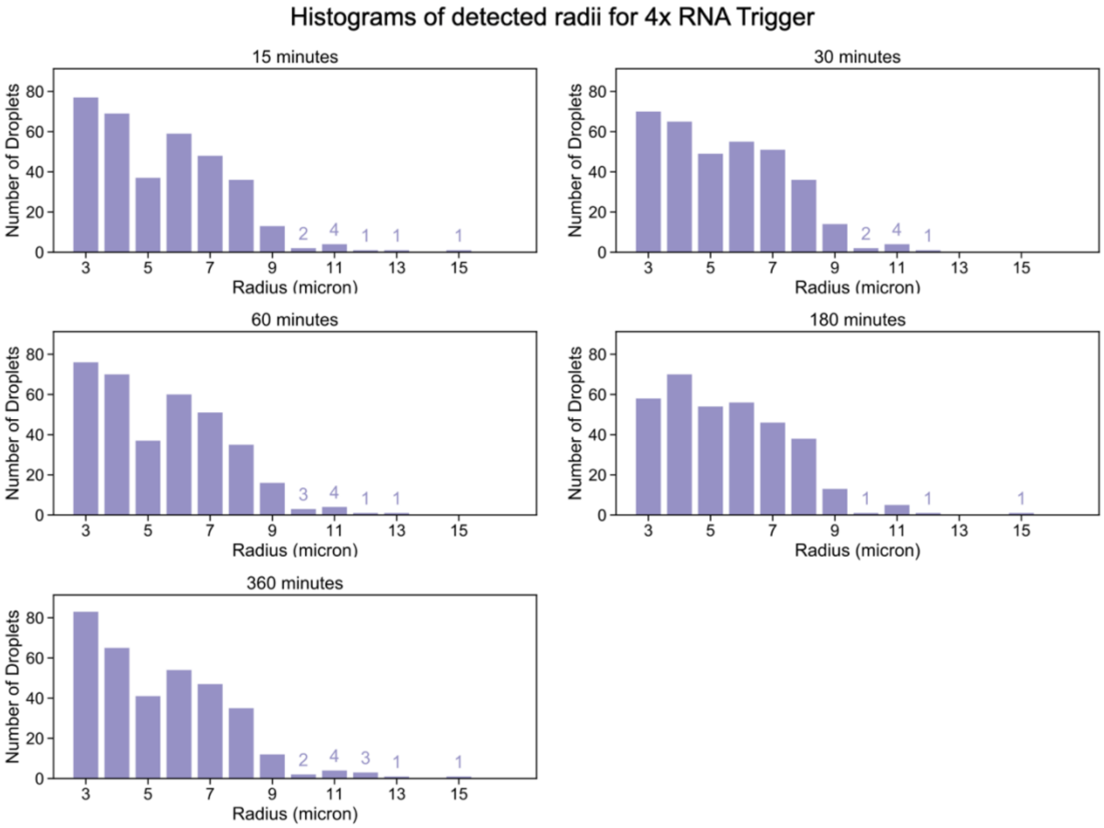

Figure S29: Histograms of the radii of detected droplets for the DNA-RNA hybrid nanotubes with 4x gel-extracted RNA trigger in water-in-oil droplets experiment (Figure 4). Droplets were detected with the script described in SI Section 4.13.

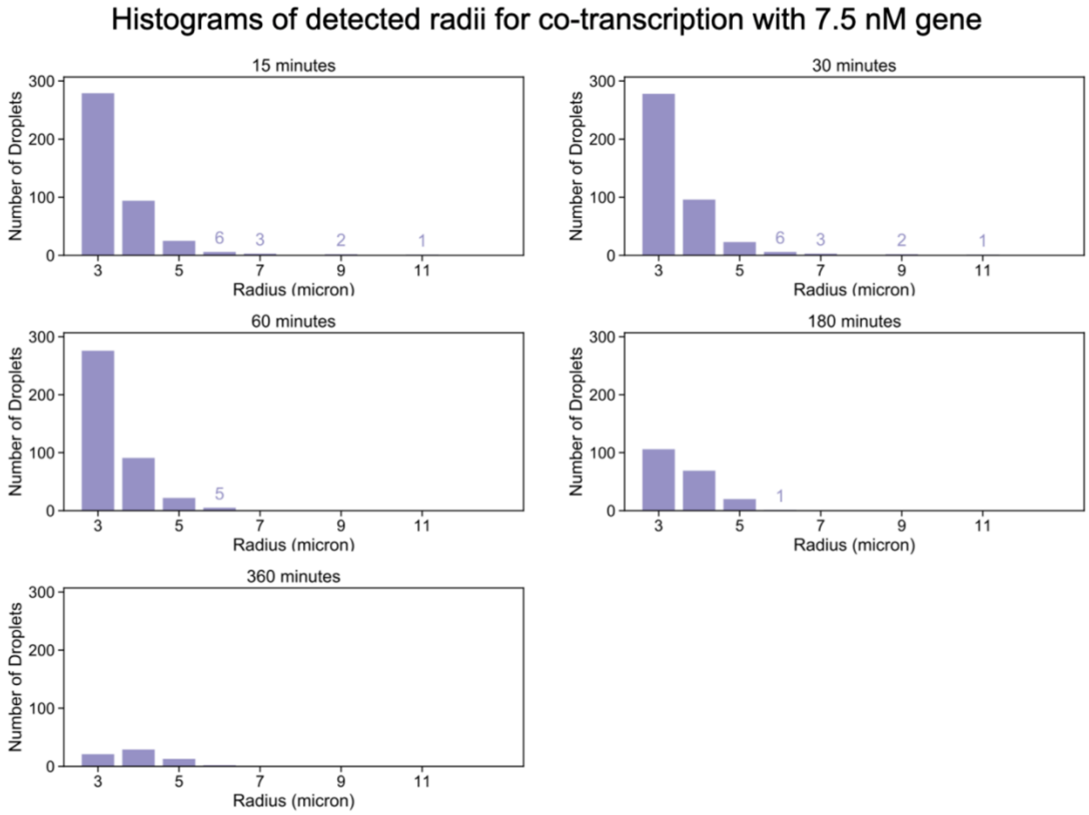

Figure S30: Histograms of the radii of detected droplets for the DNA-RNA hybrid nanotubes with co-transcription of RNA trigger from 7.5 nM template/gene in water-in-oil droplets experiment (Figure 5). Droplets were detected with the script described in SI Section 4.13.

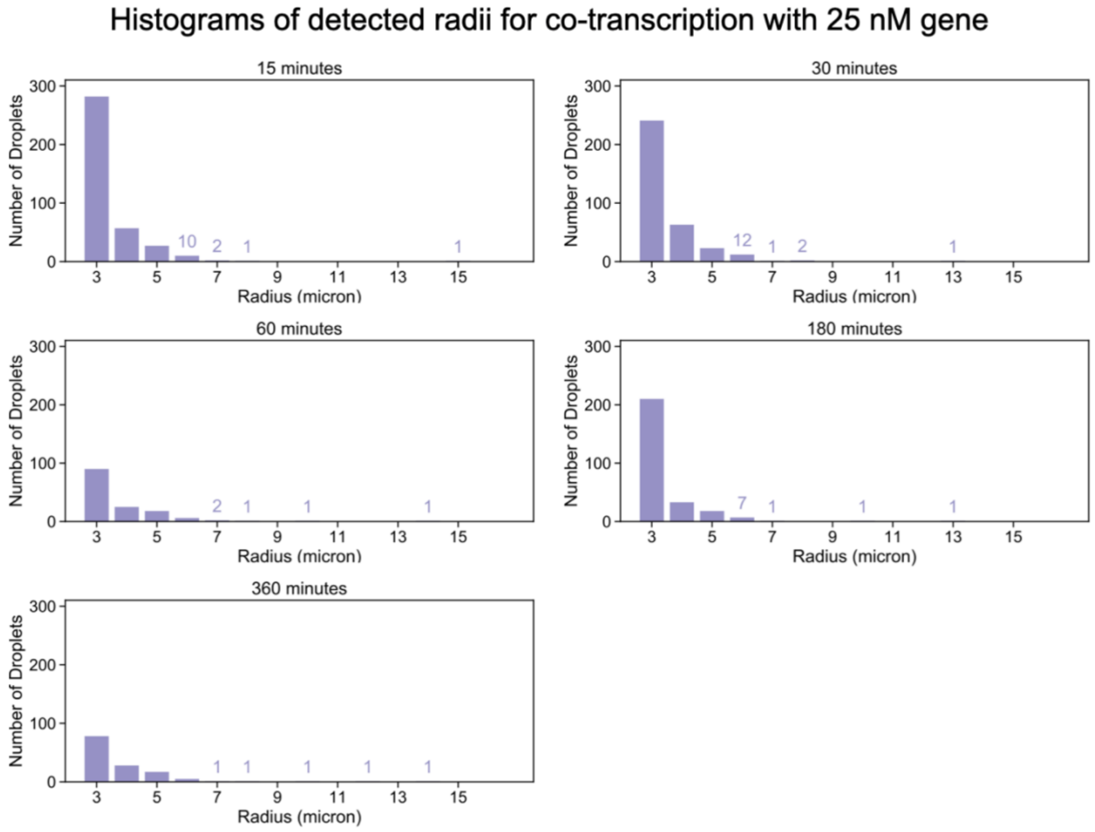

Figure S31: Histograms of the radii of detected droplets for the DNA-RNA hybrid nanotubes with co-transcription of RNA trigger from 25 nM template/gene in water-in-oil droplets experiment (Figure 5). Droplets were detected with the script described in SI Section 4.13.

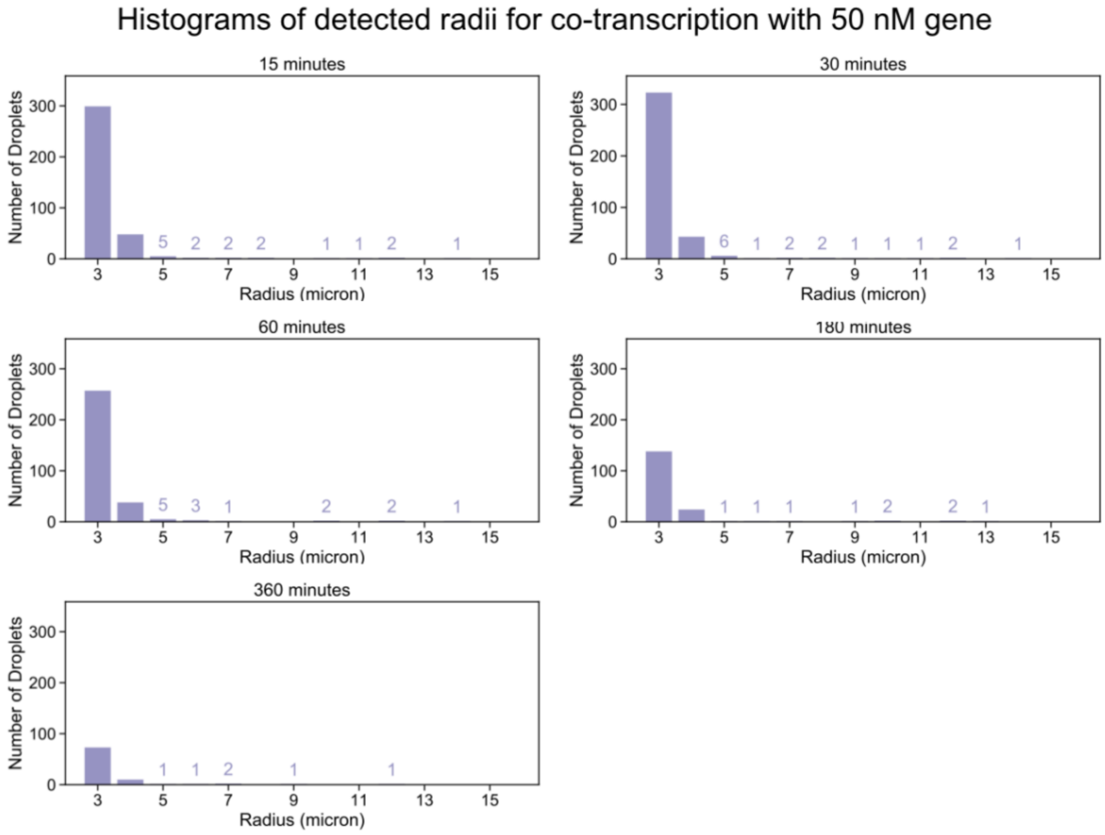

Figure S32: Histograms of the radii of detected droplets for the DNA-RNA hybrid nanotubes with co-transcription of RNA trigger from 50 nM template/gene in water-in-oil droplets experiment (Figure 5). Droplets were detected with the script described in SI Section 4.13.

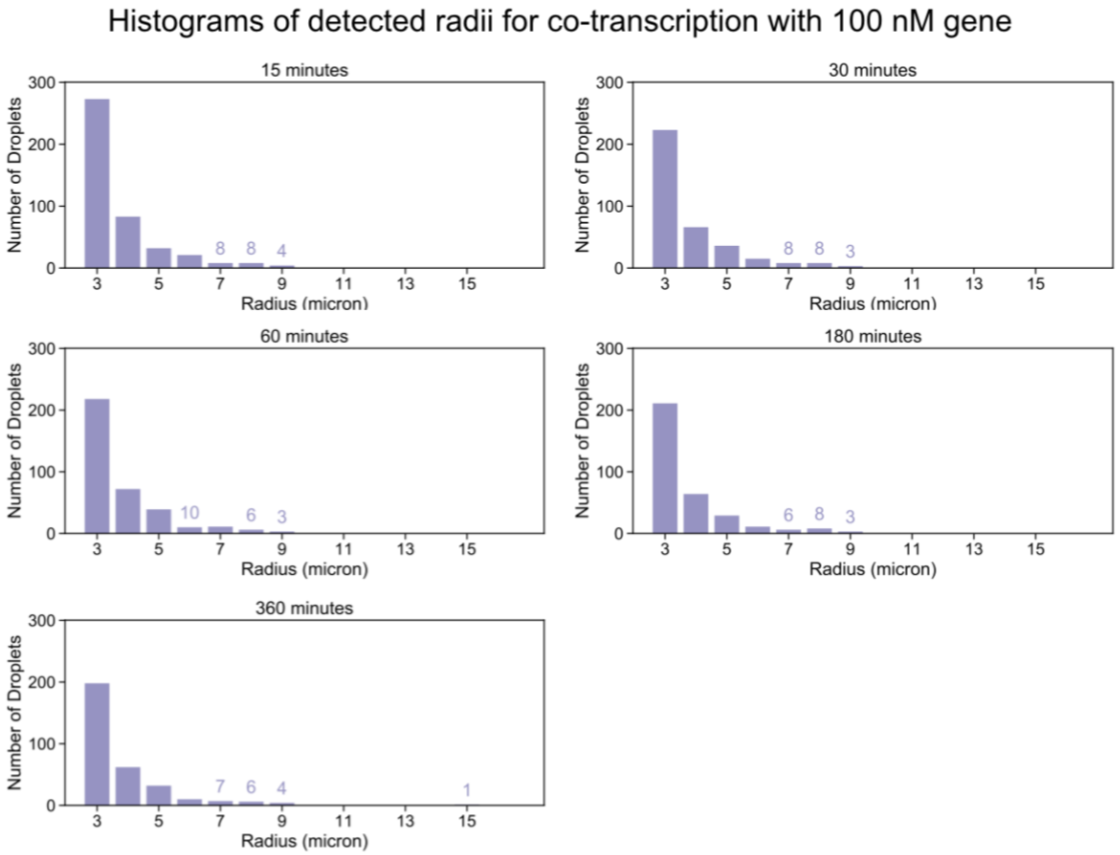

Figure S33: Histograms of the radii of detected droplets for the DNA-RNA hybrid nanotubes with co-transcription of RNA trigger from 100 nM template/gene in water-in-oil droplets experiment (Figure 5). Droplets were detected with the script described in SI Section 4.13.

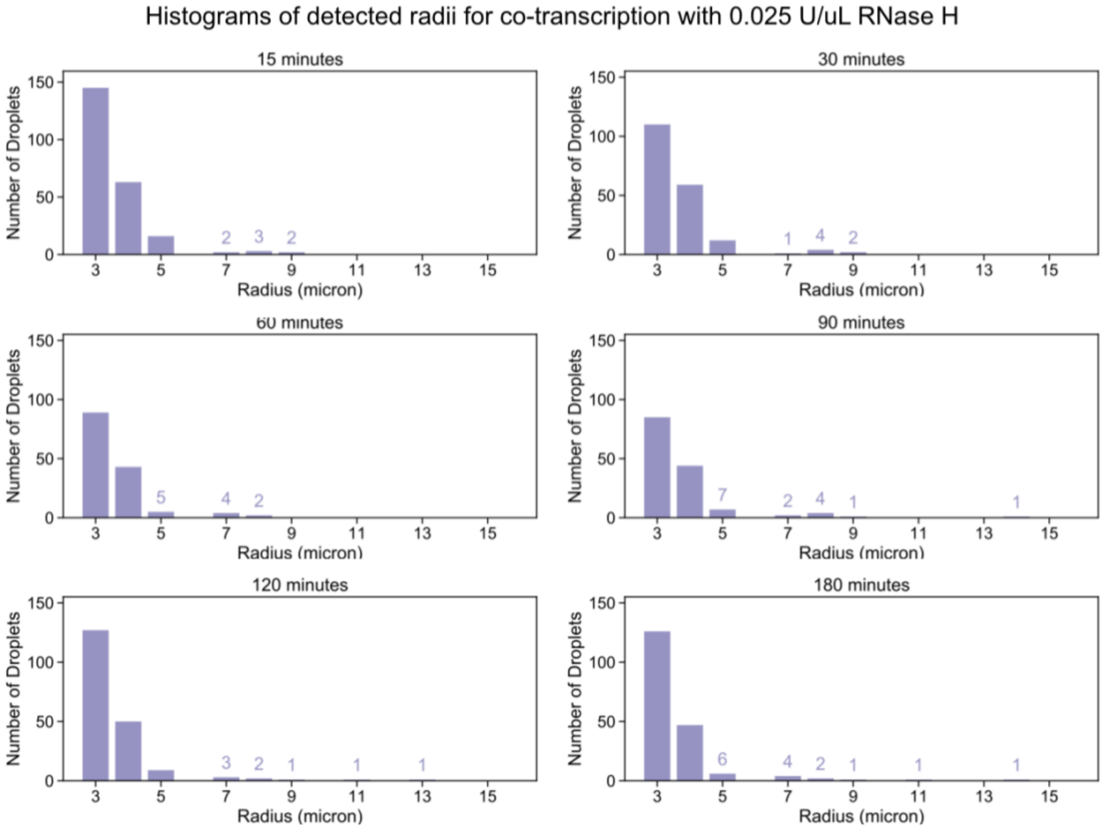

Figure S34: Histograms of the radii of detected droplets for the DNA-RNA hybrid nanotubes with co-transcription of RNA trigger from 100 nM template/gene and 0.025 U/µL RNase H in water-in-oil droplets experiment (Figure 6). Droplets were detected with the script described in SI Section 4.13.

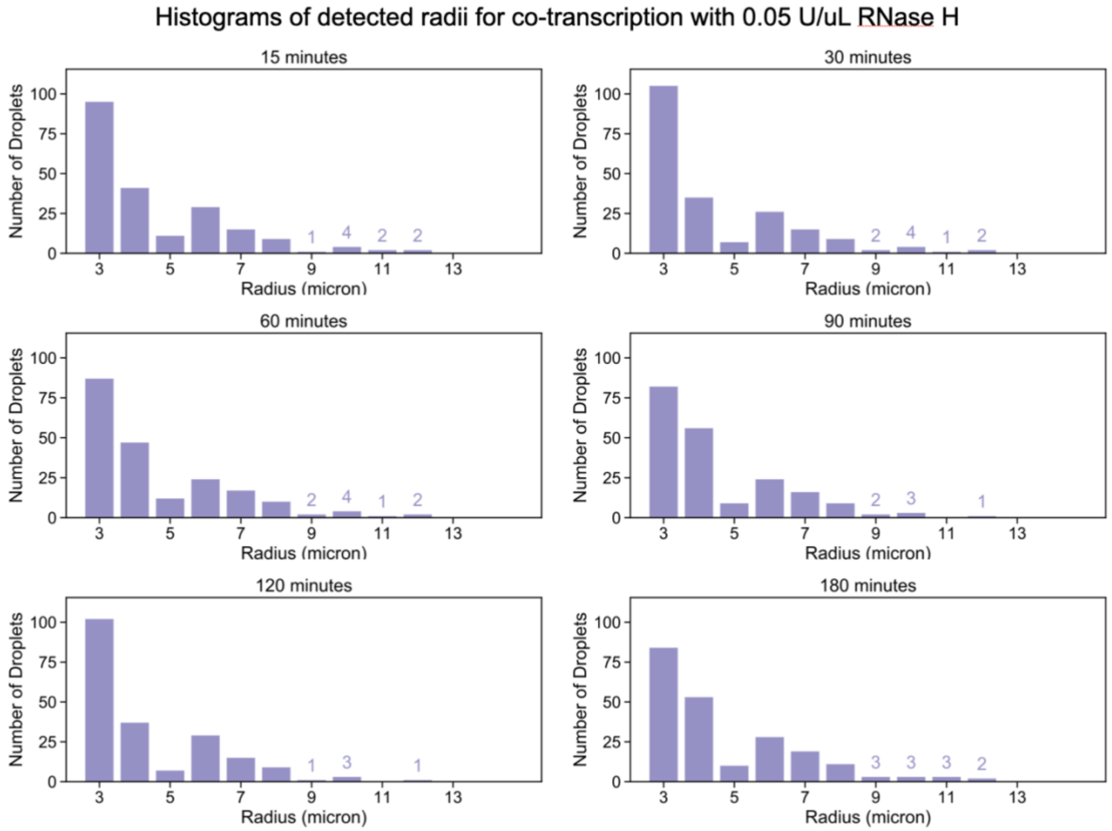

Figure S35: Histograms of the radii of detected droplets for the DNA-RNA hybrid nanotubes with co-transcription of RNA trigger from 100 nM template/gene and 0.05 U/μL RNase H in water-in-oil droplets experiment (Figure 6). Droplets were detected with the script described in SI Section 4.13.

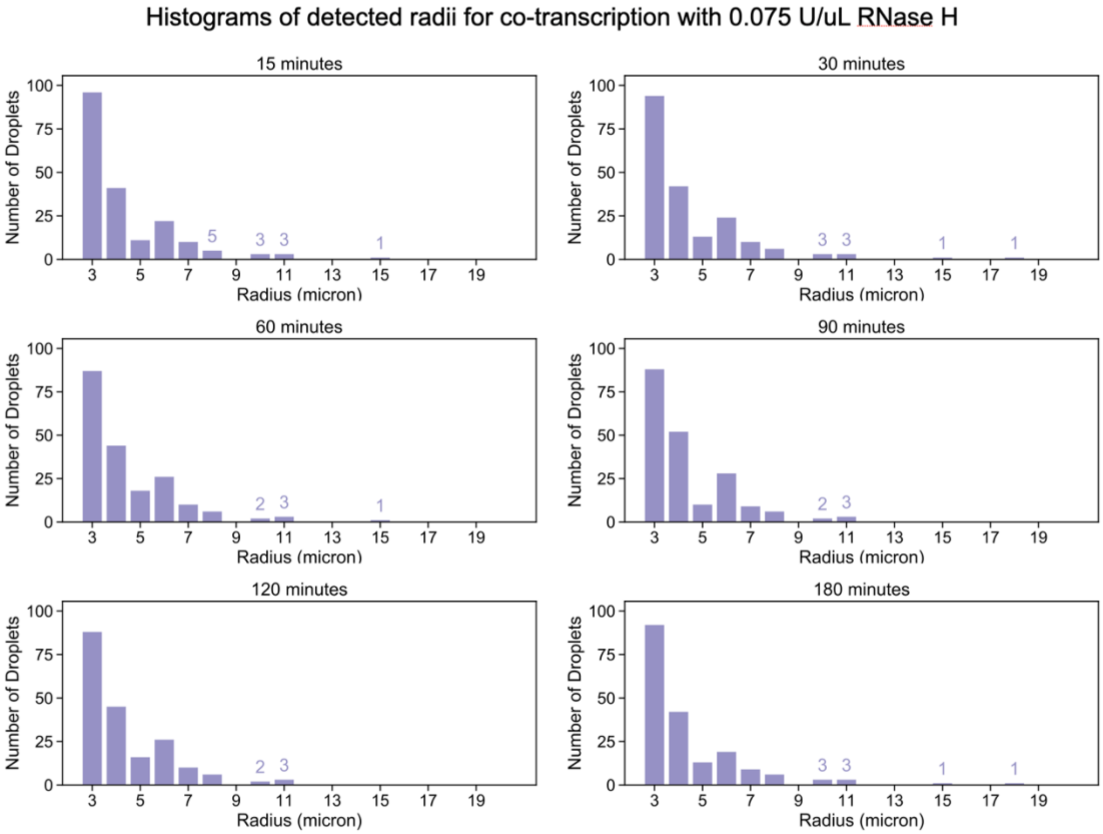

Figure S36: Histograms of the radii of detected droplets for the DNA-RNA hybrid nanotubes with co-transcription of RNA trigger from 100 nM template/gene and 0.075 U/µL RNase H in water-in-oil droplets experiment (Figure 6). Droplets were detected with the script described in SI Section 4.13.

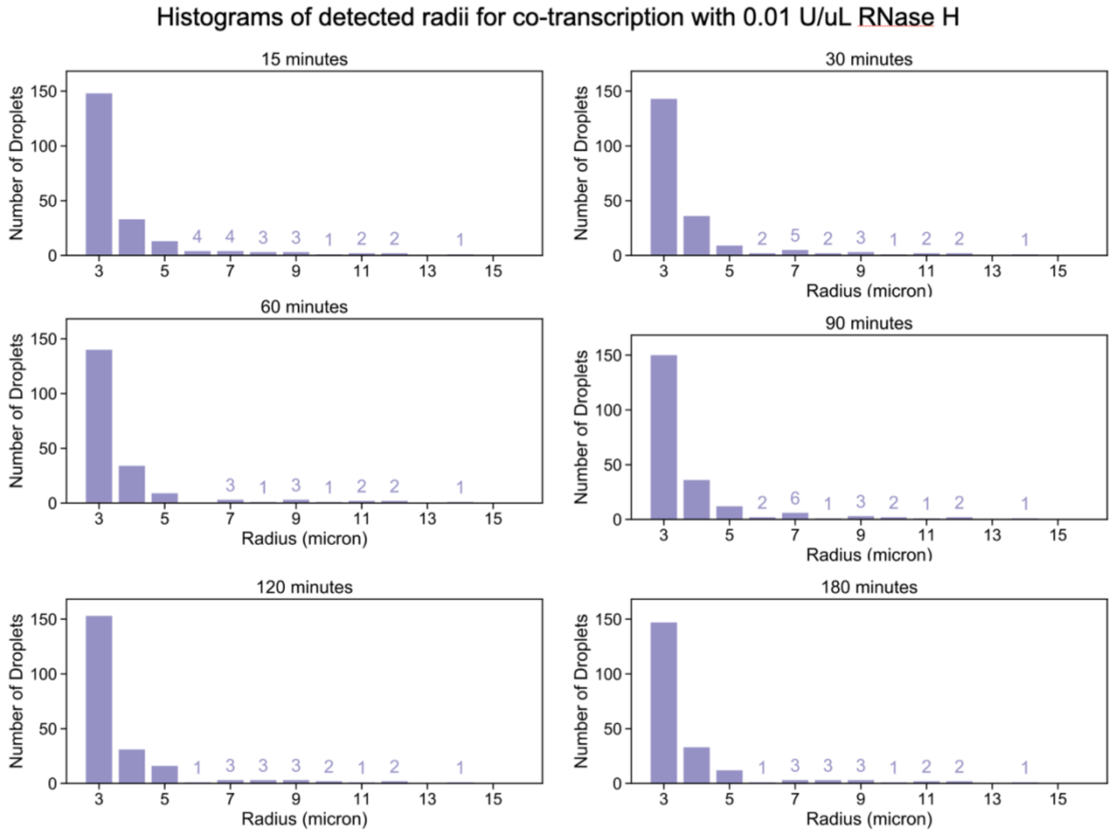

Figure S37: Histograms of the radii of detected droplets for the DNA-RNA hybrid nanotubes with co-transcription of RNA trigger from 100 nM template/gene and 0.01 U/µL RNase H in water-in-oil droplets experiment (Figure 6). Droplets were detected with the script described in SI Section 4.13.

S5.11 Droplet radius versus skewness and kurtosis

In this section we plot the skewness and kurtosis measured for each droplet of a given radius. The plots are organized to cover all experimental assays reported in the main text, and each plot corresponds to images collected at a specific time during the experiment, which is reported in the title.

In general, for a given time at which the droplets are imaged, we do not observe a correlation between droplet radius and measured skewness and kurtosis in any of the experiments. We report the mean skewness and kurtosis of droplets in a given radius bin as a guide to the eye. In some cases, a single droplet of a given radius is present, and although it is an outlier the data point was not discarded.

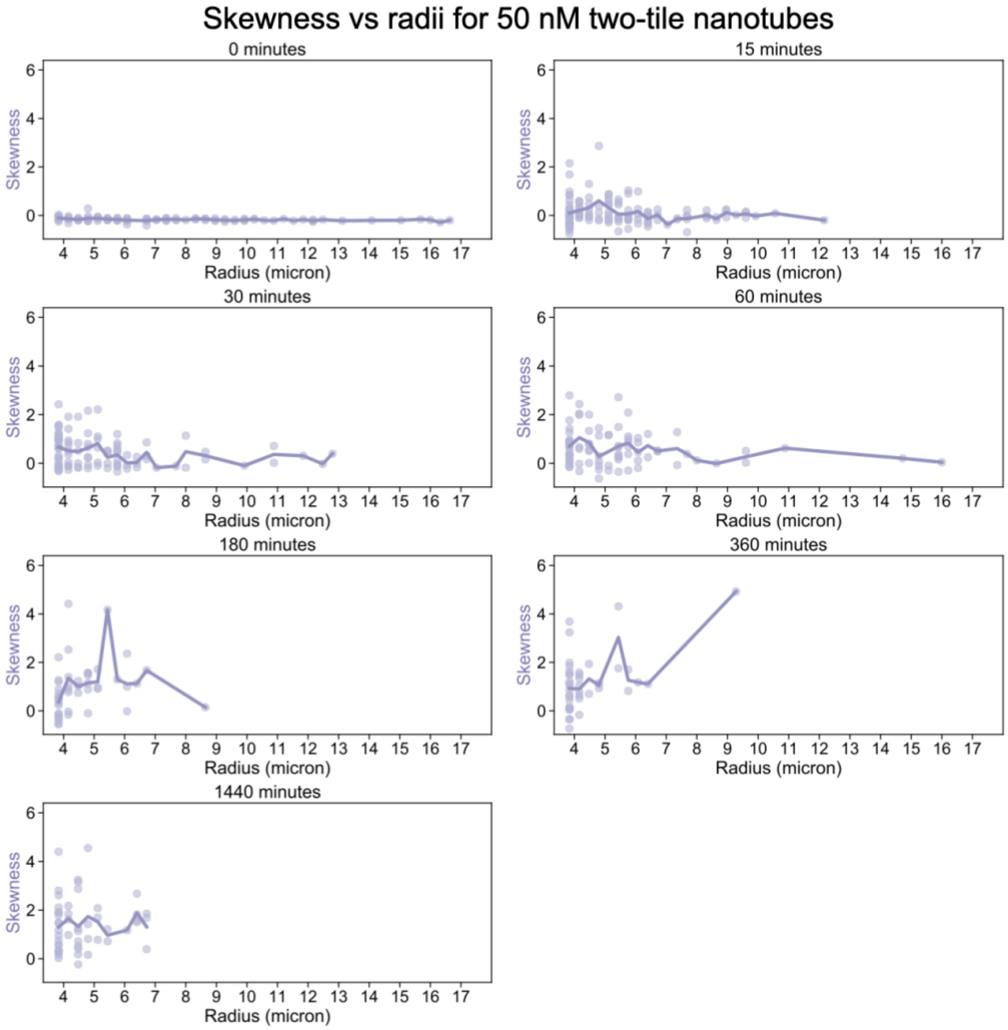

Figure S38: Skewness for the 50 nM two-tile nanotubes in water-in-oil droplets experiment (Figure 3 of the main text).

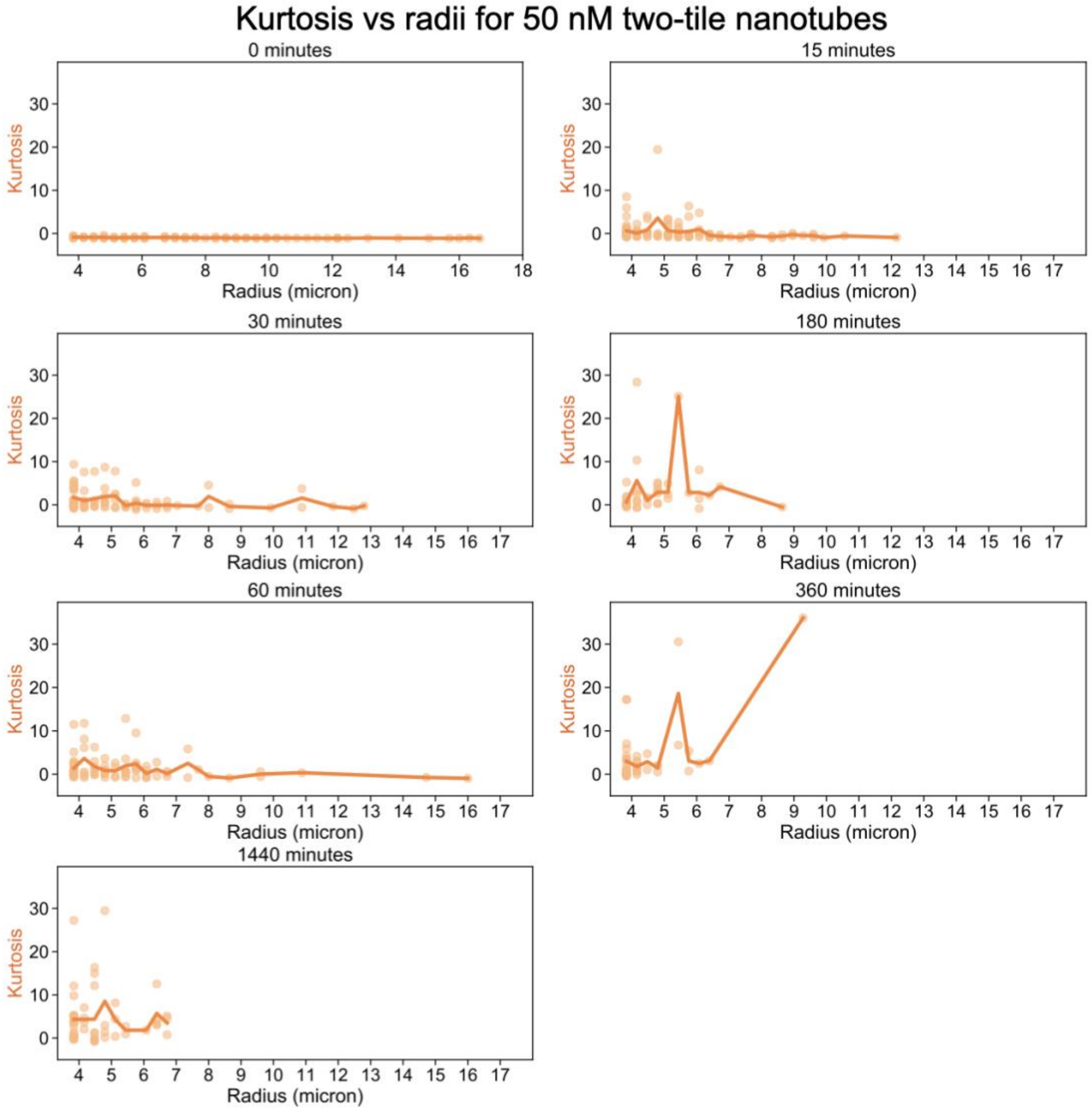

Figure S39: Kurtosis for the 50 nM two-tile nanotubes in water-in-oil droplets experiment (Figure 3 of the main text).

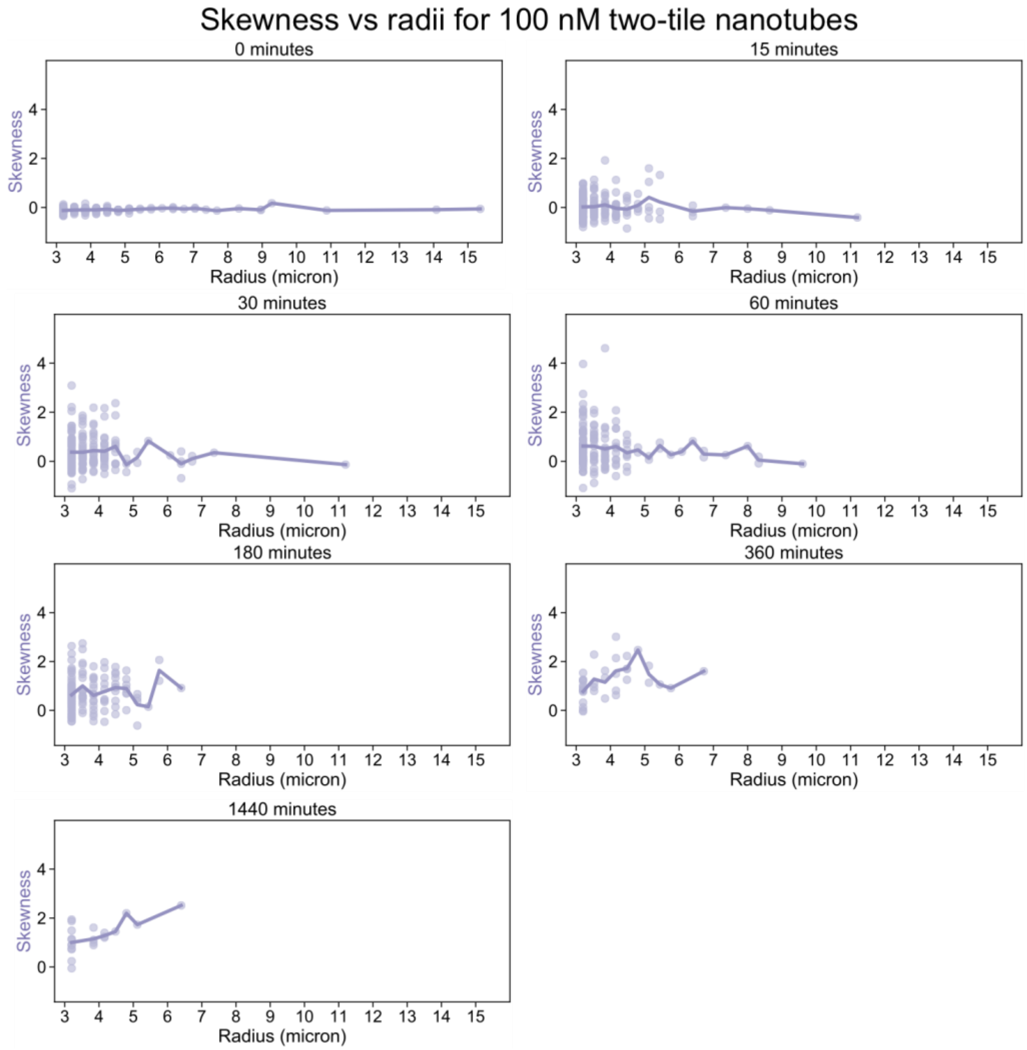

Figure S40: Skewness for the 100 nM two-tile nanotubes in water-in-oil droplets experiment (Figure 3 of the main text).

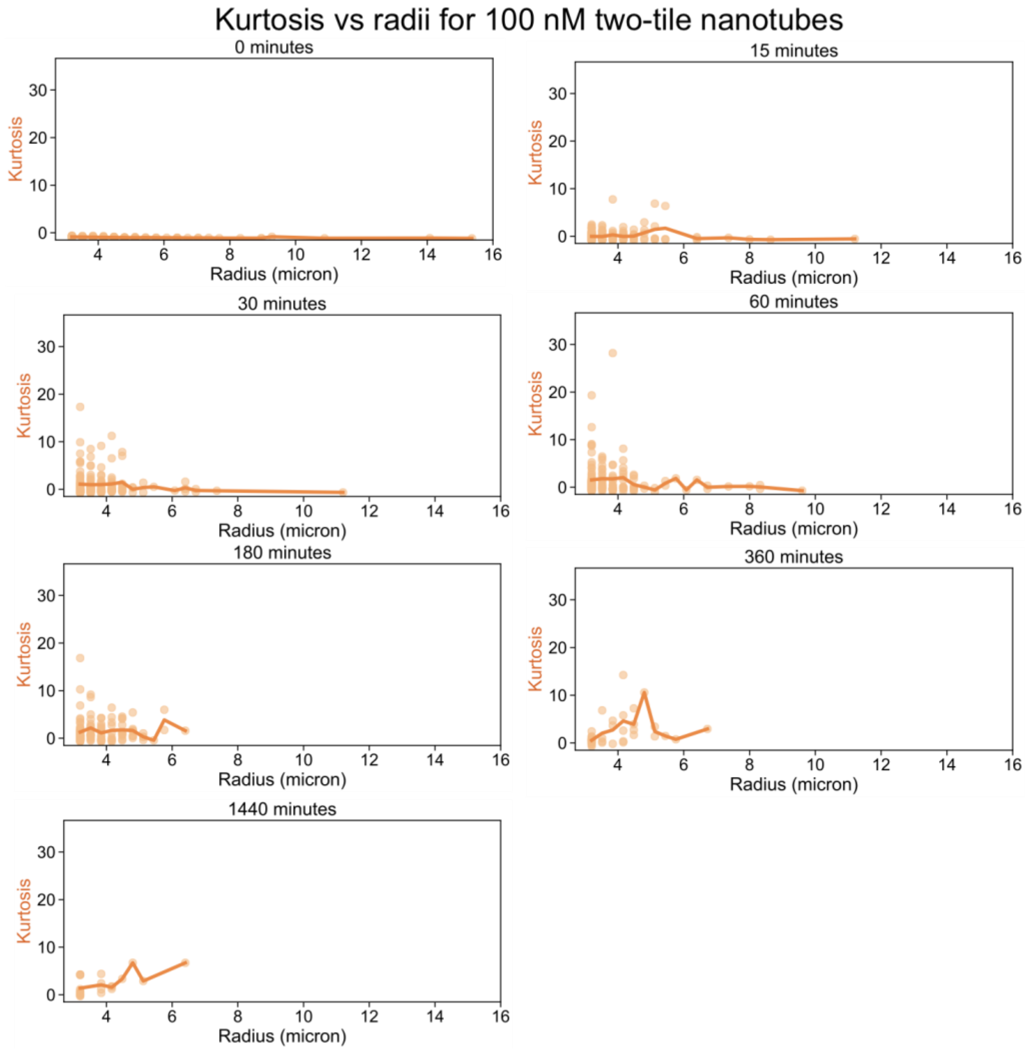

Figure S41: Kurtosis for the 100 nM two-tile nanotubes in water-in-oil droplets experiment (Figure 3 of the main text).

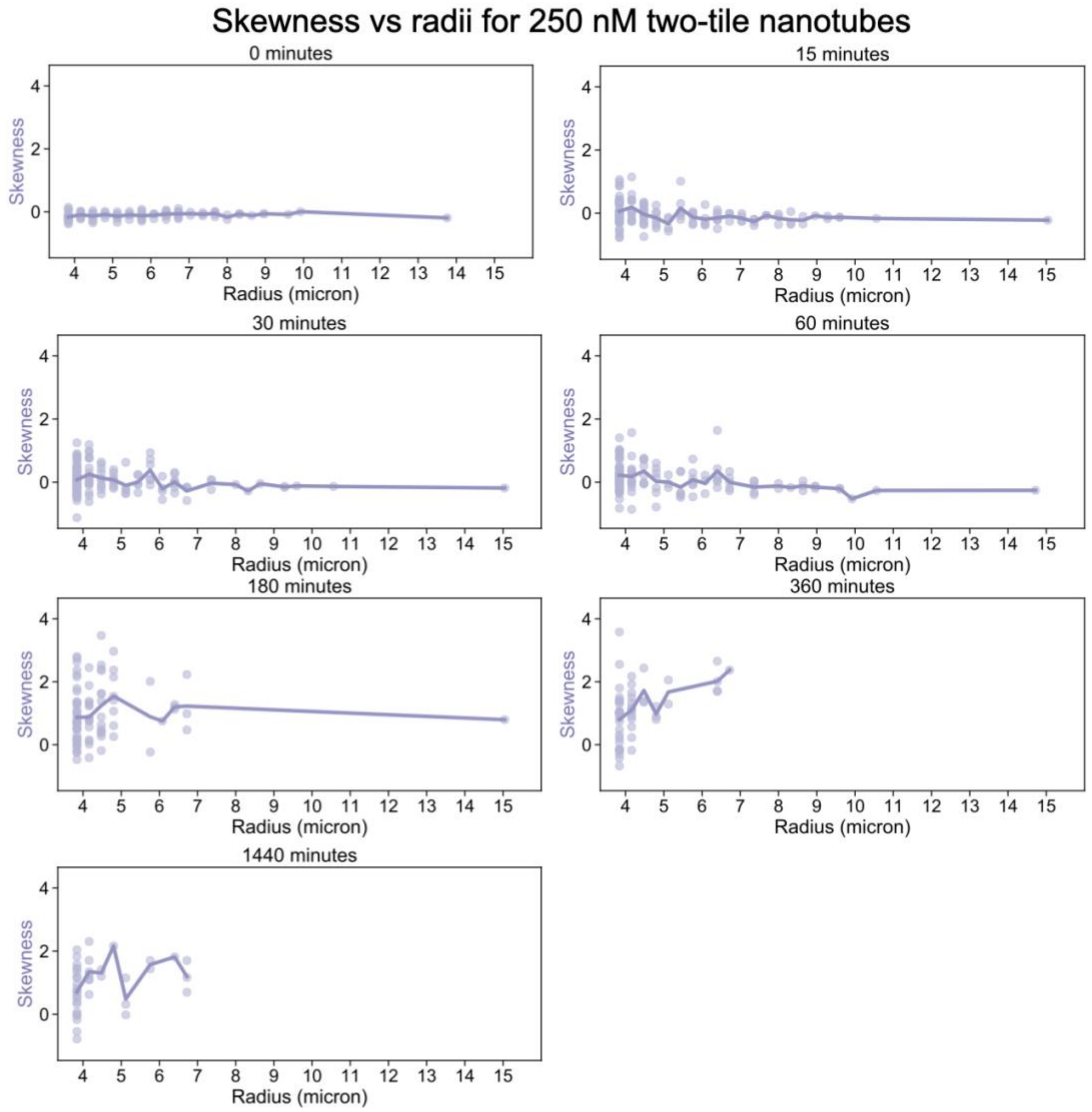

Figure S42: Skewness for the 250 nM two-tile nanotubes in water-in-oil droplets experiment (Figure 3 of the main text).

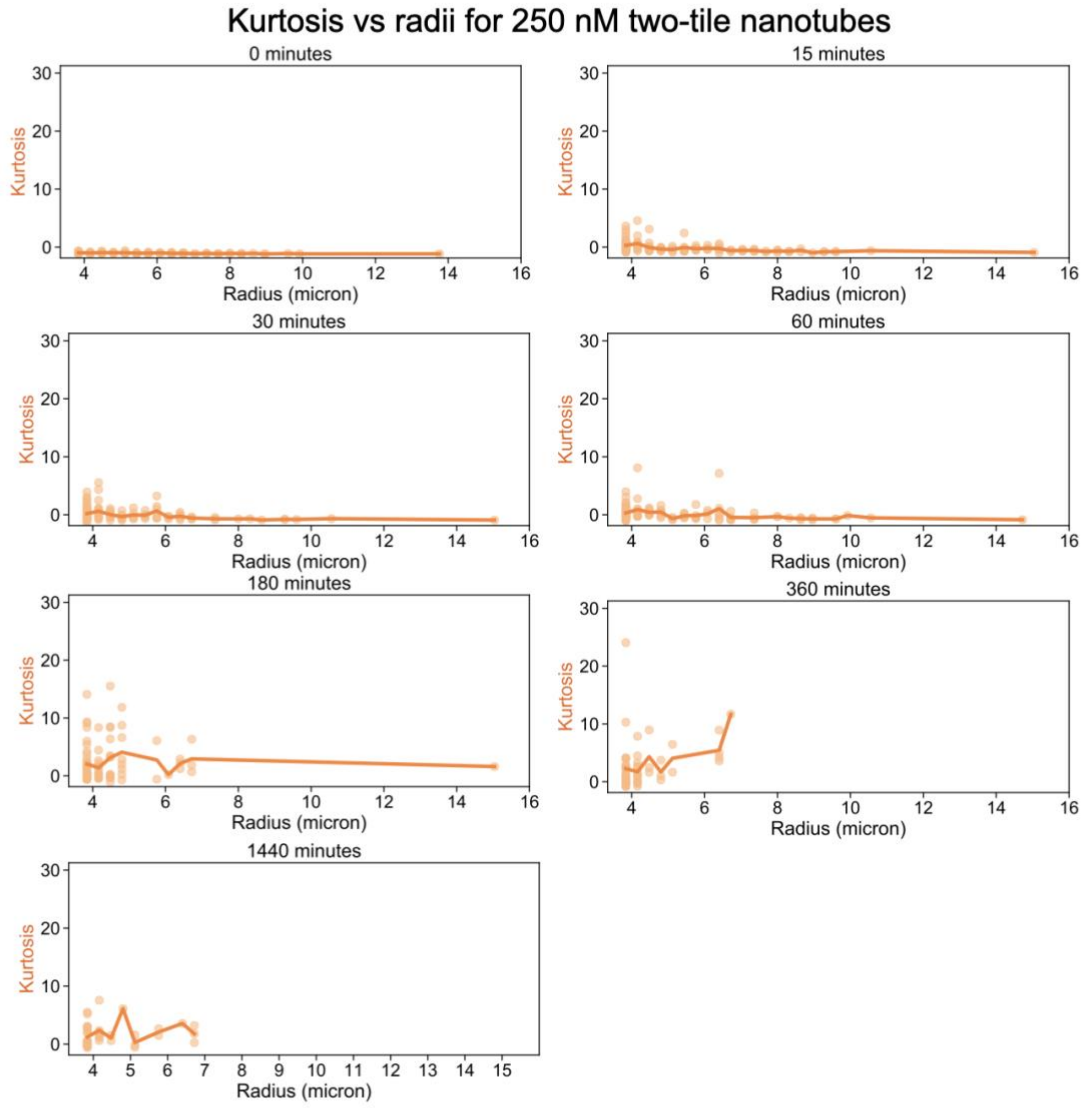

Figure S43: Kurtosis for the 250 nM two-tile nanotubes in water-in-oil droplets experiment (Figure 3 of the main text).

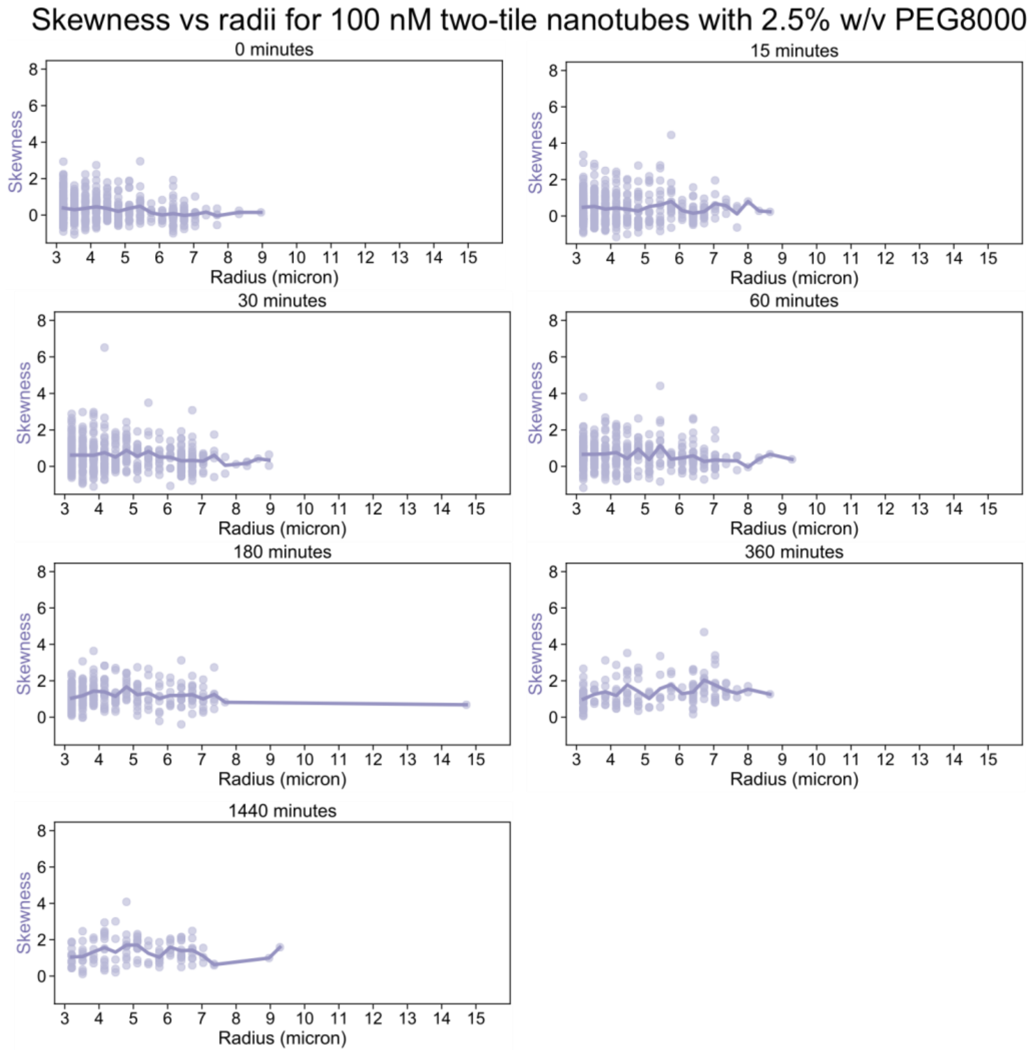

Figure S44: Skewness for the 100 nM two-tile nanotubes with 2.5%w/v PEG8000 in water-in-oil droplets experiment (Figure 3 of the main text).

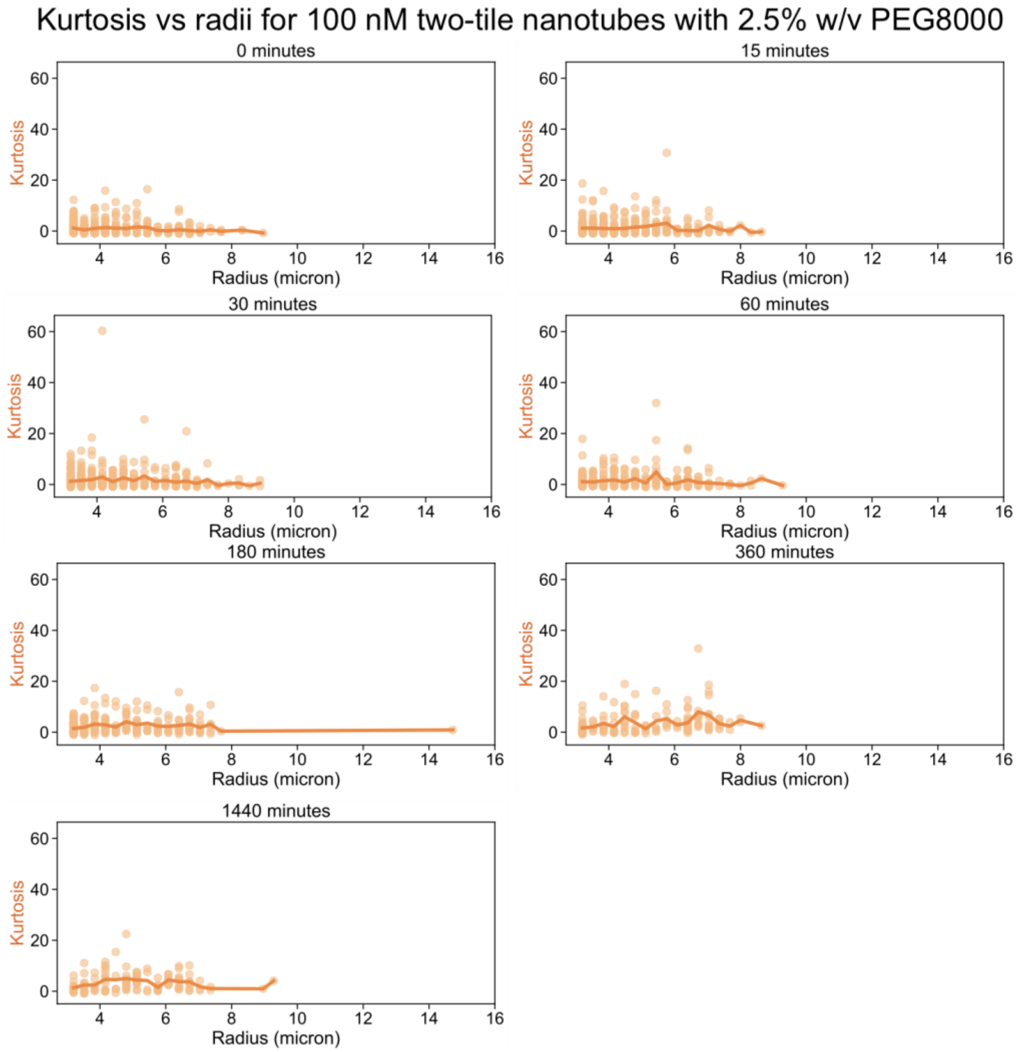

Figure S45: Kurtosis for the 100 nM two-tile nanotubes with 2.5%w/v PEG8000 in water-in-oil droplets experiment (Figure 3 of the main text).

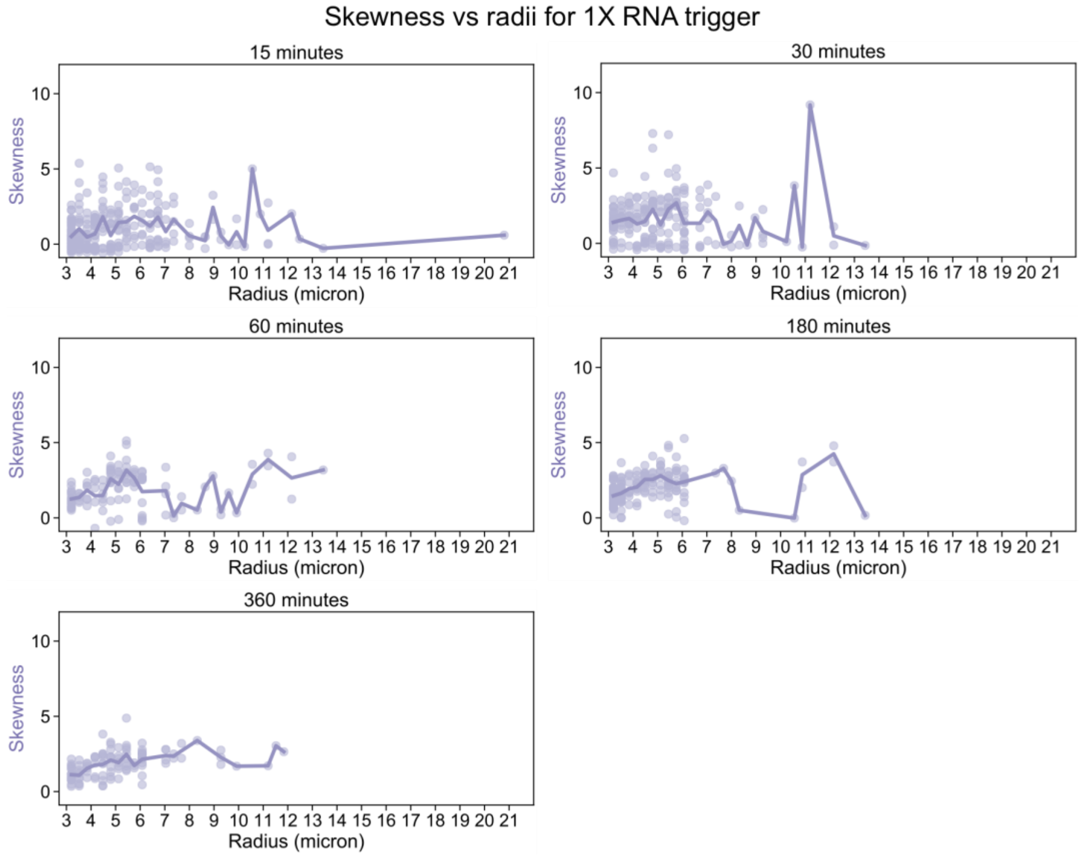

Figure S46: Skewness for droplets of different radii detected in the 1x gel-extracted RNA trigger in water-in-oil droplets experiment (Figure 4 of the main text).

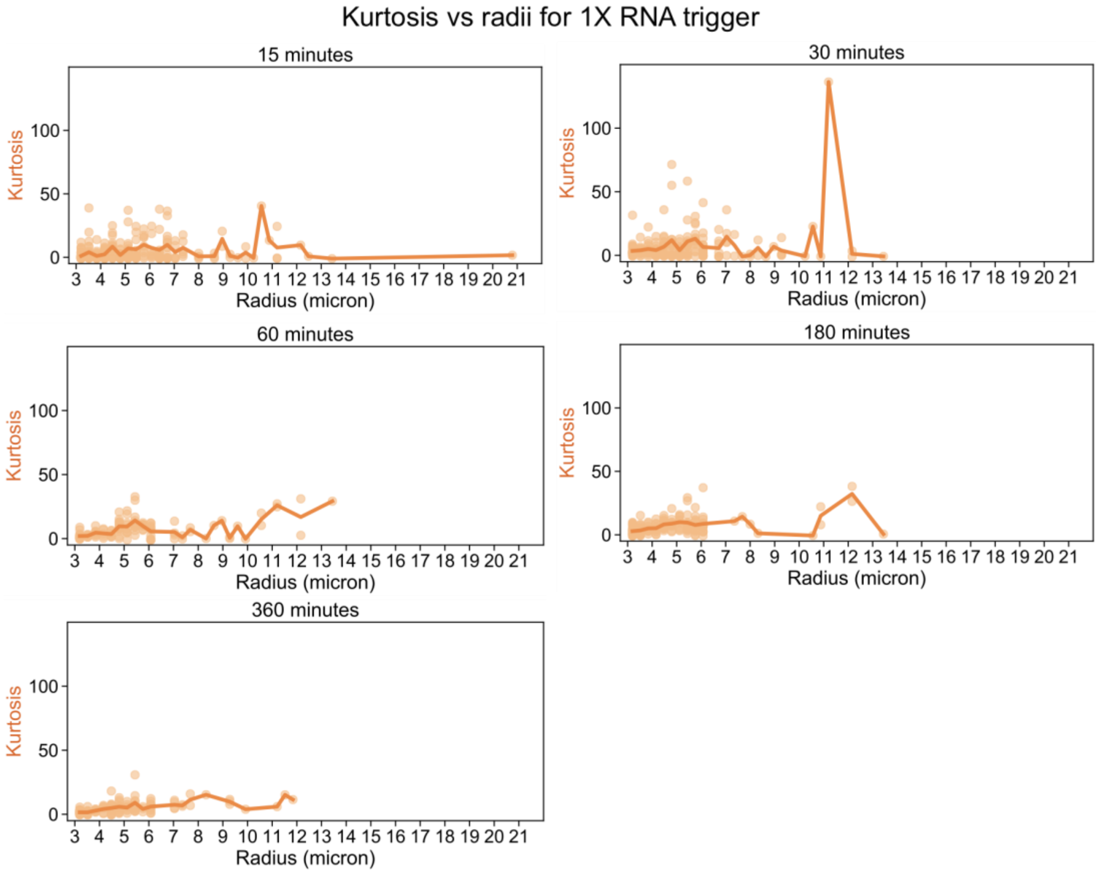

Figure S47: Kurtosis for droplets of different radii detected in the 1x gel-extracted RNA trigger in water-in-oil droplets experiment (Figure 4 of the main text).

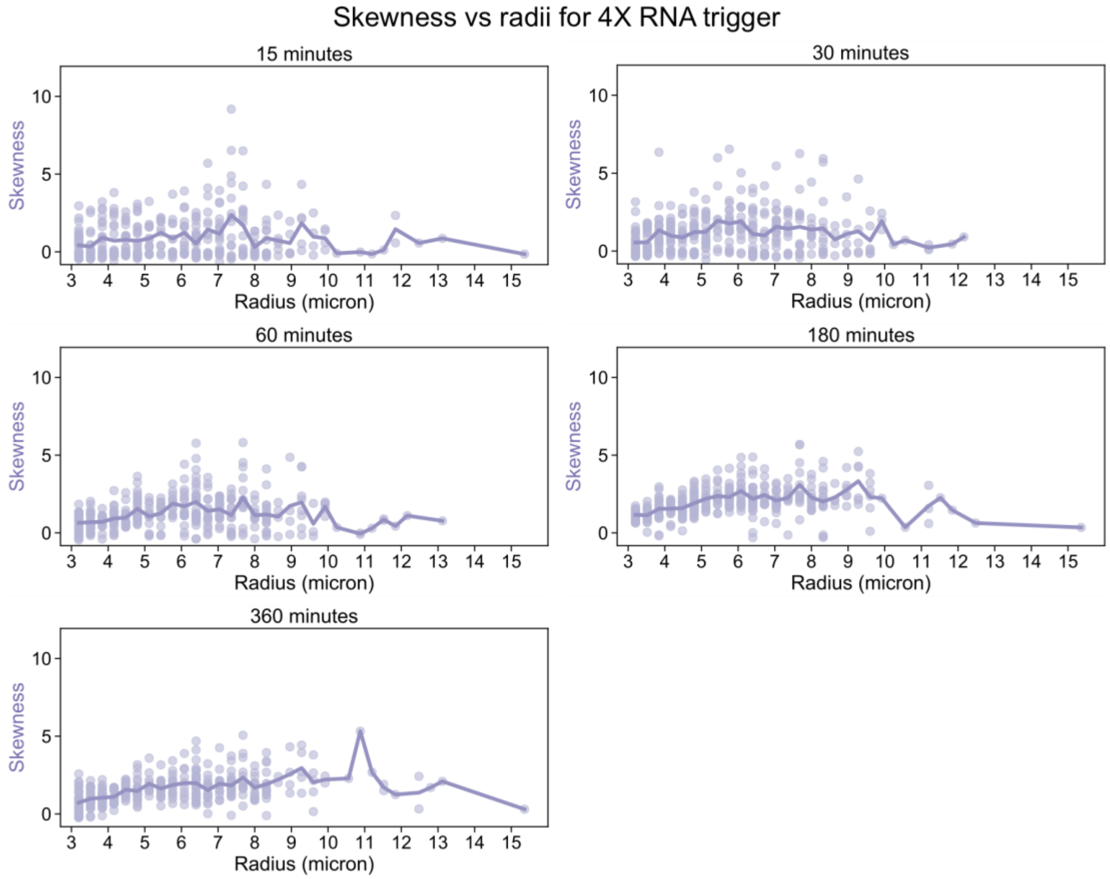

Figure S48: Skewness for droplets of different radii detected in the 4x gel-extracted RNA trigger in water-in-oil droplets experiment (Figure 4 of the main text).

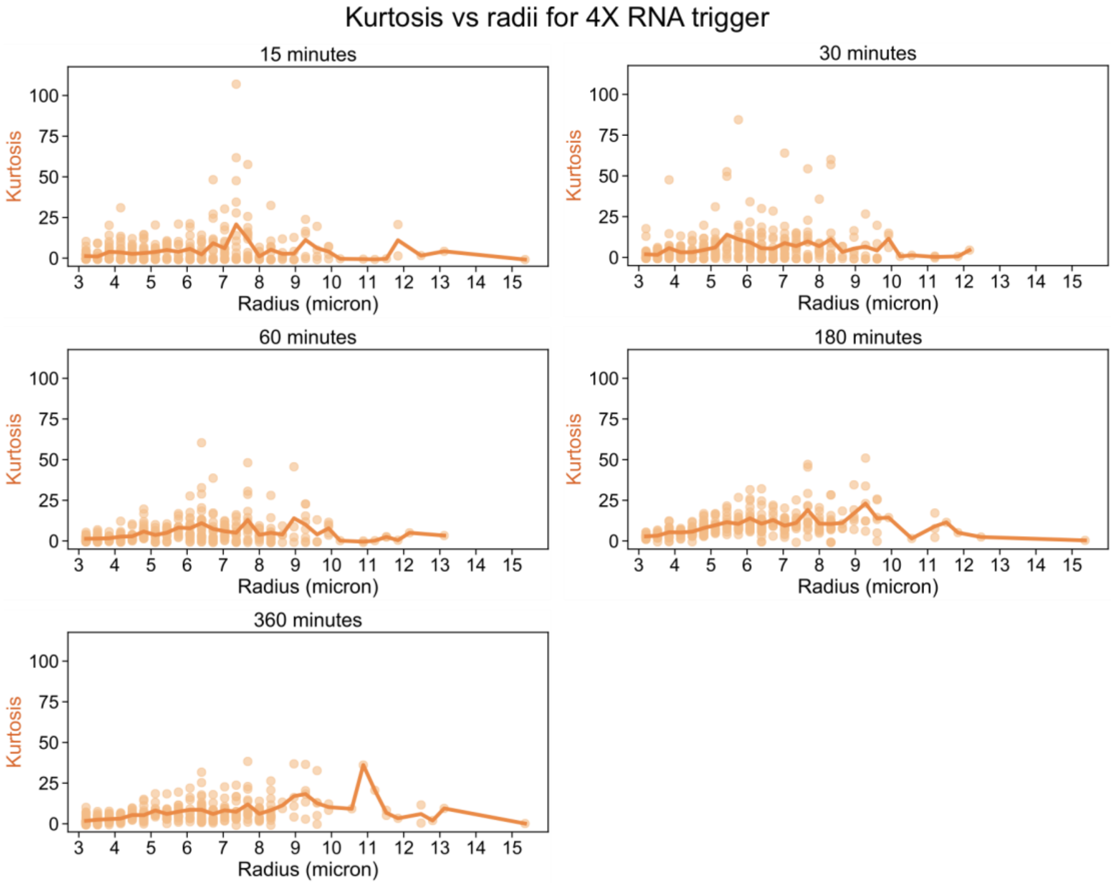

Figure S49: Kurtosis for droplets of different radii detected in the 4x gel-extracted RNA trigger in water-in-oil droplets experiment (Figure 4 of the main text).

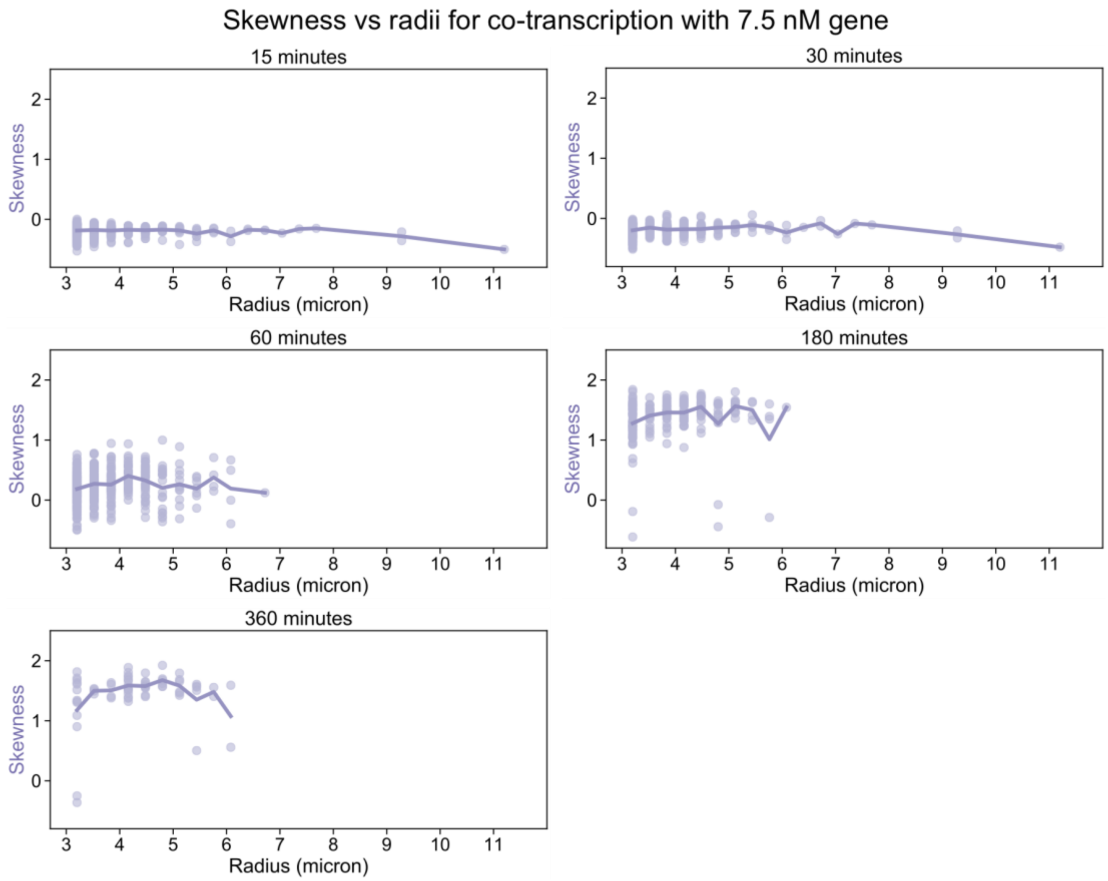

Figure S50: Skewness of droplets for the DNA-RNA hybrid nanotubes with co-transcription of RNA trigger from 7.5 nM template/gene in water-in-oil droplets experiment (Figure 5 of the main text).

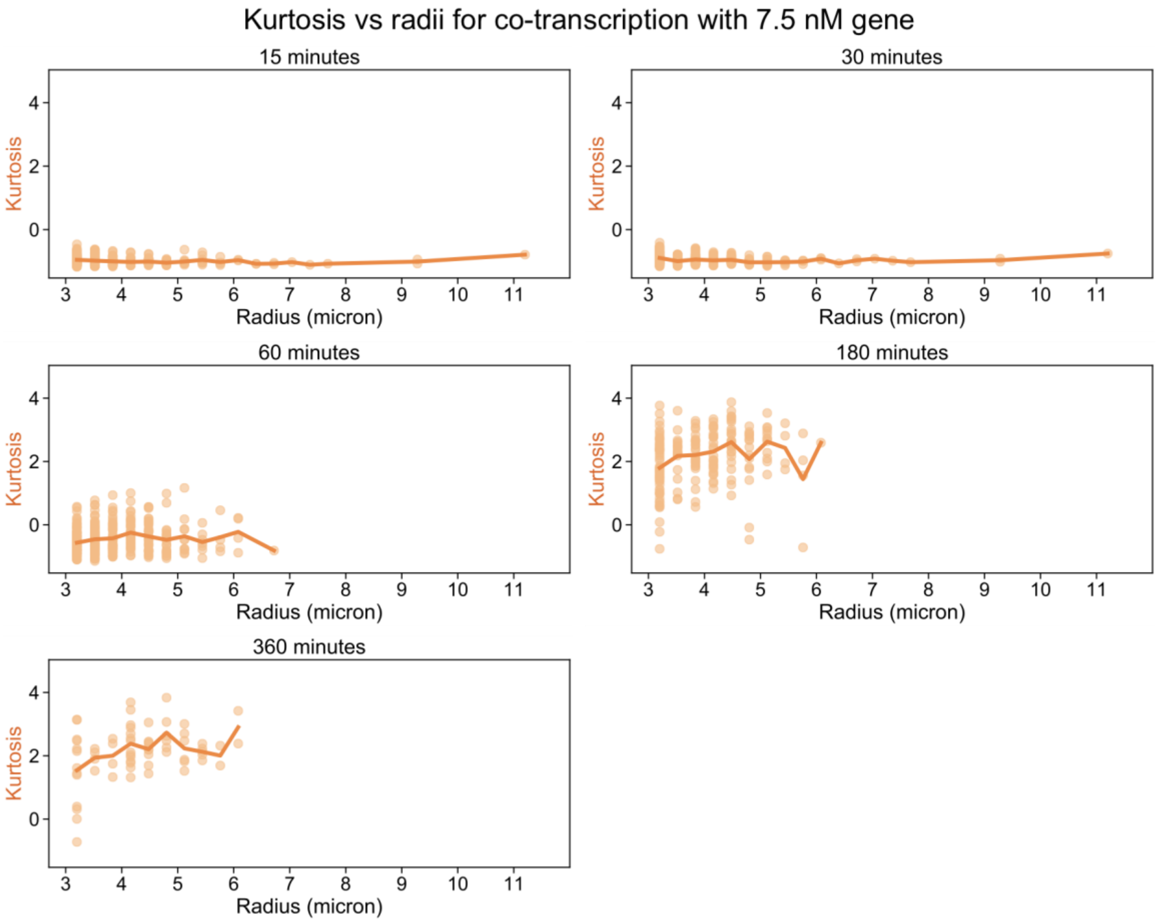

Figure S51: Kurtosis of droplets for the DNA-RNA hybrid nanotubes with co-transcription of RNA trigger from 7.5 nM template/gene in water-in-oil droplets experiment (Figure 5 of the main text).

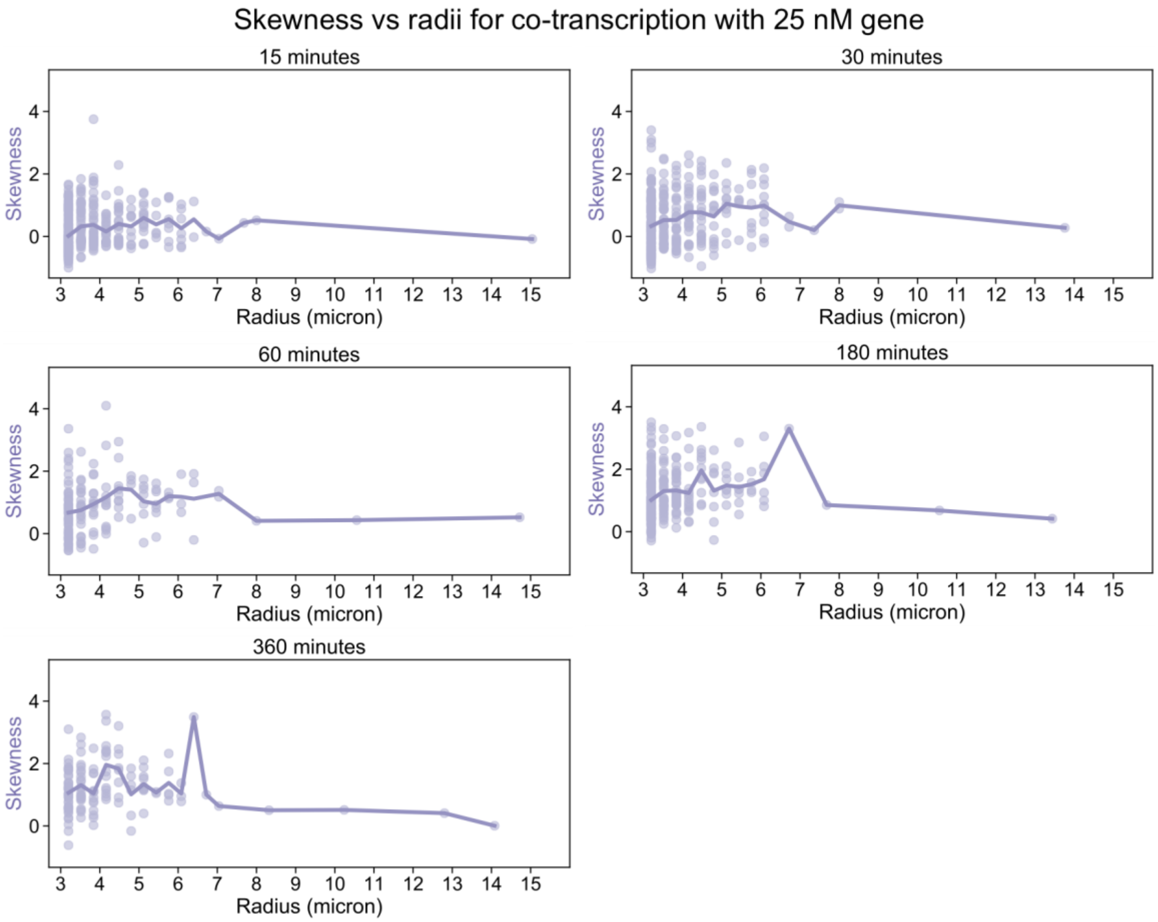

Figure S52: Skewness of droplets for the DNA-RNA hybrid nanotubes with co-transcription of RNA trigger from 25 nM template/gene in water-in-oil droplets experiment (Figure 5 of the main text).

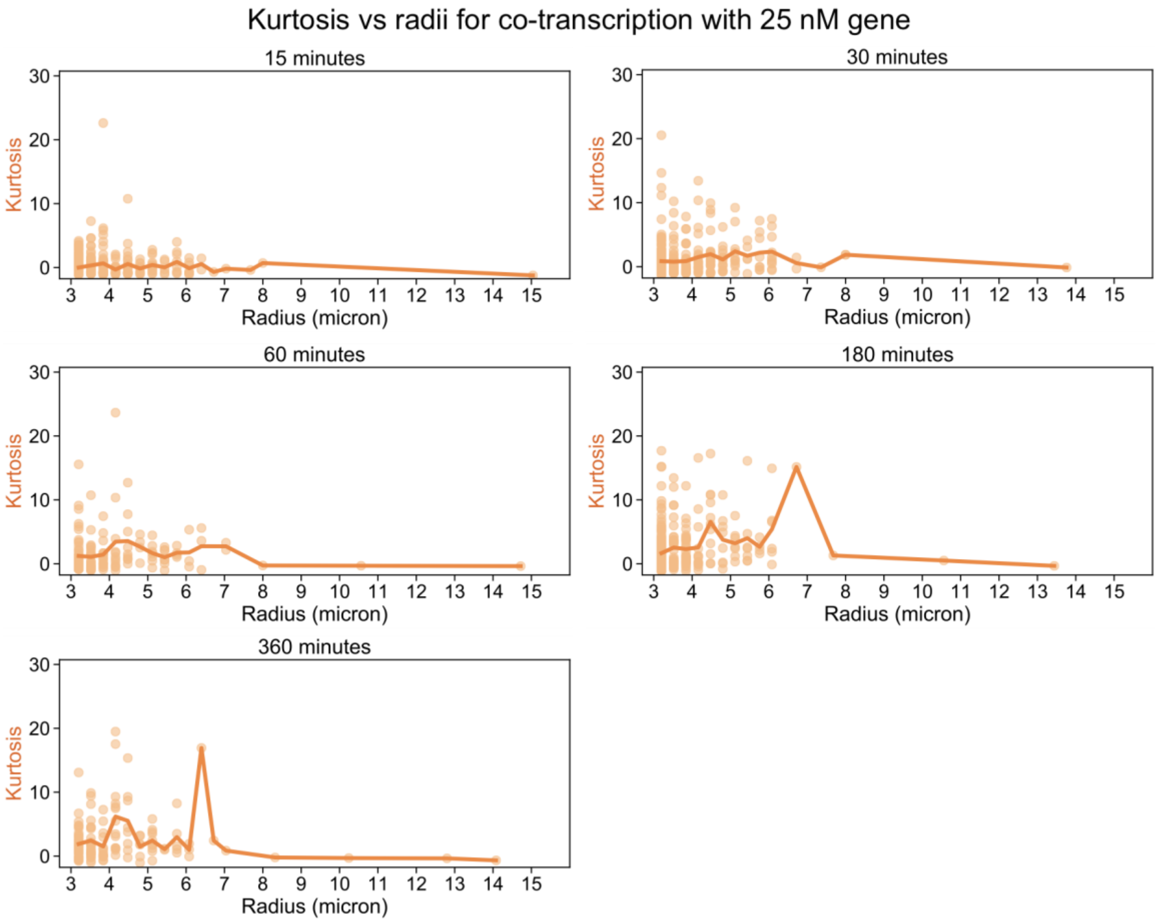

Figure S53: Kurtosis of droplets for the DNA-RNA hybrid nanotubes with co-transcription of RNA trigger from 25 nM template/gene in water-in-oil droplets experiment (Figure 5 o the main text).

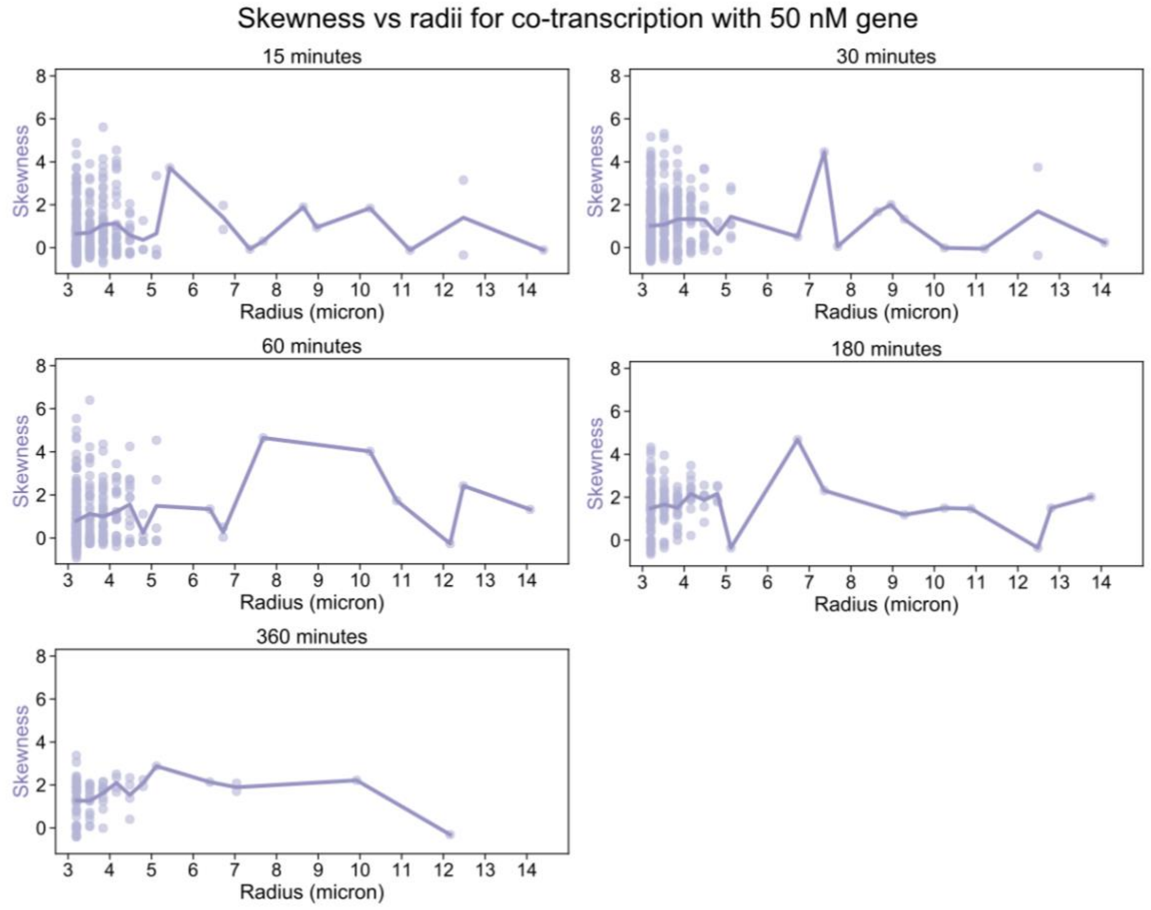

Figure S54: Skewness of droplets for the DNA-RNA hybrid nanotubes with co-transcription of RNA trigger from 50 nM template/gene in water-in-oil droplets experiment (Figure 5 of the main text).

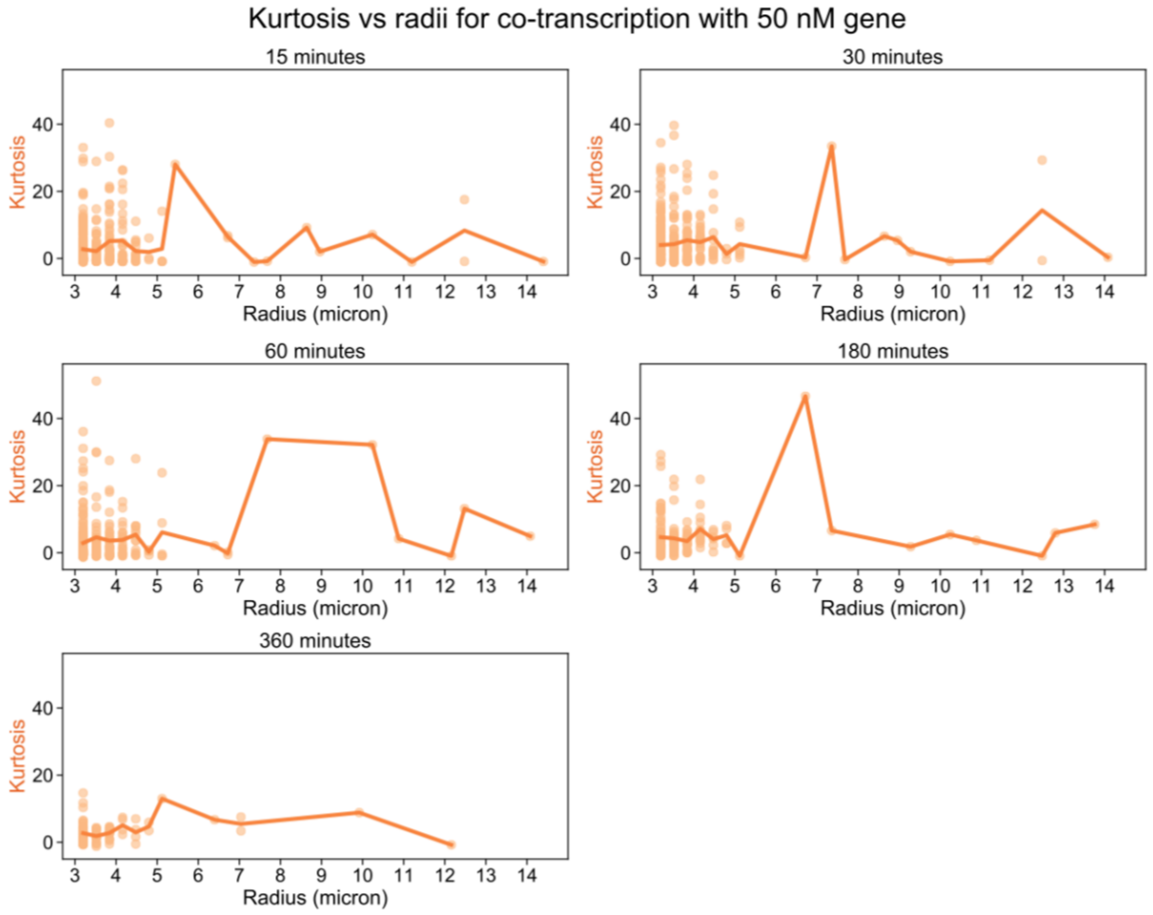

Figure S55. Kurtosis of droplets for the DNA-RNA hybrid nanotubes with co-transcription of RNA trigger from 50 nM template/gene in water-in-oil droplets experiment (Figure 5 of the main text).

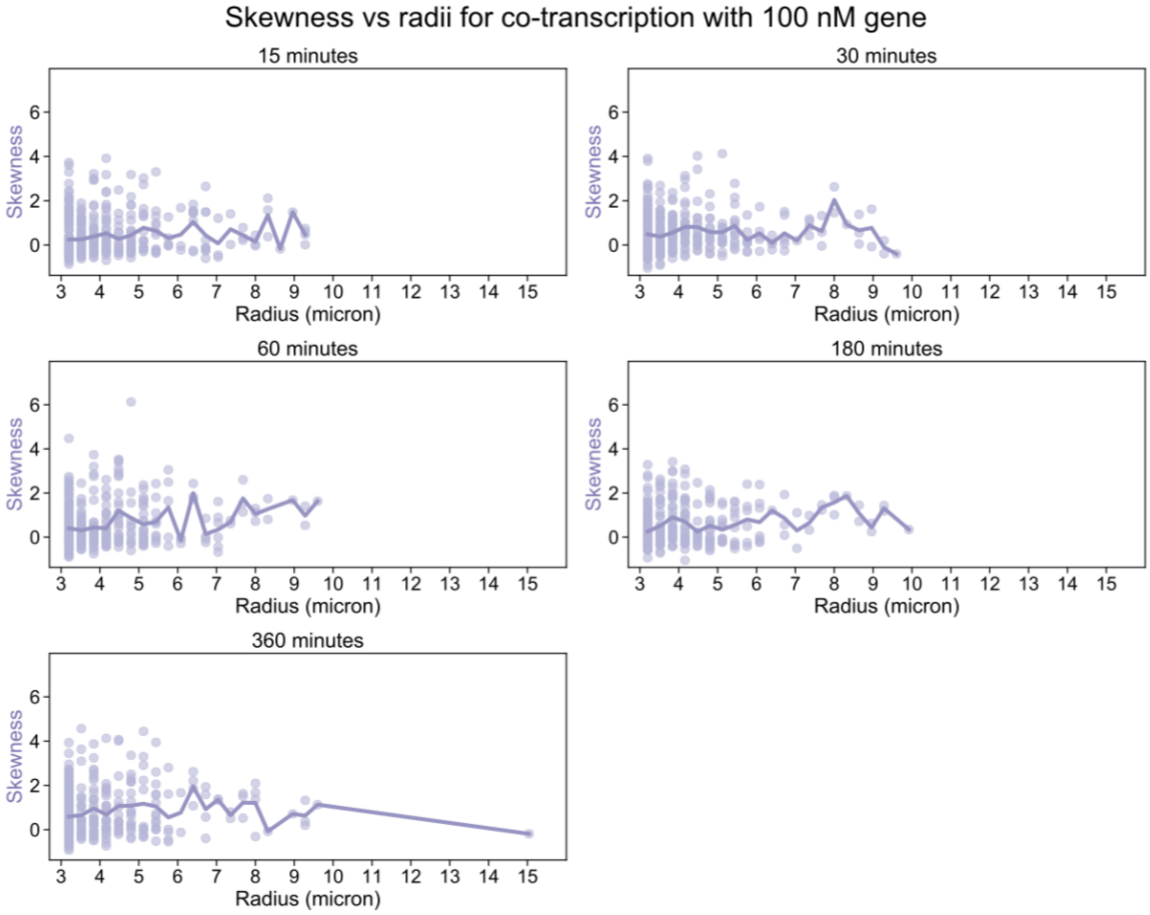

Figure S56: Skewness of droplets for the DNA-RNA hybrid nanotubes with co-transcription of RNA trigger from 100 nM template/gene in water-in-oil droplets experiment (Figure 5 of the main text).

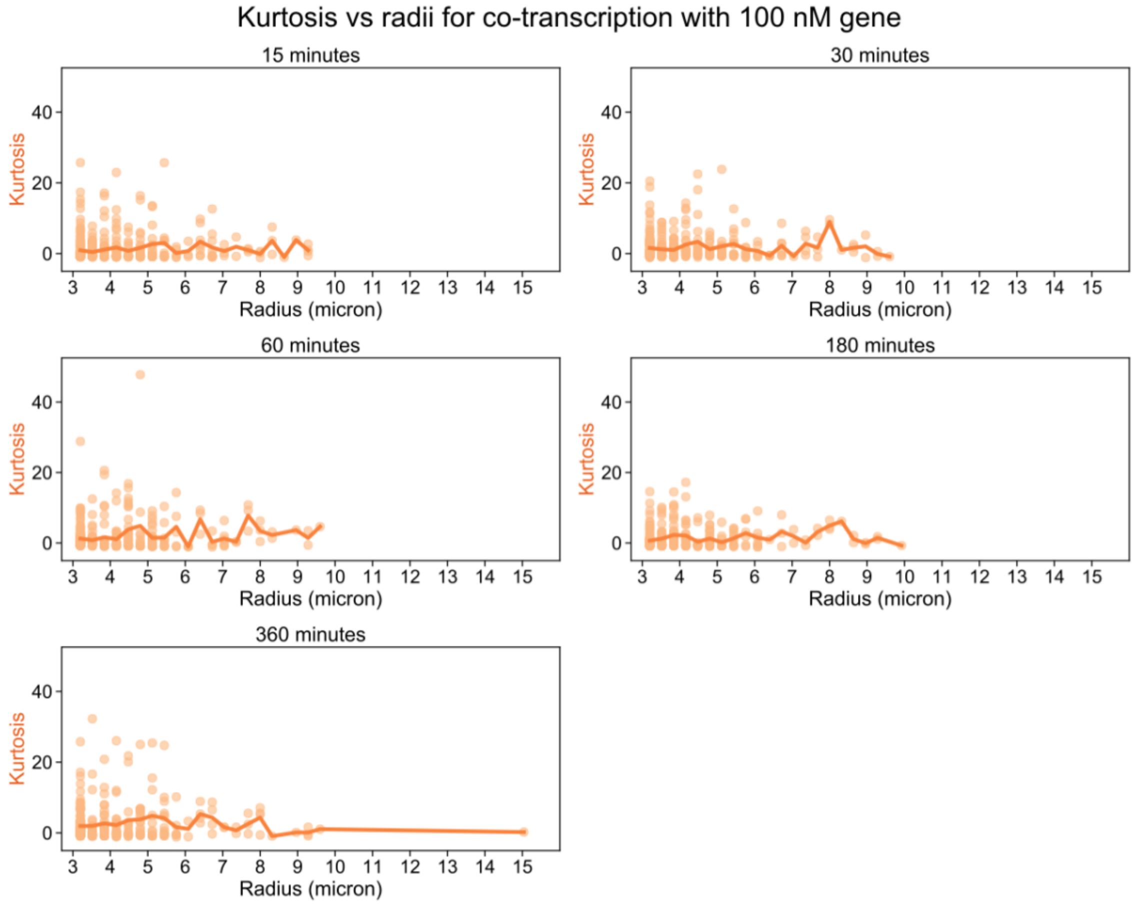

Figure S57: Kurtosis of droplets for the DNA-RNA hybrid nanotubes with co-transcription of RNA trigger from 100 nM template/gene in water-in-oil droplets experiment (Figure 5 of the main text).

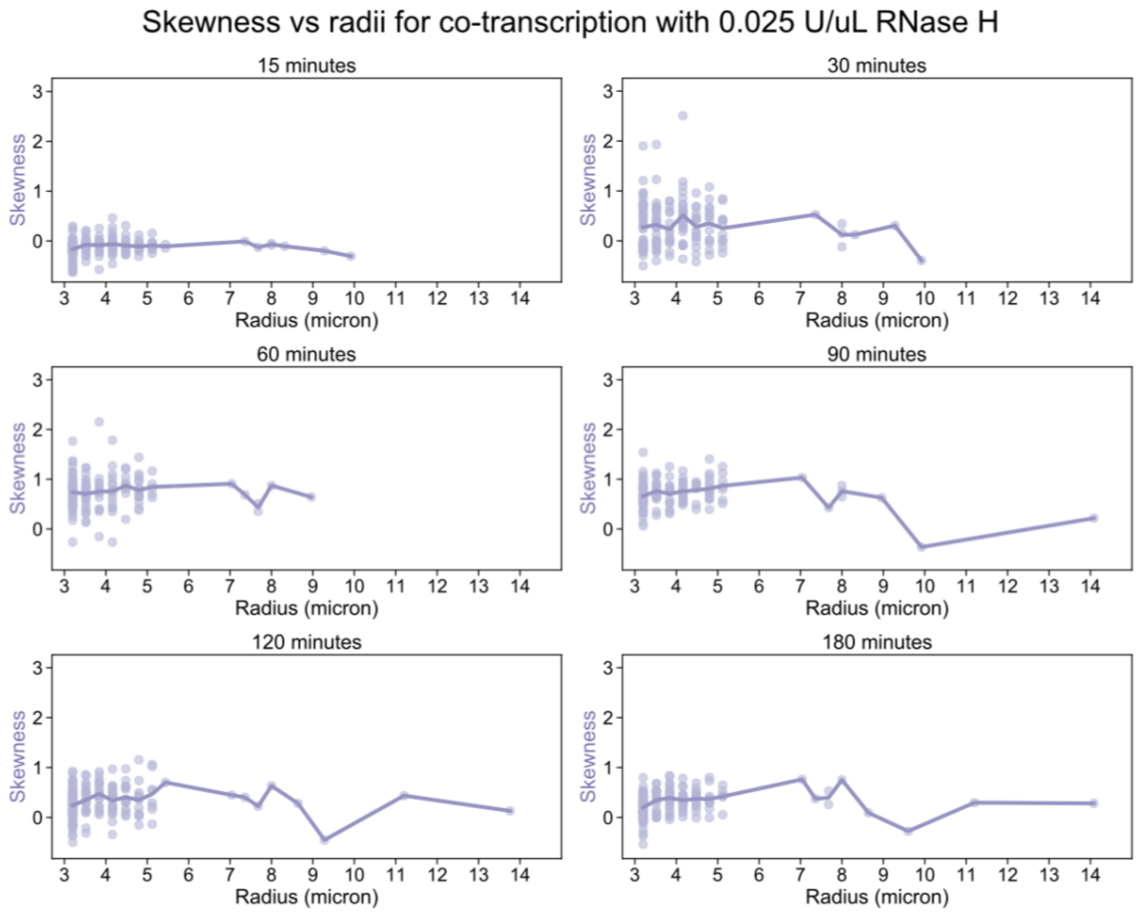

Figure S58: Skewness of droplets for the DNA-RNA hybrid nanotubes with co-transcription of RNA trigger from 100 nM template/gene and 0.025 U/µL RNase H in water-in-oil droplets experiment (Figure 6 of the main text).

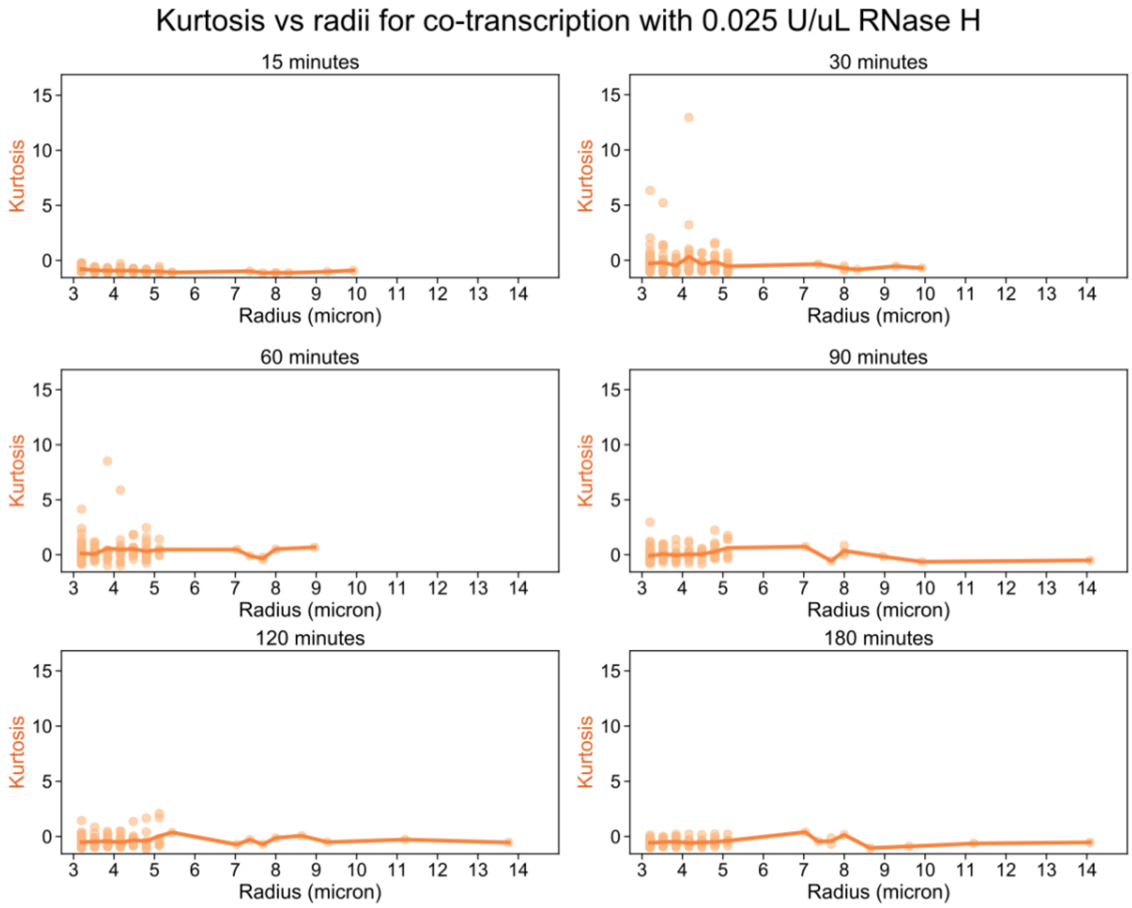

Figure S59: Kurtosis of droplets for the DNA-RNA hybrid nanotubes with co-transcription of RNA trigger from 100 nM template/gene and 0.025 U/µL RNase H in water-in-oil droplets experiment (Figure 6 of the main text).

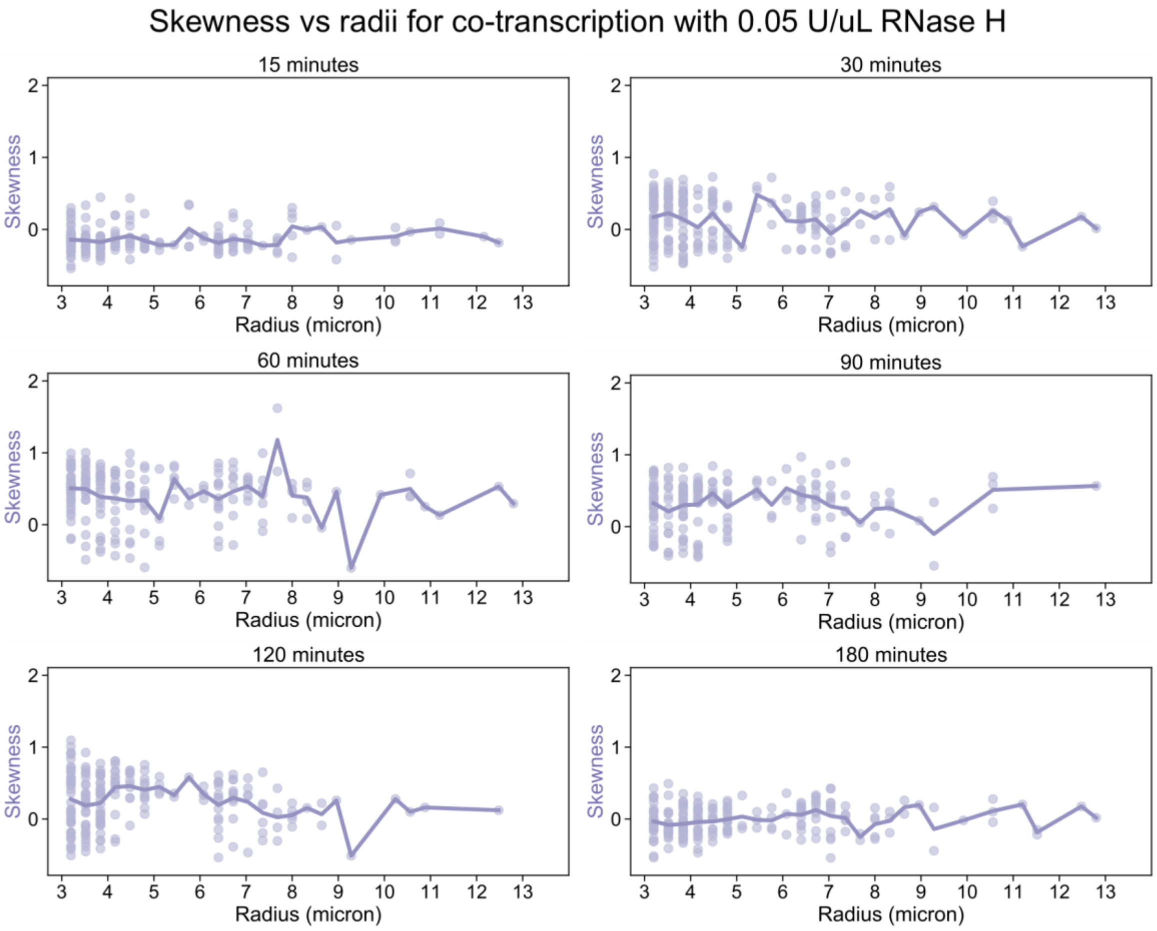

Figure S60: Skewness of droplets for the DNA-RNA hybrid nanotubes with co-transcription of RNA trigger from 100 nM template/gene and 0.05 U/µL RNase H in water-in-oil droplets experiment (Figure 6 of the main text).

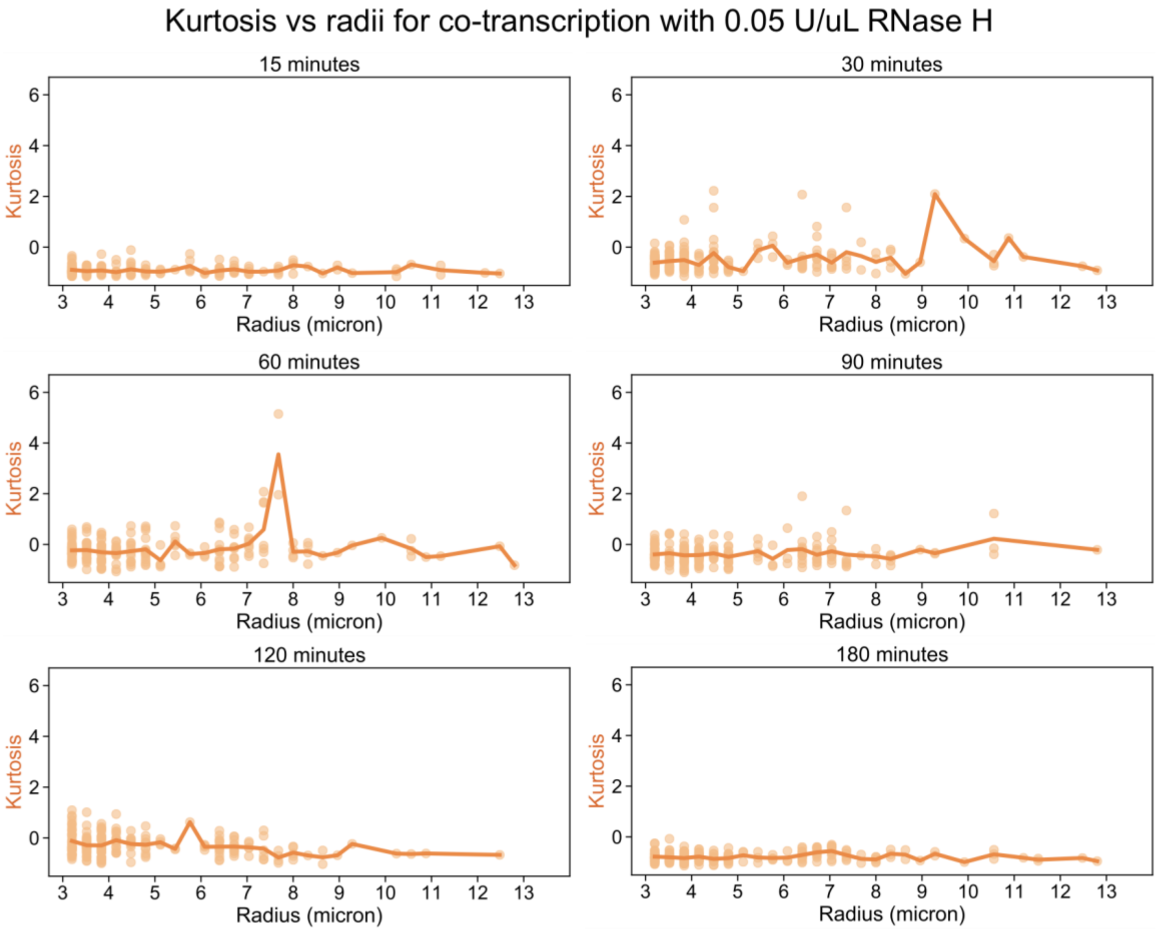

Figure S61: Kurtosis of droplets for the DNA-RNA hybrid nanotubes with co-transcription of RNA trigger from 100 nM template/gene and 0.05 U/µL RNase H in water-in-oil droplets experiment (Figure 6 of the main text).

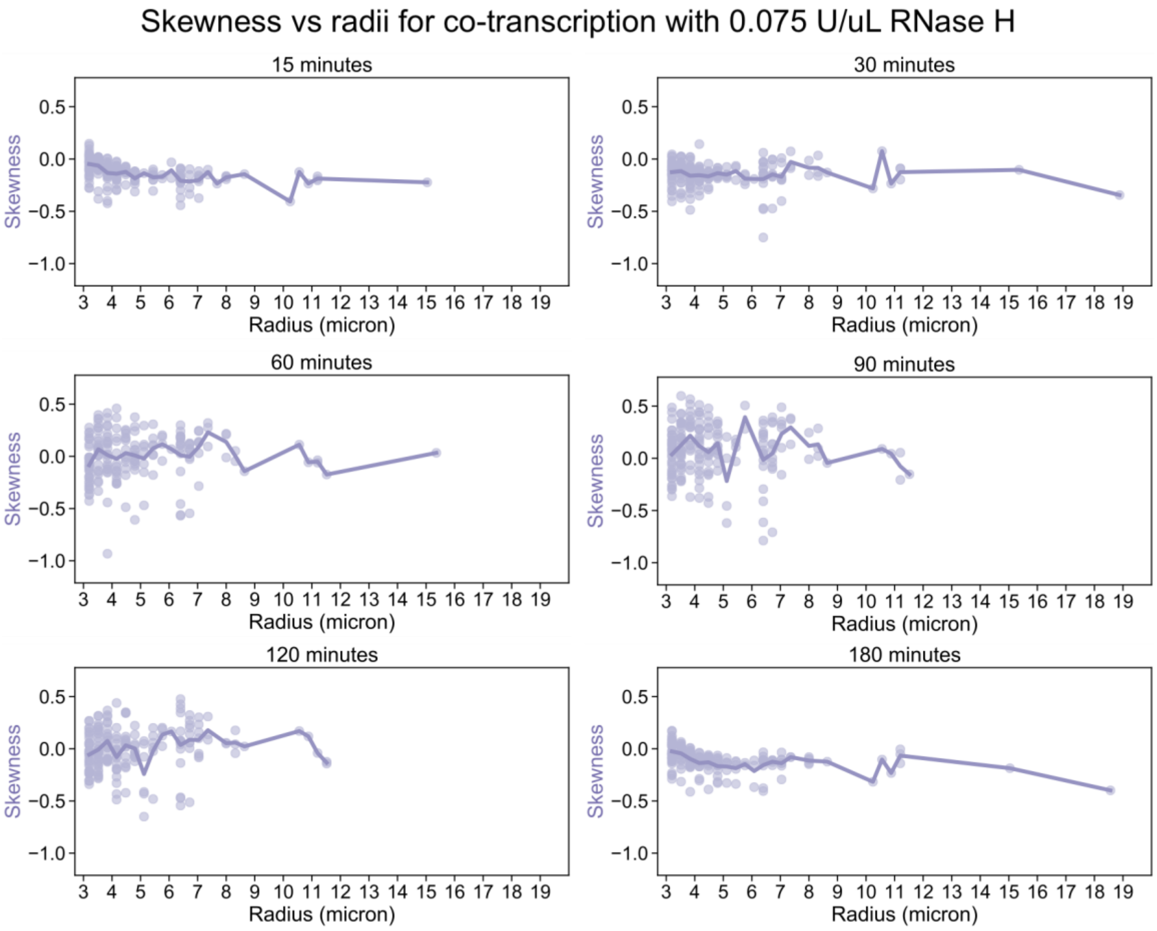

Figure S62: Skewness of droplets for the DNA-RNA hybrid nanotubes with co-transcription of RNA trigger from 100 nM template/gene and 0.075 U/µL RNase H in water-in-oil droplets experiment (Figure 6 of the main text).

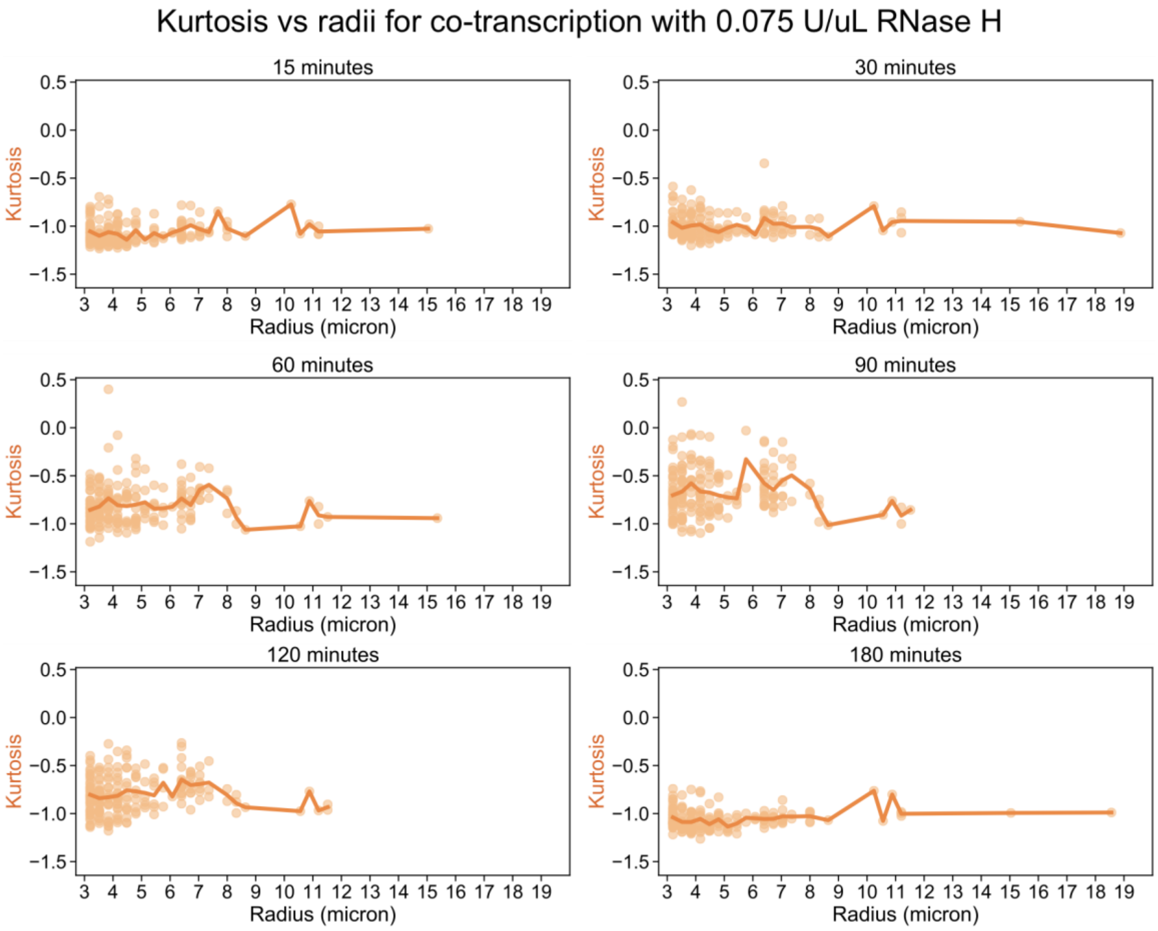

Figure S63: Kurtosis of droplets for the DNA-RNA hybrid nanotubes with co-transcription of RNA trigger from 100 nM template/gene and 0.075 U/µL RNase H in water-in-oil droplets experiment (Figure 6 of the main text).

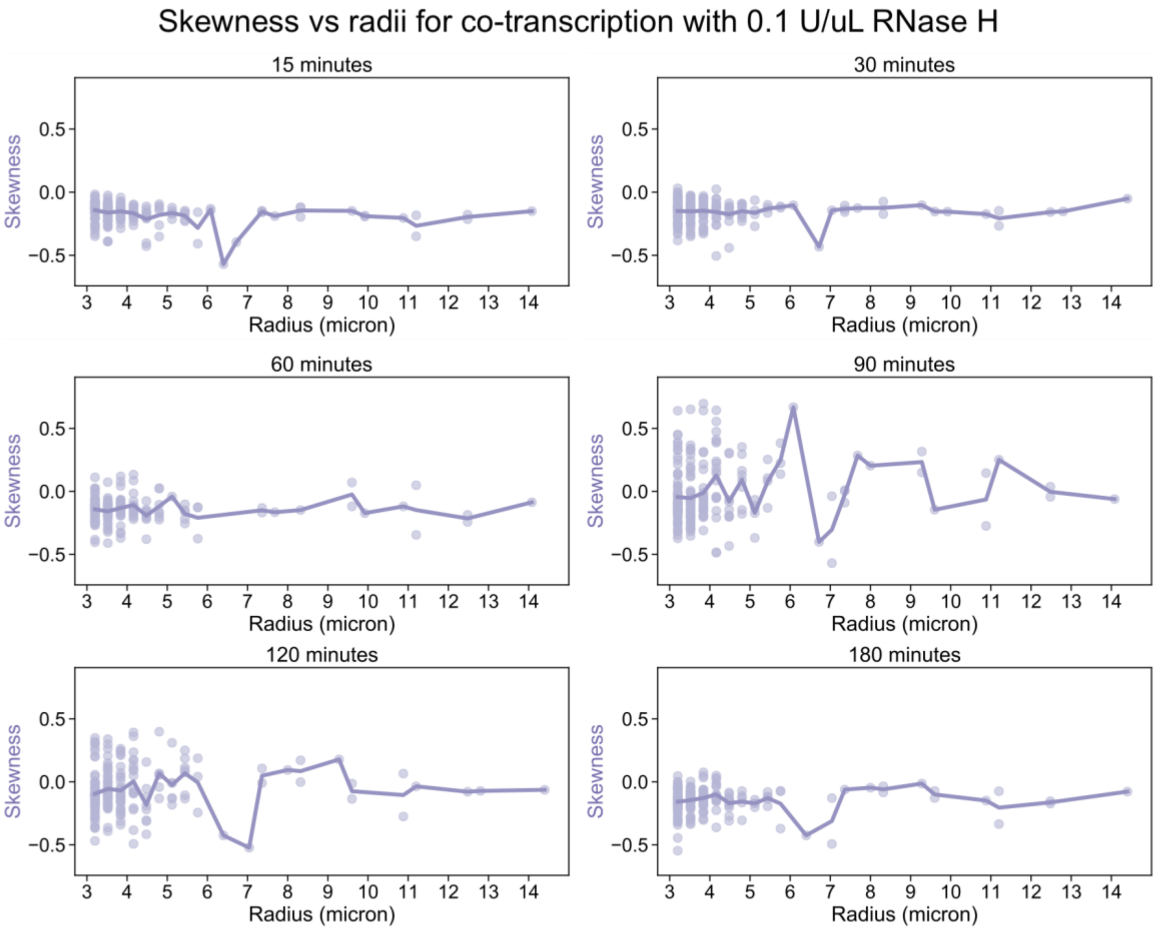

Figure S64: Skewness of droplets for the DNA-RNA hybrid nanotubes with co-transcription of RNA trigger from 100 nM template/gene and 0.1 U/µL RNase H in water-in-oil droplets experiment (Figure 6 of the main text).

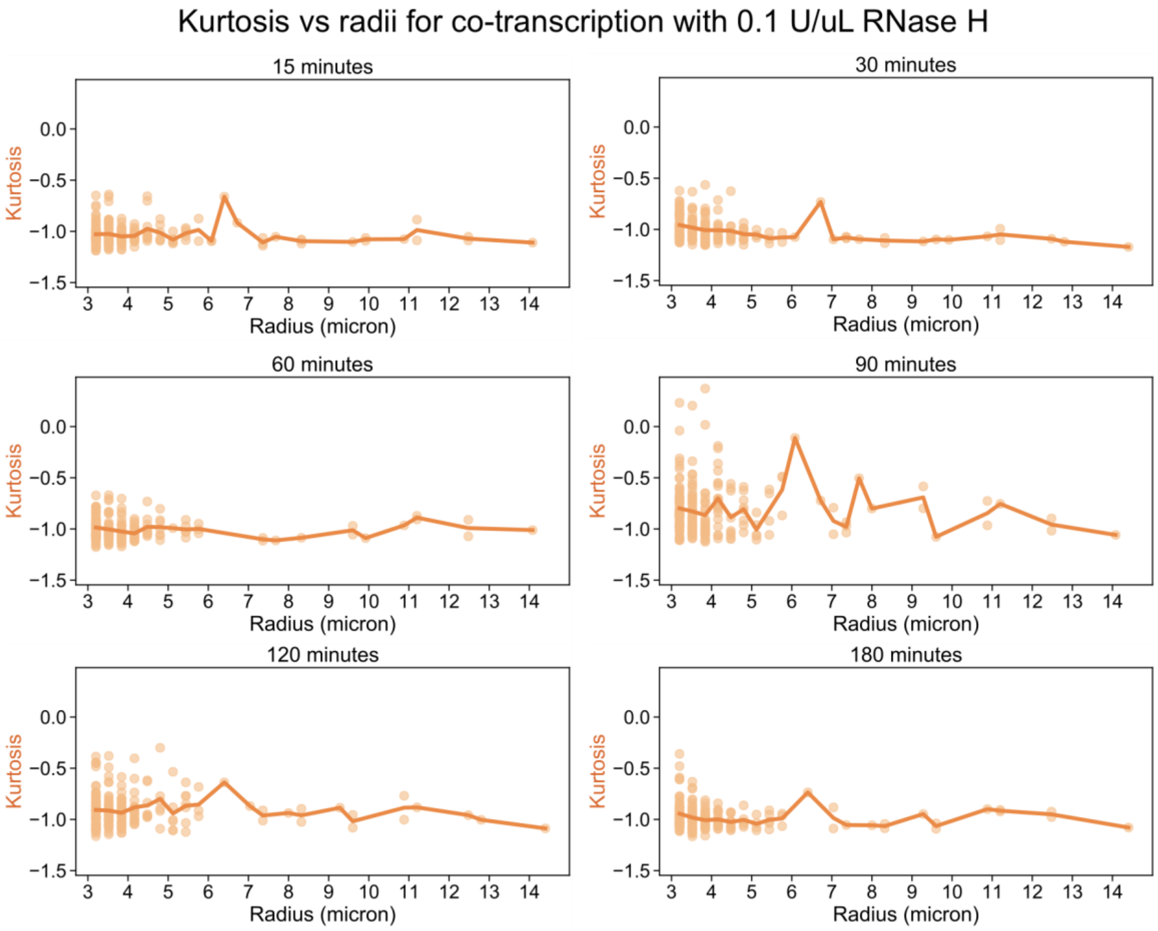

Figure S65: Kurtosis of droplets for the DNA-RNA hybrid nanotubes with co-transcription of RNA trigger from 100 nM template/gene and 0.1 U/µL RNase H in water-in-oil droplets experiment (Figure 6 of the main text).

S5.12 Two-tile nanotubes in bulk

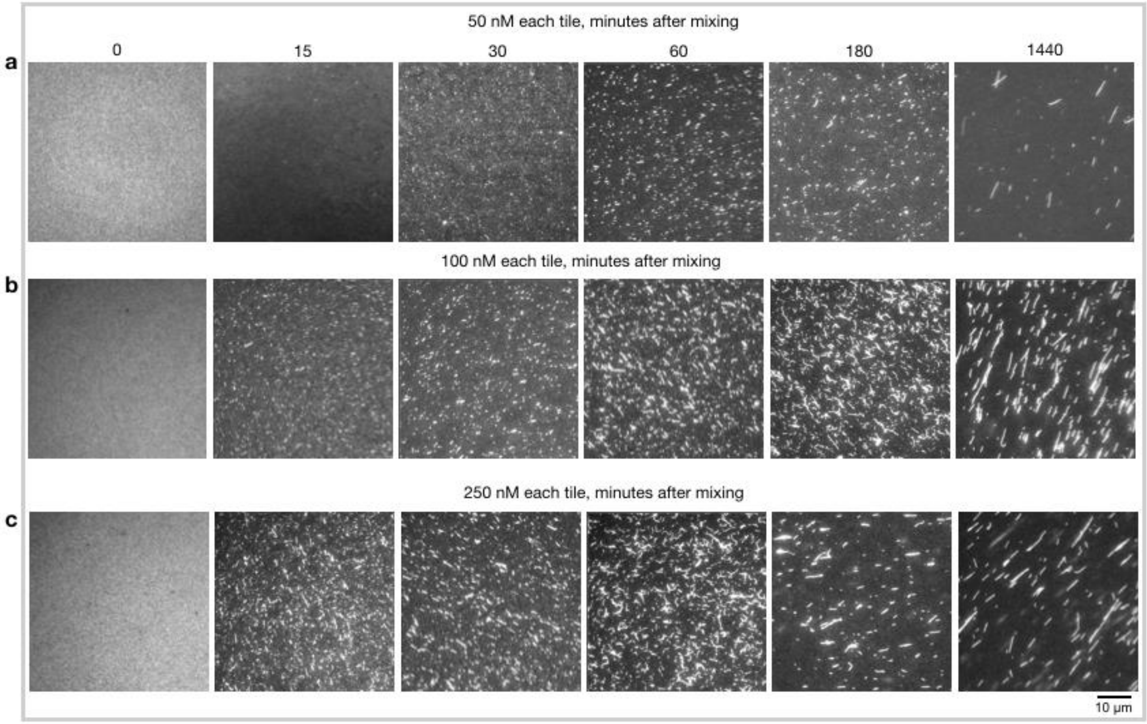

Figure S66: Isothermal assembly of non-encapsulated two-tile DNA nanotubes at room temperature. Tiles were pre-annealed and mixed immediately before the start of the imaging experiment. (a, b, c) Representative temporal sequence of fluorescence microscopy images of two-tile nanotubes mixtures at 50, 100, and 250 nM concentration for each tile respectively. Fluorescence microscopy images were collected after diluting each sample to 50 nM each tile at the time of slide preparation. Scale bar: 10 µm.

S5.13 Two-tile nanotubes in droplets for extended observations

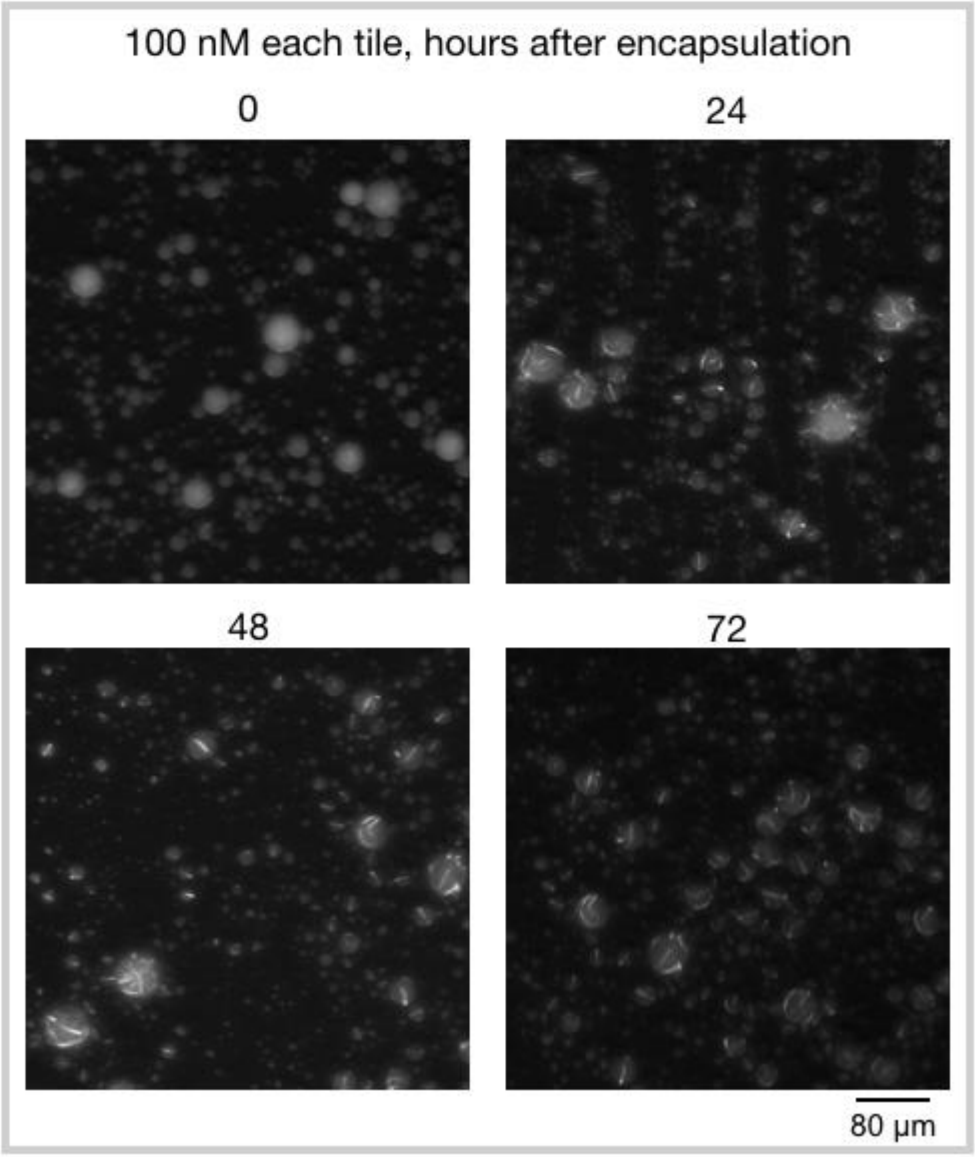

Figure S67: Encapsulated nanotube polymerization through 72 hours after encapsulation. Fluorescence images depict two-tile nanotube design with 100 nM each tile with 1x TAE buffer and 12.5 mM $MgCl_2$. Beyond 24h, we do not observe any discernible change in the nanotube morphology. Scale bar: 80 µm.

S5.14 Autofluorescence of water-in-oil droplets without fluorescently labeled strands

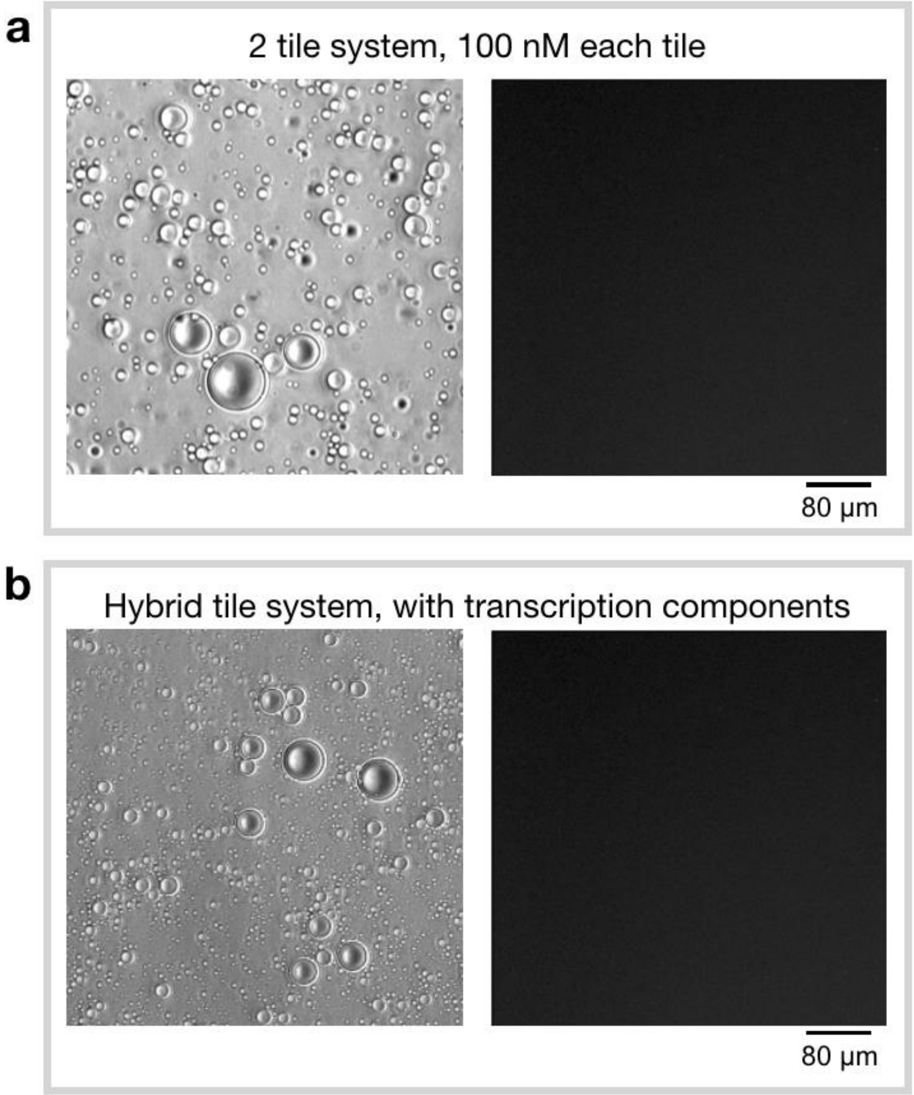

Figure S68: Background fluorescence of two nanotube designs without attached fluorescent dye encapsulated in water-in-oil droplets. We imaged nanotubes without an attached fluorescent dye in water-in-oil droplets to check for autofluorescence of either the droplets or surrounding oil medium. (a) Brightfield and Cy3 filter images of water-in-oil droplets containing the two-tile design, 100 nM each strand without an attached fluorescently labeled dye with 1x TAE buffer and 12.5 mM $MgCl_2$. (b) Brightfield and Cy3 filter images of water-in-oil droplets containing the hybrid design, 500 nM each strand without an attached fluorescent dye with 4 mM NTPs, 10 mM $MgCl_2$, 100 nM gene template, 2.5% w/v PEG, 2.5% v/v RNAP in 1X TXN buffer. These experiments confirm

that neither the droplets themselves nor the oil/surfactant medium are fluorescent. Scale bar: 80 µm.

S5.15 Increased background fluorescence for increasing tile concentration in two-tile design experiments

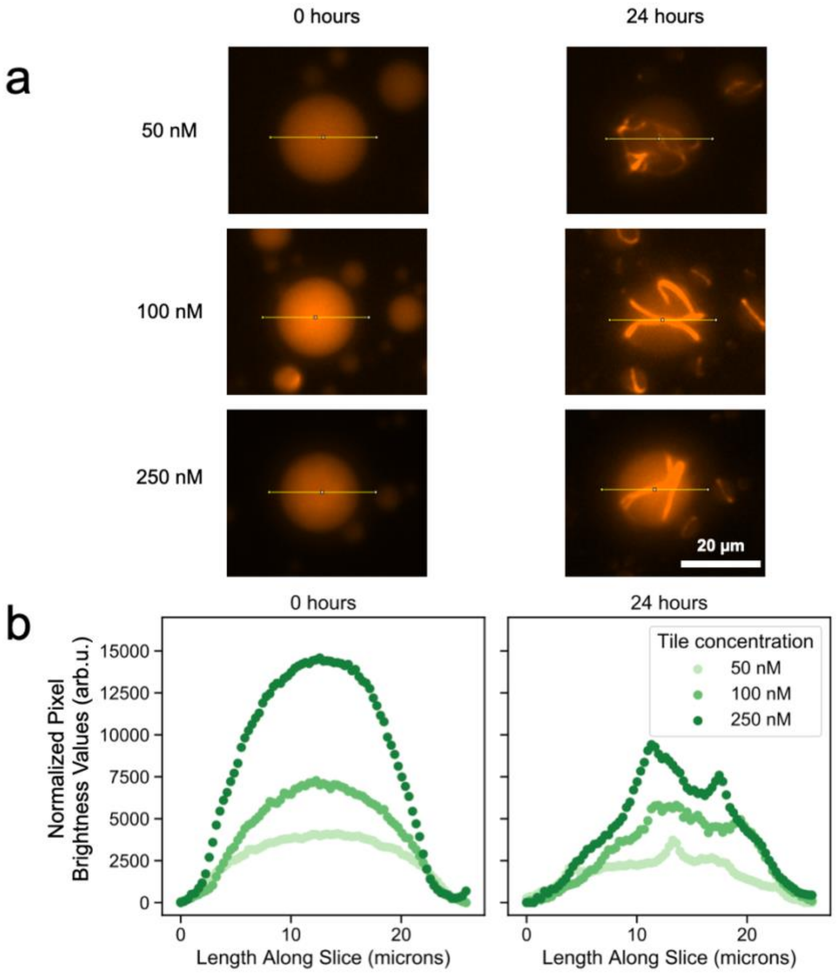

Figure S69: Comparison of the normalized pixel brightness values for different concentrations of two-tile nanotubes along a linear cross-section of a single droplet. a) Representative fluorescence microscopy images of a single droplet for each concentration of two-tile nanotubes reported in the main text at 0 and 24 hours. The yellow line indicates the linear segment for which normalized pixel brightness values are plotted in (b). The linear segment is the same length for all measurements. b) Normalized pixel brightness values along the length of the slice in (a) for 50, 100, 250 nM two-tile nanotubes at 0 and 24 hours. Pixel brightness values are normalized for each concentration and time point by subtracting the minimum brightness for that profile from each value such that the minimum brightness value for each profile is 0 arb.units. Scale bar: 20 µm.

S5.16 Length measurement of nanotubes using two-dimensional contour projection

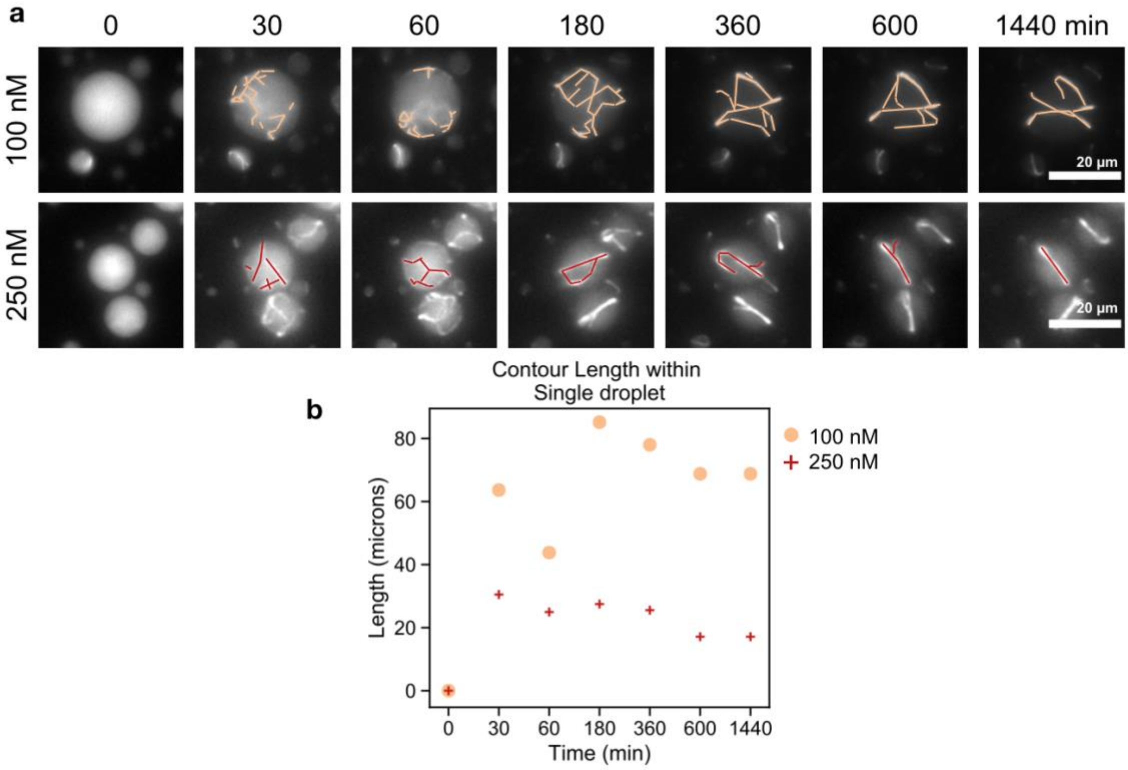

Figure S70: Measuring the projected contour length of nanotubes at each time point for 100 and 250 nM two-tile nanotubes within a single droplet. Epifluorescence microscopy images are a two-dimensional projection of signals coming from the focal plane, as well as planes above and below the focal plane. As a two-dimensional projection it is non-trivial, if not impossible, to determine the actual depth, angle, and length of observed nanotubes. Additionally, the number nanotubes which may be overlapping or intersecting are not distinguishable. For these droplets, we only measured each observable contour once, although the brightness and decreased curvature to the structures at 360 minutes onwards indicate there may be more than one nanotube along some contours. For these reasons, we believe contour measurement length to be misleading and non-comparable to previously reported nanotubes length measurements in bulk.[2,11] a) Representative microscopy images of the droplets for which contours are measured. Images with measured contours marked in orange (100 nM) and red (250nM) lines. Contours are measured by hand in Fiji using linear segments as indicated in (a). b) The sum of length of measured contours over time within each droplet. The length for the 100 nM sample is indicated by the light orange circles, while the 250 nM is indicated by the dark red crosses. In disagreement with both the model reported in this paper, and previous reports on nanotube assembly,[2,10] the total contour length decreases rather than reaches steady-state at some maximum total length, further confirming that contour length is not an accurate method for quantifying nanotube assembly inside droplets. Scale bar: 20 µm.

S5.17 Effect of DNase I on two-tile DNA nanotubes

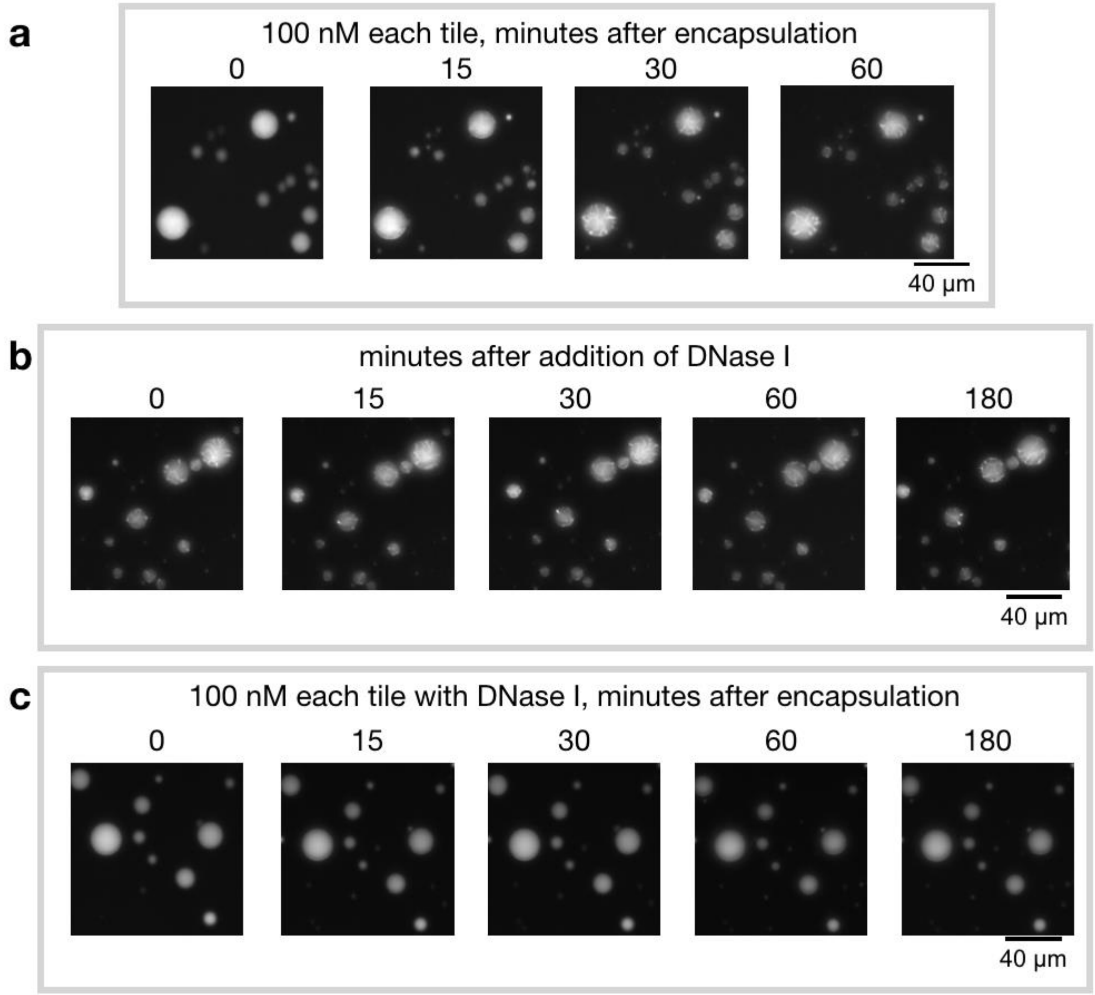

Figure S71: DNase I can inhibit growth of nanotubes by degrading the monomer tile made of DNA oligomers but DNase I cannot penetrate through water-in-oil droplets if added to the oil phase (a) Encapsulated DNA nanotubes were grown (two-tile nanotube design with 100 nM each tile with 1x TAE buffer and 12.5 mM $MgCl_2$) for 60 minutes. (b) 5μl of 1U/μl of DNase 1 to the oil phase to see if DNase can penetrate through the droplet but no change was observed in the morphology of nanotubes inside the droplets. (c) Control experiment where DNase was encapsulated with the tile mixture inside the droplets (two-tile nanotube design with 100 nM each tile with 1x TAE buffer and 12.5 mM $MgCl_2$ and 5μl of 1U/μl of DNase 1). DNase 1 successfully prevents any nanotube formation. Scale bar: 40 μm.

Supplementary Note 6 Computational modeling

We built a series of deterministic models to illustrate the temporal dynamics of the following processes:
1) tile assembly
2) tile activation using a single stranded activator, also called trigger, and
3) tile activation/deactivation using transcription of RNA trigger and degradation (mediated by RNase H) of DNA-RNA hybrid complexes.

The models described here are derived based on models of tile assembly developed in (Zhang et al. 2013)[11], and from models of transcriptional circuits developed in (Kim et al., 2006, Weitz et al. 2014)[12,13]. Both classes of models are deterministic ordinary differential equations (ODEs), that rely on the assumption that molecular species are at high concentration and stochastic effects can be neglected. Our encapsulated experiments satisfy this assumption, as all DNA species are present at concentration above 50 nM. For experiments including RNase H, partitioning noise may affect the system operation as RNase H is likely present only at few nanomolar concentration, but we focus on droplets with a diameter above 6 µm, which means more than 100 copies of the enzyme are present in each droplet. Deterministic ODEs are still suited to capture this scenario.

The models we developed were not fitted to our data. The purpose of our modeling efforts is to illustrate our understanding of experimental behaviors observed in our experiments, not to recapitulate such behaviors quantitatively. To solve the ODEs via numerical integration, we adopted parameters provided in the literature. These parameters were identified in the cited literature by fitting the models to data collected from experiments comparable to the ones described here, but not encapsulated in droplets.

S6.1 Tile assembly models

Our goal is to model the fraction of tiles that are assembled into growing nanotubes. A simple ODE model that captures tile assembly into nanotubes was proposed and fitted to data by (Zhang et al. 2013)[11].
The model by Zhang et al. includes two reactions:

Nucleation

$$n \cdot Tile \xrightarrow{k_{nuc}} Nanotube$$

Elongation

$$Nanotube + Tile \xrightarrow{k_{elong}} Nanotube$$

Using the law of mass action, these reactions were mapped to two ODEs describing the temporal evolution of free tiles and nanotubes:

$$\frac{d\,[Tile]}{dt} = -nk_{nuc}[Tile]^n - k_{elong}[Tile][Nanotube] \qquad (1)$$
$$\frac{d\,[Nanotube]}{dt} = nk_{nuc}[Tile]^n$$

Assuming the total tile concentration T^{tot} remains constant, the fraction of assembled tiles can be derived by subtracting the concentration of free tiles from T^{tot}. The model parameters, nucleation stoichiometry (n), nucleation rate parameter (k_{nuc}) and elongation rate parameter (k_{elong}), were fitted and validated experimentally in Zhang et al. This model does not include depolymerization, which in practice reduces growth rate; also this model does not include nanotube joining (Ekani Nkodo et al. 2004, Mardanlou et al. 2018)[13,14]. Thus, it likely overestimates assembly rates and nanotube number.

We adapted the Zhang model to a scenario in which two separate tile species interact to nucleate and then polymerize, however the kinetic parameters of the reactions are identical (for simplicity) for each species. Nucleation and elongation can only occur if both tile species are present. Adopting for consistency the same notation as in the Zhang models, we have:

Nucleation: we assume that only half the number of tiles of each species are needed to achieve the nucleation stoichiometry n

$$\frac{n}{2} \cdot Tile\ A + \frac{n}{2} \cdot Tile\ B \xrightarrow{k_{nuc}} Nanotube$$

Elongation: Because we use a two-tile design in which tiles form a perpendicular ring pattern, we model nanotubes elongation using the same reaction assumed by Zhang for the single-tile design. However, we also assume on average half the nanotube population is growing a Tile A edge and the other half is growing a Tile B edge; this assumption is reflected in the ODE model (2) that follows.

$$Nanotube + Tile\ A \xrightarrow{k_{elong}} Nanotube$$
$$Nanotube + Tile\ B \xrightarrow{k_{elong}} Nanotube$$

Using the law of mass action, we derive the following ODEs:

$$\frac{d\,[Tile\ A]}{dt} = \frac{-n}{2} k_{nuc} [Tile\ A]^{\frac{n}{2}} [Tile\ B]^{\frac{n}{2}} - \frac{1}{2} k_{elong} [Tile\ A][Nanotube] \quad (2)$$

$$\frac{d\,[Tile\ B]}{dt} = \frac{-n}{2} k_{nuc} [Tile\ A]^{\frac{n}{2}} [Tile\ B]^{\frac{n}{2}} - \frac{1}{2} k_{elong} [Tile\ B][Nanotube]$$

$$\frac{d\,[Nanotube]}{dt} = k_{nuc} [Tile\ A]^{\frac{n}{2}} [Tile\ B]^{\frac{n}{2}}$$

Adopting the parameters in Table S6, these ODEs were integrated numerically using MATLAB with a custom script implementing the Euler method with a step size of 0.1 s.

We assumed the total concentration of each tile to be 50 nM, 100 nM or 250 nM (for consistency with the experiments in Figure 3 of the main text). The computed fraction of assembled tiles and the estimated number of nanotubes in a droplet of 3µm radius are shown in Fig. S66. This plots illustrates that the higher the tile concentration, the faster tiles are incorporated into nanotubes, and the faster nanotubes nucleate, reaching higher numbers.

In our experiments monitoring assembly of nanotubes in droplets (Figure 3 of the main text) nanotubes appear to nucleate at higher tile concentration in agreement with the predictions of this simple model. However the droplet brightness suggests that a large number of tiles may remain unpolymerized beyond 2-3 hours, suggesting that depolymerization may be a non-negligible reaction. Unlike the predicted fraction of assembled tiles, skewness appears to increase faster at lower concentrations, likely due to the high fluorescence background at high tile concentrations.

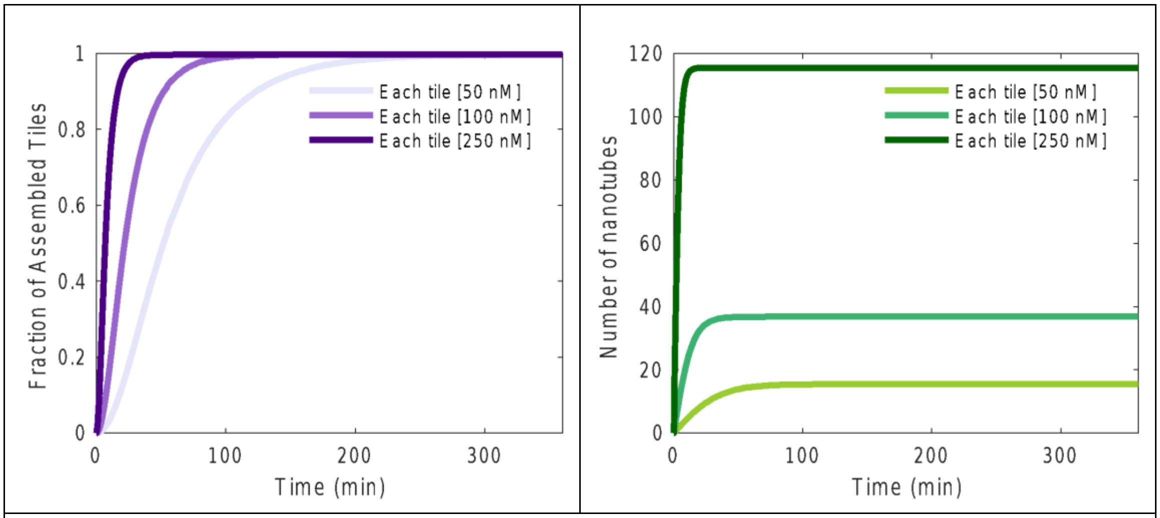

Figure S72: Predicted fraction of assembled tiles and number of nanotubes in a droplet of 3µm radius using model (2). Simulation parameters are listed in Table S6.

S6.2 Model capturing transcription of RNA and RNA-mediated tile activation and assembly

We now derive a simple model for activation of tiles through an RNA molecule that is transcribed by a synthetic gene.

First, activation of tiles can be modeled with the macroscopic chemical reaction:

$$RNA_{Trigger} + Inactive\ Tile \xrightarrow{k_{TAI}} Active\ Tile$$

Next, we assume the system includes a constant amount of synthetic gene, or template, that produces RNA trigger, which we denote $T_{RNA.}$ RNA polymerase binds to the template and produces RNA trigger according to the following steps:

$$RNAP + T_{RNA} \underset{k-}{\overset{k+}{\Leftrightarrow}} RNAP \cdot T_{RNA} \xrightarrow{K_{CAT,ON}} RNA_{Trigger} + RNAP + T_{RNA}$$

We further assume that RNAP loses activity according to a first order reaction:

$$RNAP \xrightarrow{k_{loss}} 0$$

Because in our experiments we used the Ampliscribe Flash transcription kit that recommends 4 hour incubation (see section S4.6), we assumed k_{loss} to be 0.00019/s which corresponds to a loss of more than 50% activity in four hours of reaction.

Active tiles nucleate and contribute to nanotube elongation according to model (1). The additional ODEs for RNA production and tile activation are derived using the law of mass action:

(3)

$$\frac{d\,[T_{RNA}]}{dt} = K_{CAT,ON}\ [RNAP \cdot T_{RNA}] + k_{-}\ [RNAP \cdot T_{RNA}] - k_{+}[RNAP \cdot T_{RNA}]$$

$$\frac{d[RNA_{trigger}]}{dt} = K_{CAT,ON}\ [RNAP \cdot T_{RNA}] - k_{TAI}[RNA_{Trigger}][Inactive\ Tile]$$

$$\frac{d\,[Inactive\ Tile]}{dt} = -k_{TAI}[RNA_{Trigger}][Inactive\ Tile]$$

$$\frac{d\,[Active\ Tile]}{dt} = k_{TAI}[RNA_{Trigger}][Inactive\ Tile]$$

$$\frac{d\,[RNAP \cdot T_{RNA}]}{dt} = -K_{CAT,ON}\ [RNAP \cdot T_{RNA}] - k_{-}[RNAP \cdot T_{RNA}] + k_{+}[RNAP][T_{RNA}]$$

$$\frac{d\,[RNAP]}{dt} = K_{CAT,ON}\ [RNAP \cdot T_{RNA}] + k_{-}[RNAP \cdot T_{RNA}] - k_{+}[RNAP][T_{RNA}] - k_{loss}[RNAP]$$

These equations were numerically integrated with custom MATLAB scripts, using the parameters in Table S6.

The manufacturer did not provide information about the estimated concentration RNAP batch we used in our experiments. To estimate the RNAP amount based on the volume we used in the encapsulated reactions (2.5% v/v of the transcription and tile mix), we reasoned that the transcription kits we used were optimized for rapid yield, like kits adopted in previous research on transcriptional circuits (Weitz et al. 2014)[13]. Thus, we assumed that the stock concentration of our enzyme should be in the range of 3-4μM (Weitz et al. 2014)[13]. Remaining consistent with the 218 nM concentration of RNAP estimated in (Weitz et al. 2014)[13] for experiments that used a 10% (v/v) RNAP, we assumed the RNAP concentration to be 54 nM.

Figure S67 shows the fraction of assembled tiles computed using model (3). This simulation illustrates that tile assembly proceeds more rapidly when a higher template concentration is used. At low template concentrations, assembly proceeds more slowly and settles to a low equilibrium level due to the assumption that RNAP loses activity and becomes inactive after a period of time.

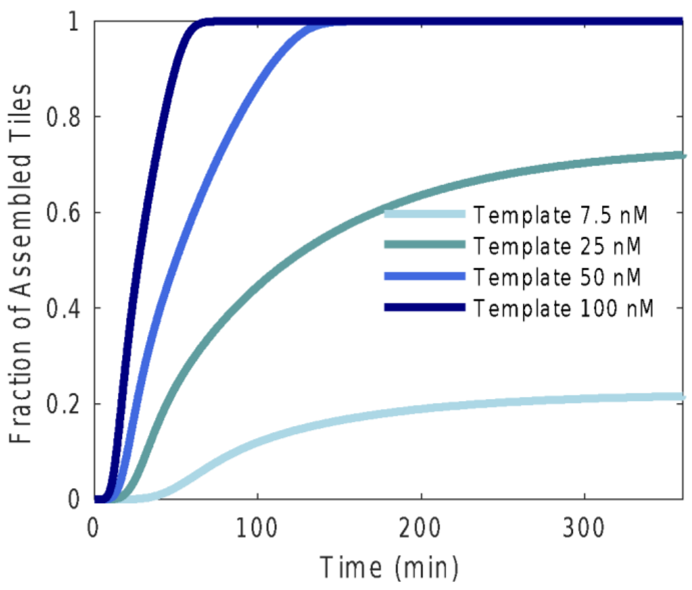

Figure S73: Illustrative simulation, obtained using model (3), showing the temporal evolution of the fraction of assembled tiles as the synthetic gene template varies between 7.5 and 100 nM.

This plot is also reported in Figure 5 of the main text. Simulation parameters are listed in Table S6.

S6.3 Model capturing transcription and degradation of RNA and RNA-mediated tile activation and assembly

We model the effects of RNase H by assuming that active tiles, assembled tiles, and nucleated nanotubes are degraded and revert to inactive tiles, with a net consumption of RNA.

These processes are modeled through the following reactions:

$$RNase\ H + Active\ Tile \underset{k-,H}{\overset{k+,H}{\Longleftrightarrow}} RNase\ H \cdot Active\ Tile \xrightarrow{K_{CAT,H}} Inactive\ Tile + RNase\ H$$

$$RNase\ H + Nanotube \underset{k-,H}{\overset{k+,H}{\Longleftrightarrow}} RNase\ H \cdot Nanotube \xrightarrow{K_{CAT,H}} Inactive\ Tile + RNase\ H$$

$$RNase\ H + Assembled\ Tile \underset{k-,H}{\overset{k+,H}{\Longleftrightarrow}} RNase\ H \cdot Nanotube \xrightarrow{K_{CAT,H}} Inactive\ Tile + RNase\ H$$

Using conservation of mass for the tiles, the concentration of the Assembled Tile species can be found as:

$$\begin{aligned} Assembled\ Tile \\ &= [Inactive\ Tile]_0 - [Inactive\ Tile] - [Active\ Tile] \\ &- [RNase\ H \cdot Active\ Tile] - [Nucleus] - [RNase\ H \cdot Nucleus] \\ &- [RNase\ H \cdot Assembled\ Tile] \end{aligned}$$

Using the law of mass action, we can then find the following 11 ODEs:

(4)

$$\frac{d\ [T_{RNA}]}{dt} = K_{CAT},_{ON}\ [RNAP \cdot T_{RNA}] + k_-\ [RNAP \cdot T_{RNA}] - k_+[RNAP \cdot T_{RNA}]$$

$$\frac{d[RNA_{trigger}]}{dt} = K_{CAT},_{ON}\ [RNAP \cdot T_{RNA}] - k_{AI}[RNA_{Trigger}][Inactive\ Tile]$$

$$\begin{aligned} \frac{d\ [Inactive\ Tile]}{dt} \\ &= -k_{AI}\left[RNA_{Trigger}\right][Inactive\ Tile] + K_{CAT},_{H}\ [RNase\ H \cdot Active\ Tile] \\ &+ K_{CAT},_{H}\ [RNase\ H \cdot Nanotube] \\ &+ K_{CAT},_{H}\ [RNase\ H \cdot Assembled\ Tile] \end{aligned}$$

$$\frac{d\,[Active\ Tile]}{dt} = k_{AI}[RNA_{Trigger}][Inactive\ Tile] + k_{-,H}\,[RNase\ H \cdot Active\ Tile] - nk_{nuc}[Active\ Tile]^n - k_{+,H}\,[RNase\ H][Active\ Tile] - k_{elong}[Nanotube][Active\ Tile]$$

$$\frac{d\,[Nanotube]}{dt} = nk_{nuc}[Active\ Tile]^n - k_{+,H}\,[RNase\ H][Active\ Tile] + k_{-,H}\,[RNase\ H \cdot Nanotube]$$

$$\frac{d\,[RNase\ H \cdot Active\ Tile]}{dt} = k_{+,H}[RNase\ H][Active\ Tile] - k_{-H}[RNase\ H \cdot Active\ Tile] - K_{CAT,H}\,[RNase\ H \cdot Active\ Tile]$$

$$\frac{d\,[RNase\ H \cdot Nanotube]}{dt} = k_{+,H}[RNase\ H][Nanotube] - k_{-H}[RNase\ H \cdot Nanotube] - K_{CAT,H}\,[RNase\ H \cdot Nanotube]$$

$$\frac{d\,[RNase\ H \cdot Assembled\ Tile]}{dt} = k_{+,H}[RNase\ H][Assembled\ Tile] - k_{-H}[Assembled\ Tile] - K_{CAT,H}\,[Assembled\ Tile]$$

$$\frac{d[RNase\ H]}{dt} = K_{CAT,H}\,[RNase\ H \cdot Assembled\ Tile] + K_{CAT,H}[RNase\ H \cdot Nanotube] + K_{CAT,H}[RNase\ H \cdot Active\ Tile] + k_{-,H}[RNase\ H \cdot Assembled\ Tile] + k_{-,H}[RNase\ H \cdot Nanotube] + k_{-,H}[RNase\ H \cdot Active\ Tile] - k_{+,H}[RNase\ H][Assembled\ Tile] - k_{+,H}[RNase\ H][Nanotube] - k_{+,H}[RNase\ H][Active\ Tile]$$

$$\frac{d\,[RNAP \cdot T_{RNA}]}{dt} = -K_{CAT,ON}\,[RNAP \cdot T_{RNA}] - k_{-}[RNAP \cdot T_{RNA}] + k_{+}[RNAP][T_{RNA}]$$

$$\frac{d\,[RNAP]}{dt} = K_{CAT,ON}\,[RNAP \cdot T_{RNA}] + k_{-}[RNAP \cdot T_{RNA}] - k_{+}[RNAP][T_{RNA}] - k_{loss}[RNAP]$$

Model (4) was integrated numerically using custom MATLAB scripts, and using the parameters in Table S6. For consistency we assumed 54 nM RNAP concentration, the same value used to generate Fig. S67. Because we were not able to obtain from the manufacturer the estimated stock concentration of RNase H, we estimated a plausible RNase concentration using the estimates for a similar product reported in (Weitz et al, 2014)[12]. Our reasoning relies on the assumption

that for practical purposes the RNase H activity/units is comparable between Promega RNase H (this project) and Ambion RNase H (Weitz et al.), although their definition is not the same. For the Weitz paper, based on their estimate of stock concentration, RNase H volume added to the aqueous phase, and vendors' units per microliter of the stock, we estimated that 0.015 units/µl correspond to 1 nM RNase H concentration. Thus, for these simulations, we estimate that a 0.025 units/µl RNase H amount corresponds to a concentration of 1.6 nM, 0.05 units/µl correspond to 3.2 nM and so on.

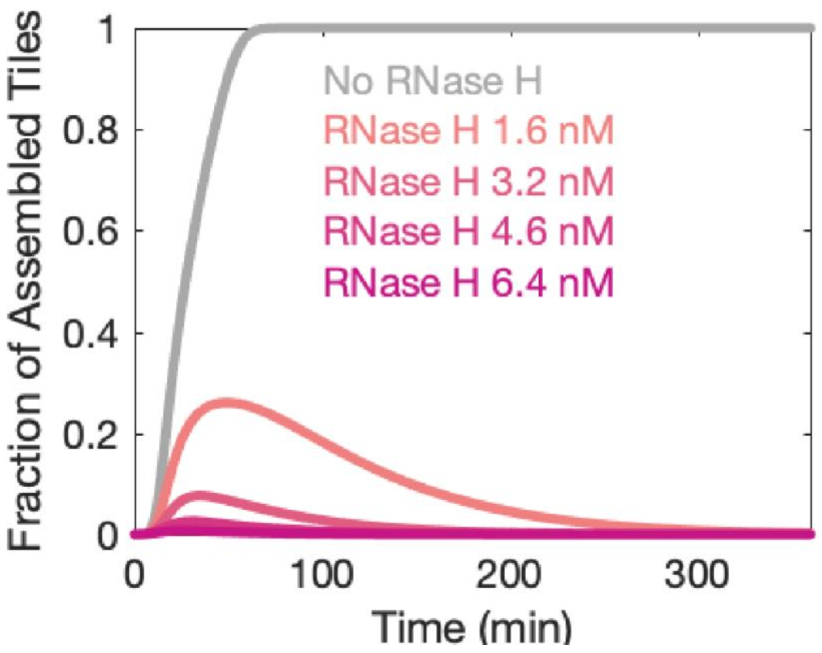

Fig. S74: Illustrative simulation showing the transient pulse predicted by the simulated fraction of assembled tiles, as the concentration of RNase H is varied. This plot is also reported in Fig. 6 of the main text.

Parameter	Value and units	Reference	Range (literature)
k_A	6.96 x 10^4/M/s	Kim et al., 2006[12], Zhang et al, 2013[11]	10^4-10^7
$k_{-, H}$	0.125 /M/s	Weitz et al., 2014[13]	0.01
$k_{+, H}$	7.13 x 10^5/M/s	Weitz et al., 2014[13]	10^5 - 10^7
$K_{CAT, H}$	0.552 /s	Weitz et al., 2014[13]	0.01 - 1
n	2.5	Zhang et al. 2014[16]	N/A
k_{nuc}	2.0 x 10^5 /M/s	Zhang et al. 2013[11]	N/A
k_{elong}	3.4 x 10^6 /M/s	Zhang et al. 2013[11]	N/A
k_+	1.9 x 10^5 /M/s	Weitz et al., 2014[13]	N/A
k_-	0.0446 /M/s	Weitz et al., 2014[13]	N/A
$K_{CAT, ON}$	0.0186 /M/s	Weitz et al., 2014[13]	N/A
k_{loss}	0.00019/s		

Table S6: *Parameters used in computational simulations.*[6]

References

1 Rothemund, P. W. *et al.* Design and characterization of programmable DNA nanotubes. *Journal of the American Chemical Society* **126**, 16344-16352 (2004).

2 Agarwal, S. & Franco, E. Enzyme-driven assembly and disassembly of hybrid DNA–RNA nanotubes. *Journal of the American Chemical Society* **141**, 7831-7841 (2019).

3 Rothemund, P. W. K. *et al.* Design and Characterization of Programmable DNA Nanotubes. *Journal of the American Chemical Society* **126**, 16344-16352, doi:10.1021/ja044319l (2004).

4 Milligan, J. F., Groebe, D. R., Witherell, G. W. & Uhlenbeck, O. C. Oligoribonucleotide synthesis using T7 RNA polymerase and synthetic DNA templates. *Nucleic acids research* **15**, 8783-8798 (1987).

5 Van der Walt, S. *et al.* scikit-image: image processing in Python. *PeerJ* **2**, e453 (2014).

6 M.A. Klocke, P. Pungchai. Dynamic self-assembly of compartmentalized DNA nanotubes, dropletDetection_dynamicSelfAssemblyProject. *Zenodo*, doi:10.5281/zenodo.4763253 (2021).

7 von Hippel, P. T. Mean, Median, and Skew: Correcting a Textbook Rule. *Journal of Statistics Education* **13**, null-null, doi:10.1080/10691898.2005.11910556 (2005).

8 DeCarlo, L. T. On the meaning and use of kurtosis. *Psychological Methods* **2**, 292-307, doi:https://doi.org/10.1037/1082-989X.2.3.292 (1997).

9 Fisher, R. A. Statistical methods for research workers. *Springer*, 66-70, doi:https://doi.org/10.1007/978-1-4612-4380-9_6 (1992).

10 Green, L. N. *et al.* Autonomous dynamic control of DNA nanostructure self-assembly. *Nature chemistry* **11**, 510-520 (2019).

11 Zhang, D. Y., Hariadi, R. F., Choi, H. M. T. & Winfree, E. Integrating DNA strand-displacement circuitry with DNA tile self-assembly. *Nature Communications* **4**, 1965, doi:10.1038/ncomms2965 (2013).

12 Kim, J., White, K. S. & Winfree, E. Construction of an in vitro bistable circuit from synthetic transcriptional switches. *Molecular systems biology* **2**, 68 (2006).

13 Weitz, M. *et al.* Diversity in the dynamical behaviour of a compartmentalized programmable biochemical oscillator. *Nature chemistry* **6**, 295-302 (2014).

14 Ekani-Nkodo, A., Kumar, A. & Fygenson, D. K. Joining and scission in the self-assembly of nanotubes from DNA tiles. *Physical review letters* **93**, 268301 (2004).

15 Mardanlou, V. *et al.* A coarse-grained model captures the temporal evolution of DNA nanotube length distributions. *Natural Computing* **17**, 183-199 (2018).

16 Zhang, F., Nangreave, J., Liu, Y. & Yan, H. Structural DNA Nanotechnology: State of the Art and Future Perspective. *Journal of the American Chemical Society* **136**, 11198-11211, doi:10.1021/ja505101a (2014).
