# Supplementary figures and images for: Dynamic self-assembly of compartmentalized DNA nanotubes
Source: Nat Commun. 2021 Jun 11;12:3557. doi: 10.1038/s41467-021-23850-1 (PMC8196065; doi:10.1038/s41467-021-23850-1)

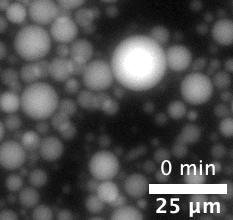

Supplement: Supplementary file 2 — Supplementary Movie 1 [file 41467_2021_23850_MOESM2_ESM.gif]

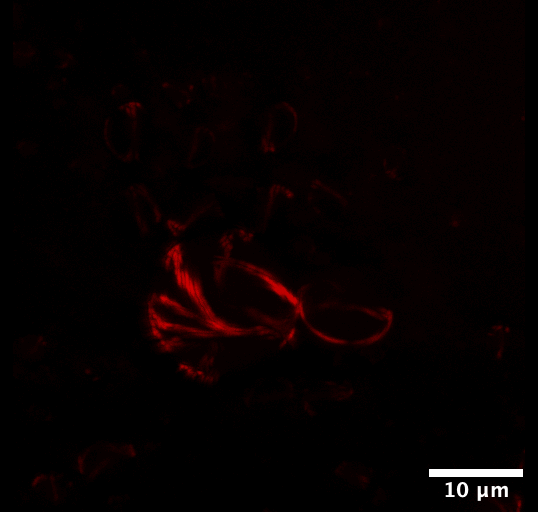

Supplement: Supplementary file 3 — Supplementary Movie 2 [file 41467_2021_23850_MOESM3_ESM.gif]

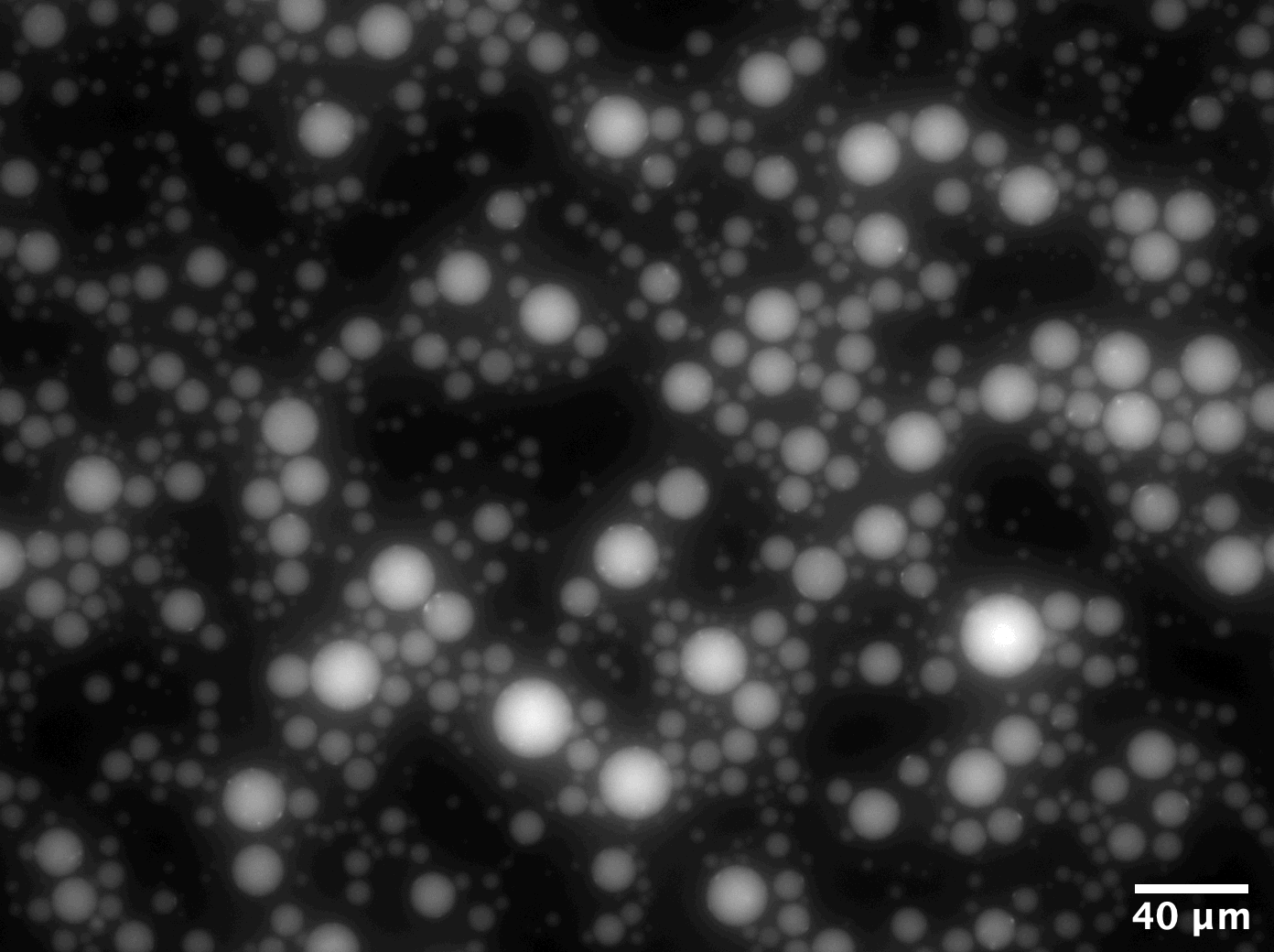

Supplement: Supplementary file 4 — Supplementary Movie 3 [file 41467_2021_23850_MOESM4_ESM.gif]

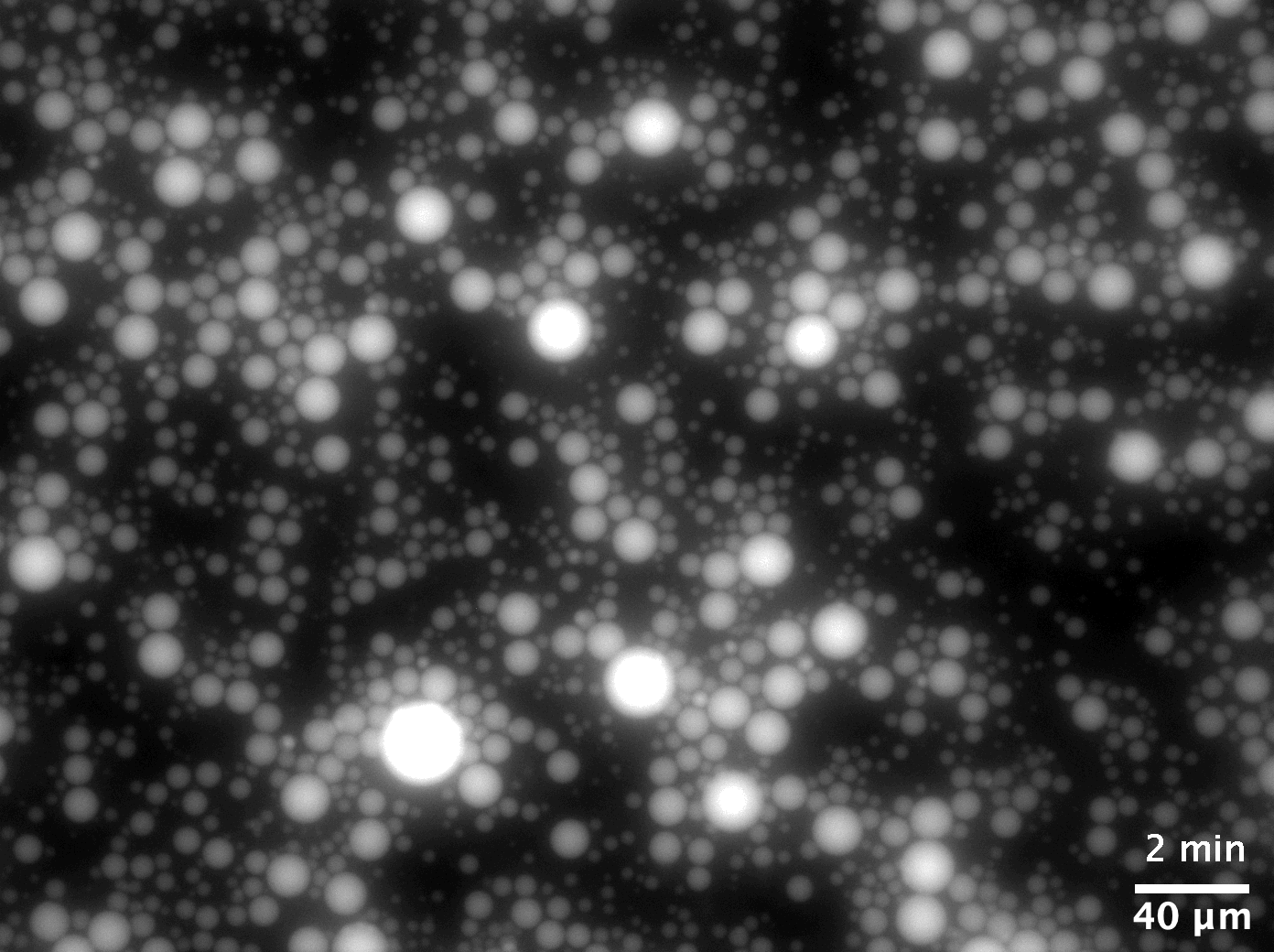

Supplement: Supplementary file 5 — Supplementary Movie 4 [file 41467_2021_23850_MOESM5_ESM.gif]

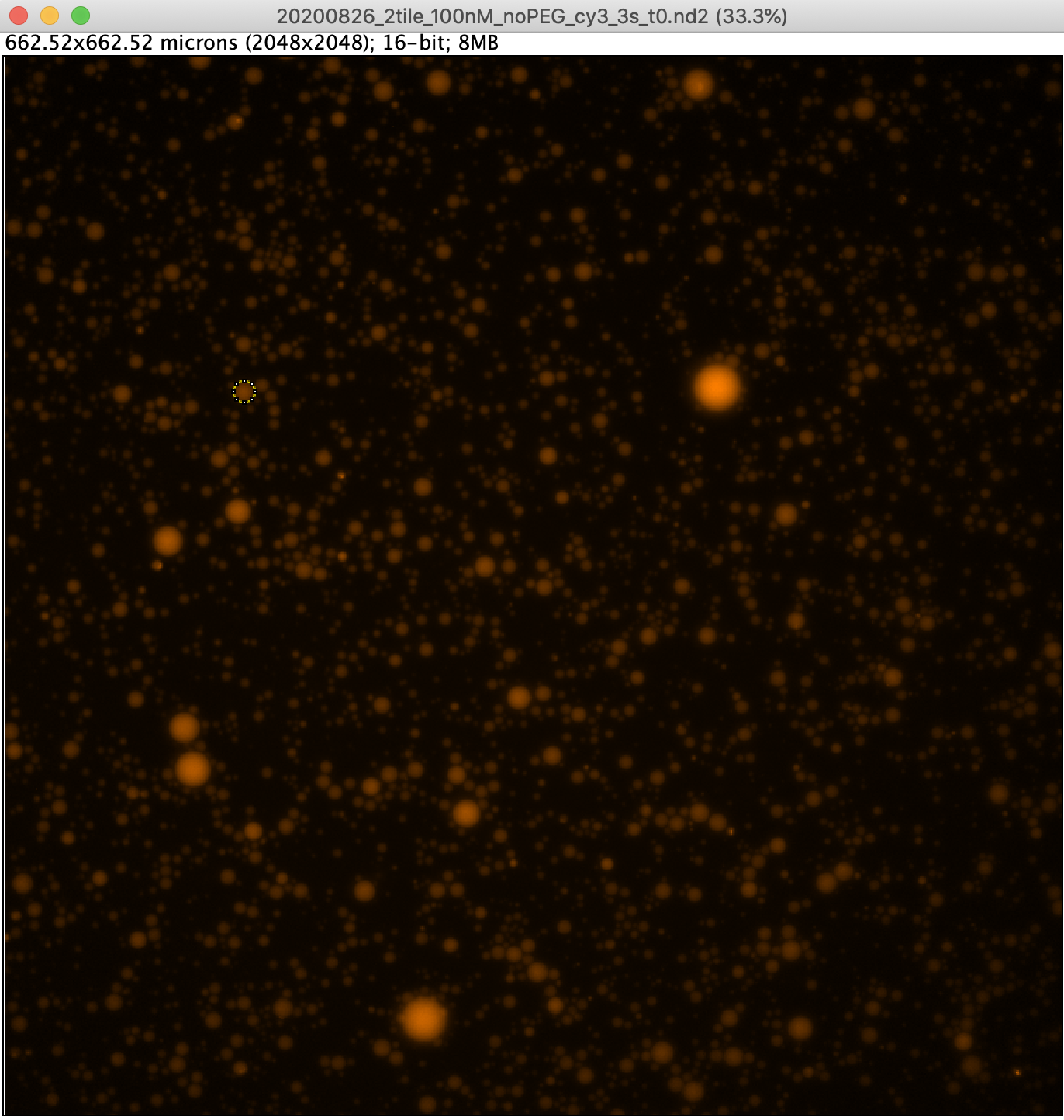

Supplement: Supplementary file 8 — Source Data [file 41467_2021_23850_MOESM8_ESM.zip › sourceData/figure2/fig2_d1_labeledOnImg.png]
