# Supplementary material for: Dynamic self-assembly of compartmentalized DNA nanotubes
Source: Nat Commun. 2021 Jun 11;12:3557. doi: 10.1038/s41467-021-23850-1 (PMC8196065; doi:10.1038/s41467-021-23850-1)
Supplement: Supplementary file 6 — Reporting Summary [file 41467_2021_23850_MOESM6_ESM.pdf]

nature research

Corresponding author(s): Elisa Franco

Last updated by author(s): 5/13/2021

Reporting Summary

Nature Research wishes to improve the reproducibility of the work that we publish. This form provides structure for consistency and transparency in reporting. For further information on Nature Research policies, see our Editorial Policies and the Editorial Policy Checklist.

Statistics

For all statistical analyses, confirm that the following items are present in the figure legend, table legend, main text, or Methods section.

n/a	Confirmed	
☐	☒	The exact sample size (*n*) for each experimental group/condition, given as a discrete number and unit of measurement
☒	☐	A statement on whether measurements were taken from distinct samples or whether the same sample was measured repeatedly
☒	☐	The statistical test(s) used AND whether they are one- or two-sided<br>*Only common tests should be described solely by name; describe more complex techniques in the Methods section.*
☒	☐	A description of all covariates tested
☒	☐	A description of any assumptions or corrections, such as tests of normality and adjustment for multiple comparisons
☐	☒	A full description of the statistical parameters including central tendency (e.g. means) or other basic estimates (e.g. regression coefficient) AND variation (e.g. standard deviation) or associated estimates of uncertainty (e.g. confidence intervals)
☒	☐	For null hypothesis testing, the test statistic (e.g. *F*, *t*, *r*) with confidence intervals, effect sizes, degrees of freedom and *P* value noted<br>*Give P values as exact values whenever suitable.*
☒	☐	For Bayesian analysis, information on the choice of priors and Markov chain Monte Carlo settings
☒	☐	For hierarchical and complex designs, identification of the appropriate level for tests and full reporting of outcomes
☒	☐	Estimates of effect sizes (e.g. Cohen's *d*, Pearson's *r*), indicating how they were calculated

Our web collection on statistics for biologists contains articles on many of the points above.

Software and code

Policy information about availability of computer code

Data collection	Images were collected using the Nikon, Leica and Biorad ChemiDocTM MP proprietary softwares as reported in the SI information file.
Data analysis	Images were collected using the built-in microscope software (NIS-Elements AR or Leica LAS X), processed and analyzed using FIJI and a custom developed Python software that is available on Github, as reported in the SI file S4.13. Model was developed using MATLAB (Mathworks). Software version information: NIS-Elements (4.20.02), Leica LAS X (3.5.6.21492), FIJI (2.1.0/1.53c), Python (3.7.6) via Anaconda (2020.02), scikit-image - Python package with CHT algorithm (0.16.2), nd2reader Python package (3.2.3), MATLAB (R202a and R2020b). Further dependencies are included on Github repository. Both Python and MATLAB scripts are available on Github and have a reference DOI (10.5281/zenodo.4763253): https://github.com/klockemel/dropletDetection_dynamicSelfAssemblyProject

For manuscripts utilizing custom algorithms or software that are central to the research but not yet described in published literature, software must be made available to editors and reviewers. We strongly encourage code deposition in a community repository (e.g. GitHub). See the Nature Research guidelines for submitting code & software for further information.

Data

Policy information about availability of data

All manuscripts must include a data availability statement. This statement should provide the following information, where applicable:

- Accession codes, unique identifiers, or web links for publicly available datasets
- A list of figures that have associated raw data
- A description of any restrictions on data availability

The data that support the findings of this study are available from the corresponding author upon reasonable request.

Field-specific reporting

Please select the one below that is the best fit for your research. If you are not sure, read the appropriate sections before making your selection.

- [x] Life sciences
- [ ] Behavioural & social sciences
- [ ] Ecological, evolutionary & environmental sciences

For a reference copy of the document with all sections, see nature.com/documents/nr-reporting-summary-flat.pdf

Life sciences study design

All studies must disclose on these points even when the disclosure is negative.

Sample size	No calculations for sample size were done. Sample sizes were determined by the number of droplets detected using the automated droplet detection Python code described in Supplementary Note S4.13. Sample sizes, dictated by the number of droplets detected per image (one image at each time point), were determined to be sufficient when they surpassed what we could have quantified by extracting values by hand in FIJI. The smallest sample size is 22 droplets (100 nM at 24 hrs, Supplementary Fig. S19 in Supplementary Note S5.9), while the majority of sample sizes are greater than 100 droplets.
Data exclusions	Data were excluded from the analyses in case of insufficient quality (out-of-focus, droplets whose edges were not detected by the in-house custom developed software, and samples in which many droplets were overlapping) as described in SI sections S4.13, S5.9.
Replication	Data which is quantified in the manuscript comes from single experiments, each including hundreds of droplets. Each experimental condition, however, was reproduced at least twice with some assays repeated three or more times for gathering of confocal data, control experiments, and optimizing imaging protocols.
Randomization	Randomization is not relevant to the study as the samples were not allocated into experimental groups.
Blinding	Blinding is not relevant to the study as no group allocation was performed.

Reporting for specific materials, systems and methods

We require information from authors about some types of materials, experimental systems and methods used in many studies. Here, indicate whether each material, system or method listed is relevant to your study. If you are not sure if a list item applies to your research, read the appropriate section before selecting a response.

Materials & experimental systems

n/a	Involved in the study	
☒	☐	Antibodies
☒	☐	Eukaryotic cell lines
☒	☐	Palaeontology and archaeology
☒	☐	Animals and other organisms
☒	☐	Human research participants
☒	☐	Clinical data
☒	☐	Dual use research of concern

Methods

n/a	Involved in the study	
☒	☐	ChIP-seq
☒	☐	Flow cytometry
☒	☐	MRI-based neuroimaging
