# Supplementary material for: Dynamic self-assembly of compartmentalized DNA nanotubes
Source: Nat Commun. 2021 Jun 11;12:3557. doi: 10.1038/s41467-021-23850-1 (PMC8196065; doi:10.1038/s41467-021-23850-1)
Supplement: Supplementary file 7 — Description of Additional Supplementary Files [file 41467_2021_23850_MOESM7_ESM.pdf]

Title: Supplementary Movie 1:
Description: This video shows the time lapse of the isothermal assembly process of the two-tile design nanotubes inside water-in-oil droplets in the imaging chamber at room temperature. The tiles are annealed in vitro separately and are mixed immediately before encapsulation (100 nM each tile with 1x TAE buffer and 12.5 mM $MgCl_2$, SI section 4.3).

Title: Supplementary Movie 2:
Description: This video shows the z-stack reconstruction of confocal images (images was captured using six line average) of a sample containing two-tile design nanotubes in water-in-oil droplets (100 nM each tile with 1x TAE buffer and 12.5 mM $MgCL_2$) in the imaging chamber at room temperature at 24 hours after encapsulation.

Title: Supplementary Movie 3:
Description: This video shows the time lapse of the isothermal assembly process of the DNA-RNA hybrid design nanotubes by in-situ RNA production inside water-in-oil droplets in the imaging chamber at 37°C. The inactive tiles are annealed separately and are mixed at 500 nM with the transcription mix (1x TXN buffer, 4 mM NTPs, 10 mM $MgCL_2$, 100 nM gene template, 2.5% w/v PEG and 2.5% v/v RNAP) immediately before encapsulation.

Title: Supplementary Movie 4:
Description: This video shows the time lapse of enzyme mediated control of assembly and disassembly of encapsulated hybrid DNA-RNA tubes inside water-in-oil droplets in the imaging chamber at 37°C. DNA template transcribing the RNA trigger promotes growth of nanotubes and RNase H promotes degradation of nanotubes. The inactive tiles are annealed separately and are mixed at 500 nM with the transcription mix (1x TXN buffer, 4 mM NTPs, 10 mM $MgCL_2$, 100 nM gene template, 2.5% w/v PEG, 2.5% v/v RNAP and 0.025 U/µl RNase H) immediately before encapsulation.
